# Two-year impact of community-based health screening and parenting groups on child development in Zambia: Follow-up to a cluster-randomized controlled trial

**DOI:** 10.1371/journal.pmed.1002555

**Published:** 2018-04-24

**Authors:** Peter C. Rockers, Arianna Zanolini, Bowen Banda, Mwaba Moono Chipili, Robert C. Hughes, Davidson H. Hamer, Günther Fink

**Affiliations:** 1 Department of Global Health, Boston University School of Public Health, Boston, Massachusetts, United States of America; 2 American Institutes for Research–Zambia Office, Lusaka, Zambia; 3 Zambia Center for Applied Health Research and Development, Lusaka, Zambia; 4 Department of Psychology, University of Zambia, Lusaka, Zambia; 5 UK Department for International Development, London, United Kingdom; 6 Section of Infectious Diseases, Department of Medicine, Boston Medical Center, Boston, Massachusetts, United States of America; 7 Swiss Tropical and Public Health Institute, Basel, Switzerland; 8 University of Basel, Basel, Switzerland; Makerere University Medical School, UGANDA

## Abstract

**Background:**

Early childhood interventions have potential to offset the negative impact of early adversity. We evaluated the impact of a community-based parenting group intervention on child development in Zambia.

**Methods and findings:**

We conducted a non-masked cluster-randomized controlled trial in Southern Province, Zambia. Thirty clusters of villages were matched based on population density and distance from the nearest health center, and randomly assigned to intervention (15 clusters, 268 caregiver–child dyads) or control (15 clusters, 258 caregiver–child dyads). Caregivers were eligible if they had a child 6 to 12 months old at baseline. In intervention clusters, caregivers were visited twice per month during the first year of the study by child development agents (CDAs) and were invited to attend fortnightly parenting group meetings. Parenting groups selected “head mothers” from their communities who were trained by CDAs to facilitate meetings and deliver a diverse parenting curriculum. The parenting group intervention, originally designed to run for 1 year, was extended, and households were visited for a follow-up assessment at the end of year 2. The control group did not receive any intervention. Intention-to-treat analysis was performed for primary outcomes measured at the year 2 follow-up: stunting and 5 domains of neurocognitive development measured using the Bayley Scales of Infant and Toddler Development–Third Edition (BSID-III). In order to show Cohen’s *d* estimates, BSID-III composite scores were converted to *z-*scores by standardizing within the study population. In all, 195/268 children (73%) in the intervention group and 182/258 children (71%) in the control group were assessed at endline after 2 years. The intervention significantly reduced stunting (56/195 versus 72/182; adjusted odds ratio 0.45, 95% CI 0.22 to 0.92; *p =* 0.028) and had a significant positive impact on language (β 0.14, 95% CI 0.01 to 0.27; *p =* 0.039). The intervention did not significantly impact cognition (β 0.11, 95% CI −0.06 to 0.29; *p =* 0.196), motor skills (β −0.01, 95% CI −0.25 to 0.24; *p =* 0.964), adaptive behavior (β 0.21, 95% CI −0.03 to 0.44; *p =* 0.088), or social-emotional development (β 0.20, 95% CI −0.04 to 0.44; *p =* 0.098). Observed impacts may have been due in part to home visits by CDAs during the first year of the intervention.

**Conclusions:**

The results of this trial suggest that parenting groups hold promise for improving child development, particularly physical growth, in low-resource settings like Zambia.

**Trial registration:**

ClinicalTrials.gov NCT02234726

## Abstract 2

In a two year follow-up of a cluster randomized trial, Peter Rockers and colleagues examine the longer term impact of a community-based health screening and parenting group intervention on child development in Zambia.

## Author summary

**Why was this study done?:**

- Prior studies have shown that community-based programs can have a positive impact on child physical growth and neurocognitive development.
- Our study was one of the first to test the impact of home visits and parenting groups on children in sub-Saharan Africa, where optimal child nutrition and stimulation are often lacking.

**What did the researchers do and find?:**

- We conducted a cluster-randomized controlled trial among households in rural Zambia.
- Caregivers of young children in intervention communities were visited at home for 1 year to monitor health and invited to attend fortnightly community-based group meetings over a 2-year period, where they learned a diverse parenting curriculum that included content on cognitive stimulation and play practices, child nutrition and cooking practices, and self-care for good mental health.
- We conducted a year 2 follow-up with 377 study children to assess the impact of the intervention on stunting and neurocognitive development.
- We found that the intervention reduced child stunting and had a positive impact on language development.

**What do these findings mean?:**

- Community-based early childhood interventions may be an effective platform for delivering parenting support in low-resource settings like Zambia.
- More research is needed to better understand how early childhood interventions can be integrated into existing public service systems to ensure sustainability.

## Introduction

Children in low- and middle-income countries continue to be exposed to a large number of risk factors affecting healthy development, ranging from exposure to malnutrition and infectious diseases to lack of appropriate stimulation and learning opportunities in their home environment and community [1,2]. According to the latest estimates, 40% of Zambian children under age 5 years are stunted and 6% are wasted [3]. More than 60% of the country’s population lives below the national poverty line, and the under-5 mortality rate is 64 per 1,000 live births [4]. Only 32% of Zambian children receive any form of early childhood care and education by age 6 years [5]. Efforts are currently underway to address the country’s malnutrition burden, most notably through the Scaling Up Nutrition funding mechanism [6].

While developmental deficits early in life have been shown to persist well into adulthood [7], a growing body of evidence suggests that the negative impact of early adversity in the short and medium run can be mitigated through appropriate early-life interventions [8]. In several settings, coaching caregivers on appropriate play-based activities and providing nutrition counseling have been shown to improve physical growth and neurocognitive development [9–11]. Community-based parenting groups have shown promising results in a few settings, and may be an effective and low-cost platform for delivering interventions to improve child health and development [12–17]. A set of recent studies in Uganda have provided evidence of the positive potential of community-based parenting interventions for improving child development in sub-Saharan Africa [18–20].

We conducted a cluster-randomized controlled trial to test the impact of a community-based parenting group intervention in a rural area of Southern Province, Zambia. As part of the trial, households in communities randomized to the intervention were invited to attend fortnightly parenting group meetings covering a diverse curriculum with content on cognitive stimulation, child nutrition, and self-care. The full intervention period lasted 2 years. An initial assessment of the intervention after 12 months found positive changes in caregiver behavior, but did not show statistically significant impacts on developmental outcomes [21]. In this paper we present the results of a year 2 in-depth follow-up of the original study cohort.

## Methods

### Study design and setting

The study was a cluster-randomized controlled trial implemented in the catchment areas of 5 health facilities in Choma and Pemba districts, Southern Province, Zambia.

### Participants

Households were randomly selected in 3 stages of sampling. In the first stage, 5 rural health centers (RHCs) were purposefully selected. These RHCs were part of a previous research study conducted by the study team [22]. In the second stage, 6 health zones were randomly selected from each of the 5 health centers. In the third stage, villages were randomly selected with probability proportional to size to reach the target sample of 18 eligible caregiver–child dyads in each health zone. The aim was to enroll 524 dyads in the study at baseline. Villages with fewer than 2 eligible children were excluded to ensure a sufficient number of potential participants in group meetings. In selected villages, all households were screened for eligibility. Eligible households were provided with study information as part of the informed consent procedure and decided whether to enroll at that time.

To be eligible for the study, a household had to have a child between 6 and 12 months of age at the time of enrollment. Caregivers reported their child’s birthdate during eligibility screening, and the reported date was confirmed by reviewing the child’s health card when available; 96% of households had the child health card for review. Caregivers younger than 15 years of age were excluded. All caregivers provided informed consent prior to study initiation. The study was approved by the Institutional Review Board at Boston University (protocol number H-32726) and by the ethics board at ERES Converge in Zambia (protocol number 2013-Dec-010) prior to the enrollment of participants.

The initial target sample size of 524 caregiver–child dyads was powered to detect a 0.5–standard deviation (SD) difference in standardized child development scores with 90% power, assuming 30 clusters of equal size, 5% loss to follow-up, and an intracluster correlation coefficient of 0.1.

### Randomization and masking

Health zones (clusters) were randomized prior to baseline enrollment with equal probability to either the intervention or the control group. Prior to randomization, health zones were matched in pairs within RHC catchment areas based on distance to the health center and village population. Within each matched pair, 1 health zone was randomly assigned to the intervention group. Group assignment was masked from all assessors, but masking of participants was not possible.

### Procedures

The original study was funded for 1 year, and all study activities stopped at the end of that year, in November 2015. The study team secured funding for a second-year extension at the end of 2015, and study activities resumed in March 2016, at which time households were also re-consented to participate.

During the original 1-year study, intervention households received 2 services. First, they received a fortnightly visit by a child development agent (CDA), a community-based health worker employed full time by the project. During home visits, CDAs screened and referred children for infections and acute malnutrition, and encouraged caregivers to use routine child health services. Second, caregivers were invited to attend fortnightly parenting group meetings, where they were taught a diverse curriculum that included content on cognitive stimulation and play practices, child nutrition and cooking practices, and self-care for good mental health. During the year 2 study extension, the household visit component of the intervention was dropped, while facilitation of the fortnightly parenting group meetings continued. This change was motivated by the findings from the year 1 assessment, which suggested that the parenting groups were the primary driver of observed positive behavior change [21].

CDAs facilitated the organization of fortnightly parenting group meetings in intervention communities. Each health zone had 2 CDAs who each covered half of its communities. CDAs were selected through consultation with communities, and all had previous experience providing community-based health services, some as part of the formal health system. Prior to the start of the study, CDAs were trained on how to support group meetings. A local child development curriculum, containing age-appropriate activities covering health, nutrition, and early stimulation activities, as well as content related to caregiver mental health, was developed for these meetings. The curriculum was newly developed for the local context by the research team and included elements from existing child development programs, including the Care for Child Development package [23] and the Essential Package from Care International [24]. Content from these existing packages was adapted using an interactive theater approach. The curriculum training manual is provided in S1 Text. During the study period, CDAs were trained on the curriculum 3 meeting rounds at a time every 6 weeks. Groups were also encouraged to meet during intervening weeks without a formal curriculum. Meetings were held at multiple sites within each health zone. Meeting locations were chosen by group members to minimize travel distances. Each group meeting was run by a local “head mother” who was selected by the members of the group. CDAs met with head mothers before each round of meetings and provided them with training and resources according to the planned curriculum for that round. CDAs did not regularly attend the meetings themselves. Each meeting focused on a different topic; the topics included child nutrition, forms of play, cognitive stimulation, and language activities. All female caregivers in study communities with children under 5 years of age were invited to attend meetings. Caregivers were encouraged to bring children to group meetings, as aspects of the curriculum involved interactions with children. Based on consultation with local community leaders, male caregivers were not invited to attend group meetings.

Household survey data were collected at 4 time points: baseline (August/September 2014), year 1 follow-up (October/November 2015), re-consent (March 2016), and year 2 follow-up (November/December 2016). In this paper, we focus on data collected at baseline, re-consent, and the year 2 follow-up; a full analysis of data from the year 1 follow-up has been published previously [21]. Baseline, year 1 follow-up, and re-consent data collection was conducted at children’s homes. To ensure a controlled environment for the administration of the Bayley Scales of Infant and Toddler Development–Third Edition (BSID-III) at the year 2 follow-up, caregivers were invited to bring their child to the nearest health center, where the assessment was conducted one child at a time in a private room.

### Outcomes

Primary outcomes were children’s stunting (defined as height-for-age *z-*score [HAZ] < −2) and neurocognitive development. Children’s height and weight were measured by trained assessors at baseline and the year 2 follow-up using standard anthropometric assessment kits. Length of all children under 2 years of age was collected in a lying position; height of older children was measured in a standing position. Length and height boards were rented from the Zambia National Food and Nutrition Commission (NFNC). Child weight was measured using digital scales also rented from the NFNC. Study interviewers were carefully trained for the anthropometric assessment, and a member of the NFNC attended the training to ensure correct use of the boards and scales. Anthropometric data were converted to *z-*scores using WHO child growth standards [25]. Child neurocognitive development was assessed using the BSID-III, which has previously been used and validated in Zambia [26]. A team of assessors attended a 2-week training on the BSID-III led by an accredited trainer prior to the start of data collection. Following the training, assessors spent 1 week pilot testing assessment procedures in the field. The BSID-III assesses 5 domains of development: cognition, language, motor, adaptive behavior, and social-emotional. Composite scores were determined for each domain using Bayley-III Scoring Assistant software [27] according to a 3-step procedure: first, raw scores were established by summing the number of items successfully completed for each sub-domain; second, scaled scores were constructed for each sub-domain by age-standardizing raw scores using the BSID-III reference population and rescaling to a range of 1 to 19, a mean of 10, and a standard deviation of 3; and third, composite scores were constructed by summing sub-domain scaled scores within domains and rescaling to a range of 40 to 160, a mean of 100, and standard deviation of 15. In order to show Cohen’s *d* estimates, composite scores were then converted to *z-*scores by standardizing within the study population.

Other outcome measures collected included HAZ, caregiver–child interactions, caregiver mental health, and child diet. Data on caregiver–child interactions were collected at baseline, year 1 follow-up, re-consent, and year 2 follow-up, using the 6-item Multiple Indicator Cluster Survey (MICS) module [28]. For child diet, a diet diversity score was constructed, using year 2 data, based on the number of food groups children had consumed the previous day, as per the method described in Steyn et al. [29]. Caregiver mental health was assessed using the 20-item WHO Self Reporting Questionnaire (SRQ) [30]. Caregiver depression was defined as reporting 7 or more symptoms on the SRQ, as per standard procedure [31]. Household demographics and asset information were collected at baseline. Caregivers reported on ongoing attendance at group meetings in the year 2 follow-up survey.

### Statistical analysis

First, we compare baseline characteristics of the intervention and control groups, and present data on attrition by treatment arm. We then estimate the impact of the intervention on the primary outcomes of interest using intention-to-treat analysis. For continuous outcome variables, ordinary least squares regression models were fit to estimate unadjusted and adjusted impacts (β estimates). For dichotomous outcome variables, logistic regression models were fit to estimate unadjusted and adjusted odds ratios (ORs). Unadjusted models included controls for the cluster matching variables (population size and distance from the nearest health center) and, when available, the outcome variable measured at baseline. Adjusted models included a set of controls selected according to the following procedure: first, the outcome measured at the year 2 follow-up was regressed on the outcome measured at baseline, when available, and a set of demographic characteristics measured at baseline following a backward stepwise selection process with a drop threshold set at *p =* 0.2. The baseline demographic variable set included child HAZ, child weight-for-age *z-*score, child mid-upper arm circumference, child age, child sex, caregiver age, caregiver’s education, father’s education, and child motor skills according to the CREDI Scale [32]. Interviewer fixed effects were also included in the stepwise selection models. The resulting subset of variables from each model was included in the adjusted model for each outcome. For all models, standard errors were adjusted to account for clustering.

As a robustness check on the primary results, we conducted a per-protocol analysis, stratifying the treatment group according to household attendance at parenting groups. We also present data on caregiver–child interactions at each round of data collection, including the re-consent visit after a 5-month interruption in the delivery of the intervention. Finally, we estimate the impact of the intervention on other outcomes of interest, including caregiver mental health and child diet diversity. Cluster-robust standard errors were used to account for within cluster correlation. All analyses were conducted using Stata statistical software [33]. This trial was registered on ClinicalTrials.gov prior to baseline data collection (NCT02234726). Data were deposited in the Dryad repository: https://doi.org/10.5061/dryad.3340hc4 [34].

## Results

### Study population

**Fig 1:**
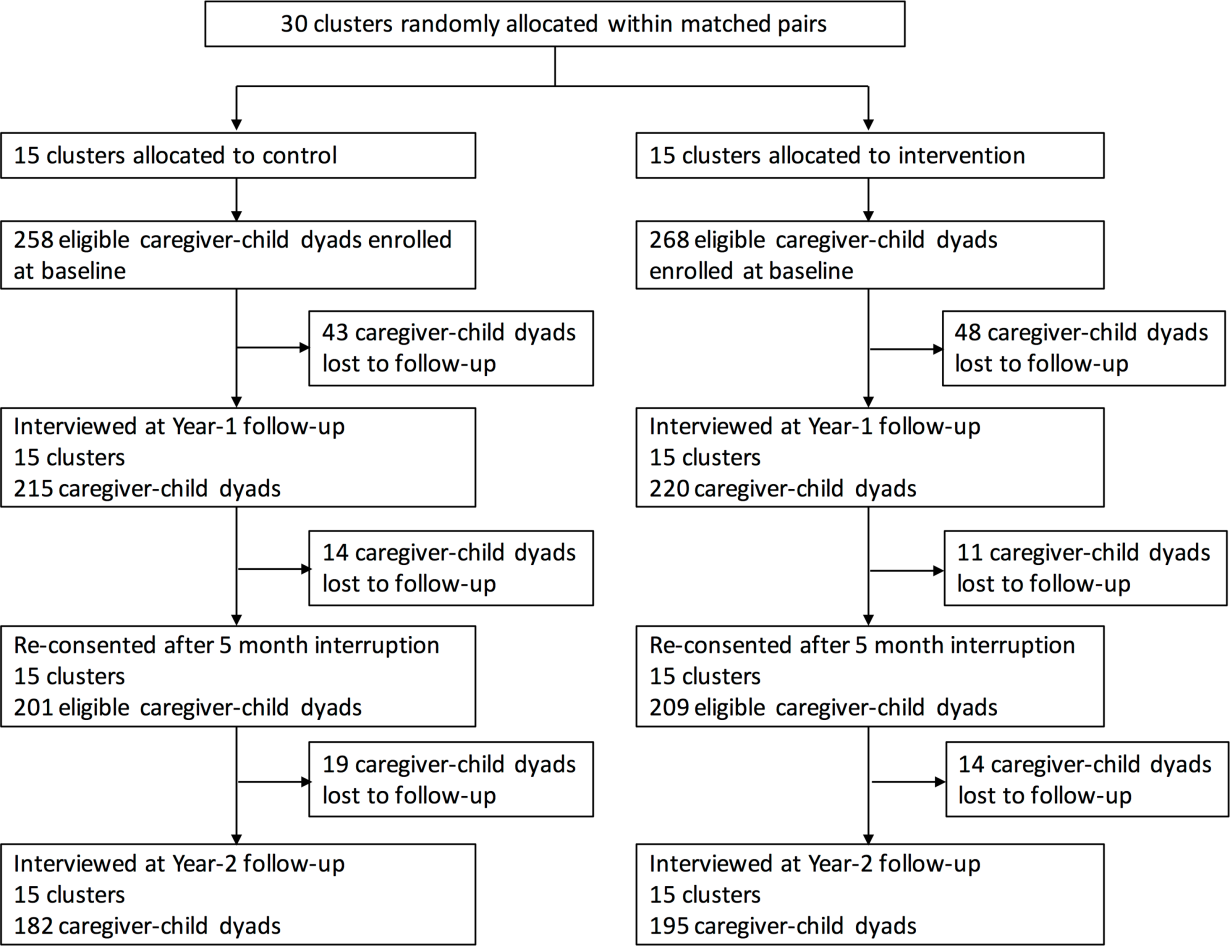
Trial profile.

Thirty clusters were randomly assigned to intervention (15 clusters, 268 caregiver–child dyads) or control (15 clusters, 258 caregiver–child dyads). Overall, 5% of eligible households approached at enrollment refused to participate in the study. During the first year of the study, 48 dyads in the intervention group and 43 dyads in the control group were lost to follow-up. After a 5-month interruption in the study intervention, 209 intervention dyads and 201 control dyads were re-consented to participate in the second year of the study. At the end of the second year (last visit December 23, 2016), 195 caregiver–child dyads (73%) remained in the intervention group, while 182 dyads (71%) remained in the control group (Fig 1). Baseline characteristics of enrolled caregivers and children were similar in the intervention and control groups (Table 1). Nearly all (97%) caregivers in the study population were the child’s mother.

**Table 1:** Baseline characteristics of study participants.

Characteristic	Control group			Intervention group		
	All enrolled (*n =* 258)	Remaining at year 2 follow-up (*n =* 182)	Lost to follow-up (*n =* 76)	All enrolled (*n =* 268)	Remaining at year 2 follow-up (*n =* 195)	Lost to follow-up (*n =* 73)
**Child characteristics at baseline**						
Age (months), mean (SD)	8.50 (2.03)	8.53 (2.05)	8.38 (2.03)	8.74 (2.17)	8.74 (2.21)	8.74 (2.06)
Height-for-age *z-*score, mean (SD)	−1.52 (1.60)	−1.52 (1.60)	−1.49 (1.70)	−1.63 (1.75)	−1.62 (1.85)	−1.68 (1.67)
Weight-for-age *z-*score, mean (SD)	−0.31 (1.38)	−0.20 (1.42)	−0.54 (1.28)	−0.45 (1.38)	−0.50 (1.44)	−0.32 (1.24)
Weight-for-height *z-*score, mean (SD)	0.76 (1.77)	0.87 (1.75)	0.51 (1.79)	0.74 (2.01)	0.68 (2.04)	0.89 (1.95)
Female, *n* (%)	131 (50.8)	94 (51.7)	37 (48.7)	126 (47.0)	93 (47.7)	33 (45.2)
Stunting, *n* (%)	89 (35.0)	59 (33.0)	30 (40.0)	102 (40.5)	74 (40.7)	28 (40.0)
Underweight, *n* (%)	30 (11.7)	21 (11.5)	9 (11.8)	39 (14.6)	32 (16.5)	7 (9.6)
Wasting, *n* (%)	13 (5.2)	8 (4.5)	5 (6.7)	22 (8.8)	16 (8.9)	6 (8.6)
Diarrhea in the previous 2 weeks, *n* (%)	86 (33.7)	60 (33.2)	26 (35.1)	83 (31.0)	66 (33.9)	17 (23.3)
Fever in the previous 2 weeks, *n* (%)	81 (31.8)	54 (29.8)	27 (36.5)	75 (28.2)	60 (31.1)	15 (20.6)
Cough in the previous 2 weeks, *n* (%)	119 (46.7)	91 (50.3)	28 (37.8)	107 (39.9)	78 (40.0)	29 (39.7)
**Caregiver characteristics at baseline**						
Age (years), mean (SD)	27.6 (8.94)	28.1 (9.57)	26.3 (7.41)	27.0 (7.75)	27.7 (7.79)	25.1 (8.09)
Mental health (SRQ score), mean (SD)	4.57 (3.45)	4.65 (3.39)	4.36 (3.62)	3.72 (2.89)	3.75 (2.81)	3.64 (3.11)
Household wealth quintile, mean (SD)	2.85 (1.42)	2.89 (1.44)	2.76 (1.36)	3.13 (1.41)	3.26 (1.40)	2.78 (1.38)
Completed primary school, *n* (%)	169 (66.3)	123 (68.0)	46 (62.2)	182 (70.0)	128 (67.7)	54 (76.1)

Child height-for-age *z-*score, weight-for-age *z-*score, and weight-for-height *z-*score are normalized to WHO standards. Stunting is defined as height-for-age *z-*score < −2. Underweight is defined as weight-for-age *z-*score < −2. Wasting is defined as weight-for-height *z-*score < −2. Lower SRQ score indicates better mental health.

SRQ, Self Reporting Questionnaire.

Children were on average 8 months old at the start of the study, with a relatively uniform distribution within the eligible age range (6 to 12 months). Height was on average well below the international reference median, with a mean HAZ of −1.5 at baseline. More than one-third of children were stunted at baseline. Study caregivers were on average 27 years old at baseline, and slightly fewer than half had completed primary school.

### Impact of intervention on primary outcomes

**Fig 2:**
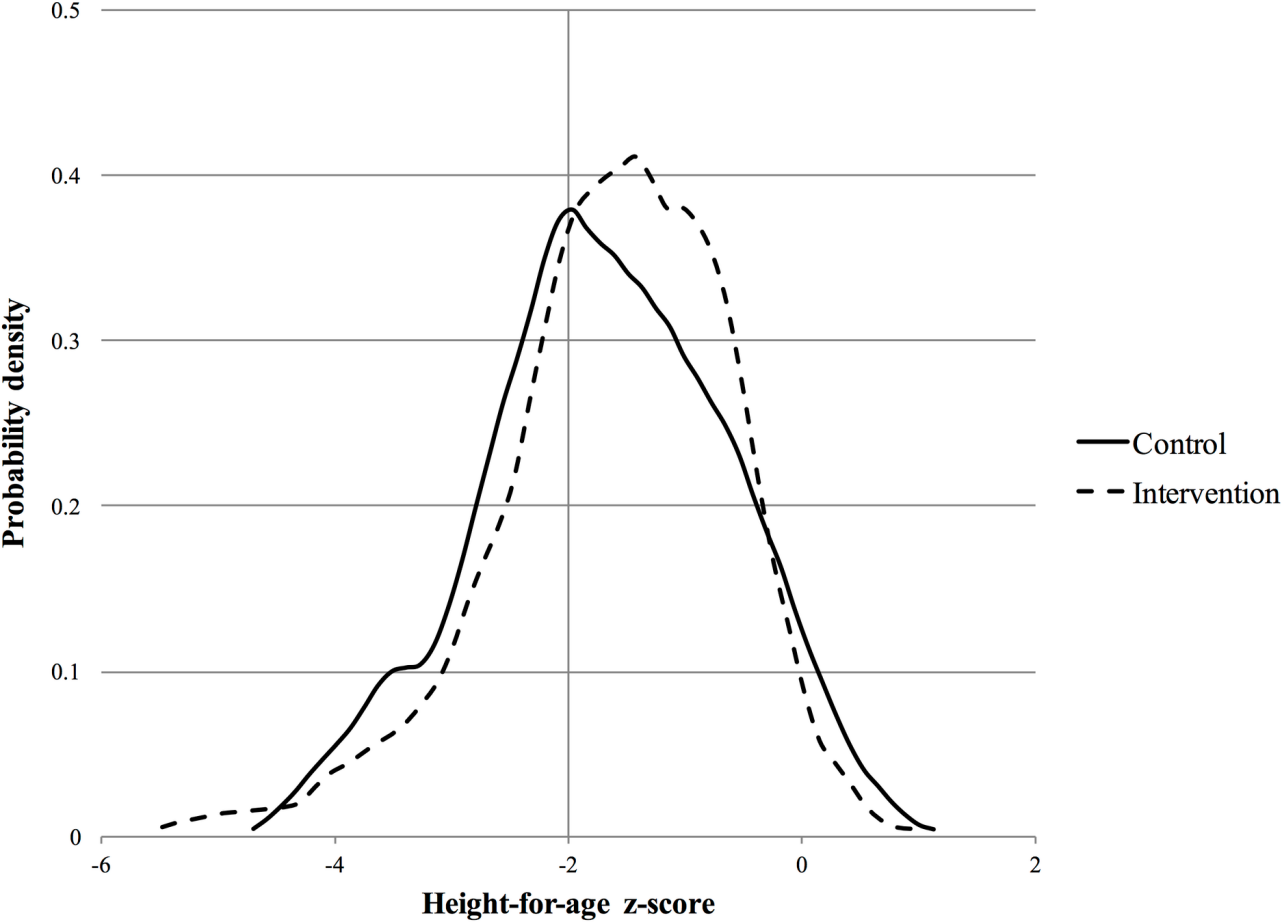
Probability density function for height-for-age *z-*score at year 2 follow-up.

Controlling for a set of baseline characteristics (Table 2), the intervention significantly reduced the odds of stunting (OR 0.45 [95% CI 0.22 to 0.92]; *p =* 0.028). Probability density functions for HAZ at the year 2 follow-up in the intervention and control groups are presented in Fig 2. Corresponding cumulative density functions are provided in S1 Fig. The intervention had a significant positive impact on child language (β 0.14 [95% CI 0.01 to 0.27]; *p =* 0.039). There was no impact on cognition (β 0.11 [95% CI −0.06 to 0.29]; *p =* 0.196), motor skills (β −0.01 [95% CI −0.25 to 0.24]; *p =* 0.964), adaptive behavior (β 0.21 [95% CI −0.03 to 0.44]; *p =* 0.088), or social-emotional development (β 0.20 [95% CI −0.04 to 0.44]; *p =* 0.098). We found no evidence to suggest that physical growth mediates these impacts (S1 Table). Raw and scaled BSID-III subtest scores are provided in S2 Table.

**Table 2:** Impact of the intervention on primary outcomes at year 2 follow-up.

Outcome	*n* (%) or mean (SD) at year 2 follow-up^a^		ICC	Unadjusted^b^		Adjusted^c^	
	Control	Intervention		OR or β^d^ (95% CI)	*p-*Value	OR or β^d^ (95% CI)	*p-*Value
**Stunting**	72 (39.6)	56 (29.2)	0.07	0.53 (0.30, 0.94)	0.029	0.45 (0.22, 0.92)	0.028
**BSID-III *z-*scores**							
Cognition	0.01 (1.02)	−0.01 (0.98)	0.06	0.07 (−0.14, 0.28)	0.510	0.11 (−0.06, 0.29)	0.196
Language	0.04 (0.97)	−0.04 (1.03)	0.04	−0.02 (−0.24, 0.19)	0.818	0.14 (0.01, 0.27)	0.039
Motor	0.05 (0.93)	−0.05 (1.06)	0.07	−0.03 (−0.26, 0.20)	0.782	−0.01 (−0.25, 0.24)	0.964
Adaptive behavior	−0.14 (0.99)	0.13 (1.00)	0.05	0.28 (0.06, 0.49)	0.014	0.21 (−0.03, 0.44)	0.088
Social-emotional	−0.13 (1.08)	0.12 (0.90)	0.06	0.28 (0.05, 0.51)	0.019	0.20 (−0.04, 0.44)	0.098

β estimates for all BSID-III *z-*scores are equivalent to Cohen’s *d* values. Stunting is defined as height-for-age *z-*score < −2. All standard errors are adjusted to account for clustering.

^a^Stunting data are summarized as *n* (%). BSID-III *z*-score data are summarized as mean (SD).

^b^Controlling for randomization blocking variables (cluster population and distance to nearest health center) and baseline value of the outcome (not available for BSID-III outcomes).

^c^Controlling for randomization blocking variables (cluster population and distance to nearest health center), a set of baseline demographic variables correlated with the outcome at the year 2 follow-up determined according to a backward stepwise selection procedure, and baseline value of the outcome (not available for BSID-III outcomes).

^d^Impact on stunting is presented as an OR estimate. Impacts on BSID-III *z*-scores are presented as β estimates.

BSID-III, Bayley Scales of Infant and Toddler Development–Third Edition; ICC, intracluster correlation coefficient; OR, odds ratio.

In S2 Fig, we show reported attendance at parenting group meetings. In total, there were 27 parenting groups in intervention communities, and on average 6 caregivers attended each meeting. A majority of caregivers reported attending parenting group meetings either 2 to 3 (22%) or 4 or more times (35%) per month, indicating that many groups took the initiative to meet on their own in the time between fortnightly planned program meetings. Around one-third of caregivers in the intervention group reported that by the end of the study period they did not attend meetings. In Table 3, we present a per-protocol analysis. Treatment effects were significant for stunting, language, and adaptive behavior among compliers, i.e., those who attended 2 or more group meetings per month; no significant impacts were found among non-compliers.

**Table 3:** Per-protocol analysis of impact of the intervention on primary outcomes.

Outcome	Compliers^a^ (*n =* 112)		Non-compliers (*n =* 76)	
	OR or β^b^ (95% CI)	*p-*Value	OR or β^b^ (95% CI)	*p-*Value
**Stunting**	0.44 (0.20, 0.93)	0.032	0.48 (0.20, 1.12)	0.091
**BSID-III *z-*scores**				
Cognition	0.16 (−0.06, 0.37)	0.160	0.08 (−0.19, 0.35)	0.555
Language	0.24 (0.02, 0.45)	0.034	−0.02 (−0.21, 0.17)	0.817
Motor	0.01 (−0.32, 0.35)	0.935	−0.05 (−0.31, 0.20)	0.676
Adaptive behavior	0.31 (0.01, 0.61)	0.046	0.00 (−0.26, 0.27)	0.972
Social-emotional	0.22 (−0.03, 0.47)	0.081	0.21 (−0.07, 0.50)	0.136

β estimates for all BSID-III *z-*scores are equivalent to Cohen’s *d* values. Stunting is defined as height-for-age *z-*score < −2. All standard errors are adjusted to account for clustering. All models include controls for randomization blocking variables (cluster population and distance to nearest health center), a set of baseline demographic variables correlated with the outcome at the year 2 follow-up determined according to a backward stepwise selection procedure, and baseline value of the outcome (not available for BSID-III outcomes).

^a^Compliers are defined as households that reported attending 2 or more parenting group meetings per month at the year 2 follow-up. Data on compliance were missing for 7 intervention households.

^c^Impact on stunting is presented as an OR estimate. Impacts on BSID-III *z*-scores are presented as β estimates.

BSID-III, Bayley Scales of Infant and Toddler Development–Third Edition; OR, odds ratio.

### Caregiver–child interactions

At baseline, no differences were found in reports of caregiver–child interactions (Table 4). The intervention had a positive impact on reported interactions at year 1 follow-up (0.70 SD [95% CI 0.51 to 0.89]; *p* < 0.001). At the re-consent visit, at the end of a 5-month interruption in the delivery of the intervention, reported interactions remained significantly higher in the intervention group (0.71 SD [95% CI 0.52 to 0.90]; *p* < 0.001). Significant but somewhat smaller differences were found at year 2 follow-up (0.35 SD [95% CI 0.12 to 0.59]; *p =* 0.005). Caregiver–child interactions at each survey round are summarized in S3 Table.

**Table 4:** Caregiver–child interaction at each period of data collection.

Time point	Mean (SD) caregiver–child interaction *z-*score^a^		ICC	Unadjusted^b^		Adjusted^c^	
	Control	Intervention		β (95% CI)	*p-*Value	β (95% CI)	*p-*Value
Baseline	0.00 (1.04)	0.00 (0.96)	0.32	0.00 (−0.50, 0.50)	0.999	0.00 (−0.42, 0.43)	0.987
Year 1 follow-up	−0.37 (0.93)	0.35 (0.94)	0.13	0.74 (0.54, 0.93)	<0.001	0.70 (0.51, 0.89)	<0.001
Re-consent	−0.36 (1.07)	0.33 (0.80)	0.46	0.68 (0.20, 1.16)	0.007	0.71 (0.52, 0.90)	<0.001
Year 2 follow-up	−0.21 (1.00)	0.19 (0.97)	0.09	0.42 (0.16, 0.67)	0.002	0.35 (0.12, 0.59)	0.005

All standard errors are adjusted to account for clustering.

^a^Caregiver–child interaction *z-*score was computed by standardizing the raw score within the study population for each round of data collection separately. The raw score was equal to the number of Multiple Indicator Cluster Survey questions to which the caregiver answered “yes,” out of a total possible score of 6.

^b^Controlling for randomization blocking variables (cluster population and distance to nearest health center) and baseline value of the outcome (not available for baseline caregiver–child interaction *z-*score).

^c^Controlling for randomization blocking variables (cluster population and distance to nearest health center), a set of baseline demographic variables correlated with the outcome at the year 2 follow-up determined according to a backward stepwise selection procedure, and baseline value of the outcome (not available for baseline caregiver–child interaction *z-*score).

ICC, intracluster correlation coefficient.

### Impact of the intervention on other secondary outcomes

Caregivers in the intervention group had slightly lower odds of depression (OR 0.63 [95% CI 0.31 to 1.26]; *p =* 0.193) at the year 2 follow-up, though these results were nonsignificant (Table 5). The intervention did not significantly impact HAZ (0.15 SD [95% CI −0.12 to 0.41]; *p =* 0.266) or child diet diversity score (0.20 [95% CI −0.13 to 0.53]; *p =* 0.218). Child diet data are summarized in S3 Table.

**Table 5:** Impact of the intervention on secondary outcomes at year 2 follow-up.

Outcome	Mean (SD) or *n* (%) at year 2 follow-up^a^		ICC	Unadjusted^b^		Adjusted^c^	
	Control	Intervention		β or OR^d^ (95% CI)	*p-*Value	β or OR^d^ (95% CI)	*p-*Value
Child height-for-age *z-*score (SD)	−1.70 (1.03)	−1.63 (0.99)	0.05	0.10 (−0.18, 0.38)	0.463	0.15 (−0.12, 0.41)	0.266
Child diet diversity score^e^	4.96 (1.21)	5.22 (1.18)	0.09	0.29 (−0.05, 0.64)	0.089	0.20 (−0.13, 0.53)	0.218
Caregiver depression (SRQ score > 7)	47 (25.8)	29 (14.9)	0.01	0.55 (0.34, 0.89)	0.016	0.63 (0.31, 1.26)	0.193

All standard errors are adjusted to account for clustering.

^a^Child height-for-age *z*-score and diet diversity score data are summarized as mean (SD). Caregiver depression data are summarized as *n* (%).

^b^Controlling for randomization blocking variables (cluster population and distance to nearest health center) and baseline value of the outcome (not available for child diet diversity).

^c^Controlling for randomization blocking variables (cluster population and distance to nearest health center), a set of baseline demographic variables correlated with the outcome at the year 2 follow-up determined according to a backward stepwise selection procedure, and baseline value of the outcome (not available for child diet diversity).

^d^Impacts on child height-for-age *z*-score and diet diversity score are presented as β estimates. Impact on caregiver depression is presented as an OR estimate.

^e^Number of food groups out of a possible of 7 consumed in previous 24 hours.

ICC, intracluster correlation coefficient; OR, odds ratio; SRQ, Self Reporting Questionnaire.

## Discussion

We investigated the impact of community-based parenting groups on child development in Southern Province, Zambia. After 2 years, the intervention substantially reduced the odds of stunting and improved child language development. The intervention had a positive impact on caregiver–child interaction, which appears to have persisted during a 5-month interruption in delivery of the intervention.

The large reductions in stunting are somewhat surprising given the low intensity of the intervention and also the minimal changes in diet observed. The small differences in diet may be partly explained by the seasonal timing of the year 2 follow-up assessment, which was conducted in December, in the middle of what is known in Zambia as the “hungry season,” when food reserves from the most recent harvest are mostly exhausted and households tend to reduce their overall nutritional intake and diet diversity. Significant impacts on diet diversity were observed at the year 1 follow-up, which was conducted in October, when food reserves are slightly more abundant [21]. While seasonal impacts on diet would affect both treatment groups, intervention impact estimates are likely smaller in settings where external constraints reduce food diversity to a minimum. The fact that a high density of study children was found to have HAZ near the stunting threshold may have contributed to the large impact on stunting; relatively modest and nonsignificant improvements in HAZ appear to have moved a significant number of children to just above the threshold.

While changes in parenting behavior were observed at the year 1 follow-up, only small, nonsignificant impacts on child development were found at that time [21]. Child physical growth and neurocognitive development are the results of complex and cumulative processes, with differentials resulting from early adversity often becoming more pronounced over time. It stands to reason that positive impacts from child development interventions might also develop over time. The temporal aspect of child development is important to consider when designing future studies, as longer intervention periods and follow-up may be needed to realize potential impacts. In addition, the utilization of BSID-III as a more detailed tool (rather than the INTERGROWTH-21st Neurodevelopment Assessment [INTER-NDA] [35] used at the year 1 follow-up) undoubtedly also increased our ability to detect developmental differences, which appear to have been domain-specific.

There are several key limitations to this work. First, the intervention package changed after the first year of the study, when household visits were stopped. We cannot say for certain that the impacts observed at the year 2 follow-up are entirely the result of parenting groups. However, the parenting group curriculum was the only platform for providing caregivers with nutrition and child stimulation information; therefore, it is very likely that this was the primary mechanism driving observed impacts. This conclusion is supported by the finding that in general larger impacts were found among children in complier households. Second, the delivery of the intervention relied on a cadre of CDAs operating parallel to the existing health system. Without significant additional public resources, scale-up efforts would likely require a delivery platform that is integrated into existing structures, such as Safe Motherhood Action Groups [36] or community health workers delivering integrated community case management [37]. Third, our measurement of parenting behavior was largely dependent on self-report, and parents may have had reasons to misreport their own behavior, introducing a source of potential bias in our behavior change estimates. This concern does not, however, apply to our primary outcomes, which were directly observed and measured by trained study staff. Fourth, we tested the impact of the intervention on 6 primary outcomes, raising concerns around multiple hypothesis testing. With Bonferroni corrections, only the estimated impact on stunting would be statistically significant, while the impact on language development would be nonsignificant. With the relatively small sample size used in this study, large effect sizes would be needed to yield *p*-values below the corrected significance threshold. Lastly, nearly 30% of households enrolled at baseline were lost to the study at the year 2 follow-up, which may have introduced some bias in our impact estimates. However, only 1 statistically significant difference was found between those retained and those lost to follow-up: in the control group, cough at baseline was more likely in the retained group (*p =* 0.03). Given the number of variables tested, 1 significant association is expected by chance. As in most low- and middle-income countries, migration from rural to urban areas in search of economic opportunities is common in Zambia and may explain the observed attrition rates [38].

This paper adds to a growing body of literature on the delivery of early childhood interventions in low-resource settings [17]. Community-based parenting groups appear to be a feasible and effective means of reaching a large number of households that may otherwise not be able to access center-based services. In settings like Zambia, where optimal child nutrition and stimulation are often lacking, parenting groups hold promise for improving child health and welfare. However, improvements in child development may not be immediate, and continued and sustained efforts are likely needed.

## Supporting information

S1 Fig Cumulative density function for height-for-age *z-*score at year 2 follow-up. (DOCX)

S2 Fig Reported attendance at parenting group meetings at time of year 2 follow-up. (DOCX)

S1 Table Analysis of physical growth as a mediating variable. (DOCX)

S2 Table Raw and scaled neurodevelopment subtest scores. (DOCX)

S3 Table Summary of MICS and child diet data. (DOCX)

S1 Text Parenting group curriculum training manual. (PDF)

S2 Text Study protocol. (DOCX)

S3 Text CONSORT checklist. (DOCX)

## Abbreviations

BSID-III: Bayley Scales of Infant and Toddler Development–Third Edition
CDA: child development agent
HAZ: height-for-age *z-*score
MICS: Multiple Indicator Cluster Survey
RHC: rural health center
SD: standard deviation
SRQ: Self Reporting Questionnaire
