# Supplementary material for: Two-year impact of community-based health screening and parenting groups on child development in Zambia: Follow-up to a cluster-randomized controlled trial
Source: PLoS Med. 2018 Apr 24;15(4):e1002555. doi: 10.1371/journal.pmed.1002555 (PMC5915271; doi:10.1371/journal.pmed.1002555)
Supplement: S1 Table — (DOCX) [file pmed.1002555.s003.docx]

|  | **Controlling for height-for-age z-score at year two follow-up** | | **Controlling for caregiver-child interaction z-score at year two follow-up** | |
| --- | --- | --- | --- | --- |
|  | **β (95% CI)** | **p value** | **β (95% CI)** | **p value** |
| **BSID-III z-scores** |  |  |  |  |
| Cognition | 0.10 (-0.06, 0.27) | 0.213 | 0.08 (-0.09, 0.25) | 0.358 |
| Language | 0.13 (0.04, 0.23) | 0.044 | 0.10 (-0.02, 0.23) | 0.103 |
| Motor | -0.02 (-0.26, 0.22) | 0.882 | 0.00 (-0.26, 0.25) | 0.976 |
| Adaptive behaviour | 0.21 (-0.03, 0.45) | 0.090 | 0.08 (-0.11, 0.28) | 0.397 |
| Social-emotional | 0.21 (-0.05, 0.47) | 0.104 | 0.15 (-0.07, 0.36) | 0.178 |

*Notes:* β estimates for all BSID-III z-scores are equivalent to *Cohen’s d* values. All standard errors are adjusted to account for clustering. All models include controls for randomization blocking variables (cluster population and distance to nearest health facility), a set of baseline demographic variables correlated with the outcome at the two year follow-up, determined according to a backward stepwise selection procedure.
