# Supplementary material for: Two-year impact of community-based health screening and parenting groups on child development in Zambia: Follow-up to a cluster-randomized controlled trial
Source: PLoS Med. 2018 Apr 24;15(4):e1002555. doi: 10.1371/journal.pmed.1002555 (PMC5915271; doi:10.1371/journal.pmed.1002555)
Supplement: S2 Table — (DOCX) [file pmed.1002555.s004.docx]

| **Bayley Scale for Infant and Toddler**  **Development (BSID-III) subtest** | **Raw scores** | |  | **Scaled scores** | |
| --- | --- | --- | --- | --- | --- |
|  | **Control** | **Intervention** |  | **Control** | **Intervention** |
| Cognitive | 64.99 | 65.10 |  | 6.77 | 6.72 |
| Expressive Language | 33.76 | 33.31 |  | 8.07 | 8.02 |
| Receptive Language | 32.64 | 31.93 |  | 9.15 | 8.83 |
| Gross Motor | 61.32 | 60.52 |  | 9.54 | 8.92 |
| Fine Motor | 44.63 | 44.58 |  | 8.35 | 8.45 |
| Adaptive Behavior - Communication | 53.15 | 54.51 |  | 7.86 | 8.09 |
| Adaptive Behavior – Community Use | 36.92 | 38.53 |  | 10.17 | 10.41 |
| Adaptive Behavior – Functional Pre-Academics | 10.08 | 11.14 |  | 4.89 | 5.15 |
| Adaptive Behavior – Home Living | 43.99 | 46.96 |  | 7.87 | 8.14 |
| Adaptive Behavior – Health and Safety | 45.22 | 47.67 |  | 8.29 | 8.86 |
| Adaptive Behavior – Leisure | 38.69 | 40.85 |  | 5.61 | 6.22 |
| Adaptive Behavior – Motor | 52.92 | 53.99 |  | 5.68 | 6.02 |
| Adaptive Behavior – Self Care | 45.44 | 46.99 |  | 7.10 | 7.66 |
| Adaptive Behavior – Self Direction | 48.71 | 50.99 |  | 7.03 | 7.76 |
| Adaptive Behavior – Social | 61.03 | 62.23 |  | 7.25 | 7.73 |
| Social-Emotional | 138.18 | 142.39 |  | 4.36 | 4.68 |
