# Supplementary material for: Two-year impact of community-based health screening and parenting groups on child development in Zambia: Follow-up to a cluster-randomized controlled trial
Source: PLoS Med. 2018 Apr 24;15(4):e1002555. doi: 10.1371/journal.pmed.1002555 (PMC5915271; doi:10.1371/journal.pmed.1002555)
Supplement: S3 Table — (DOCX) [file pmed.1002555.s005.docx]

|  | **Control** | **Intervention** |
| --- | --- | --- |
| *MICS - Baseline* |  |  |
| Read to child in last 3 days | 0.06 | 0.03 |
| Told stories to child in last 3 days | 0.16 | 0.14 |
| Sang songs to child in last 3 days | 0.73 | 0.79 |
| Took child outside in last 3 days | 0.55 | 0.53 |
| Played with child in last 3 days | 0.81 | 0.82 |
| Named, counted, or drew things with child in last 3 days | 0.07 | 0.07 |
|  |  |  |
| *MICS - Midline* |  |  |
| Read to child in last 3 days | 0.24 | 0.50 |
| Told stories to child in last 3 days | 0.18 | 0.46 |
| Sang songs to child in last 3 days | 0.77 | 0.91 |
| Took child outside in last 3 days | 0.52 | 0.51 |
| Played with child in last 3 days | 0.80 | 0.90 |
| Named, counted, or drew things with child in last 3 days | 0.42 | 0.68 |
|  |  |  |
| *MICS - Reconsent* |  |  |
| Read to child in last 3 days | 0.32 | 0.59 |
| Told stories to child in last 3 days | 0.34 | 0.60 |
| Sang songs to child in last 3 days | 0.81 | 0.91 |
| Took child outside in last 3 days | 0.68 | 0.80 |
| Played with child in last 3 days | 0.96 | 0.97 |
| Named, counted, or drew things with child in last 3 days | 0.60 | 0.79 |
|  |  |  |
| *MICS - Endline* |  |  |
| Read to child in last 3 days | 0.33 | 0.48 |
| Told stories to child in last 3 days | 0.27 | 0.46 |
| Sang songs to child in last 3 days | 0.74 | 0.80 |
| Took child outside in last 3 days | 0.42 | 0.41 |
| Played with child in last 3 days | 0.65 | 0.79 |
| Named, counted, or drew things with child in last 3 days | 0.57 | 0.69 |

|  | **Control** | **Intervention** |
| --- | --- | --- |
| *Food consumed by child in previous 24 hours* |  |  |
| Roller-mean porridge or nshima | 0.90 | 0.94 |
| Breakfast-meal porridge of nshima | 0.11 | 0.09 |
| Rice, pasta, or bread | 0.27 | 0.33 |
| Jiggies or biscuits | 0.32 | 0.31 |
| Pumpkin, carrots, or sweet potatoes | 0.05 | 0.08 |
| Irish potato or cassava | 0.10 | 0.15 |
| Vegetable leaves (e.g., pumpkin, cassava, spinach) | 0.73 | 0.79 |
| Fruits (e.g., mango, orange, banana, guava) | 0.87 | 0.89 |
| Other vegetables (e.g., eggplant, cabbage) | 0.49 | 0.45 |
| Beef or goat | 0.20 | 0.21 |
| Chicken | 0.29 | 0.30 |
| Fish (fresh or dried) | 0.25 | 0.40 |
| Other meat (e.g., field mice) | 0.05 | 0.04 |
| Soya pieces | 0.09 | 0.11 |
| Eggs | 0.52 | 0.57 |
| Groundnuts, beans, lentils, or cowpeas | 0.48 | 0.59 |
| Animal milk, cheese, or yogurt | 0.52 | 0.49 |
| Vegetable oil or butter | 0.86 | 0.92 |
