# Supplementary material for: Two-year impact of community-based health screening and parenting groups on child development in Zambia: Follow-up to a cluster-randomized controlled trial
Source: PLoS Med. 2018 Apr 24;15(4):e1002555. doi: 10.1371/journal.pmed.1002555 (PMC5915271; doi:10.1371/journal.pmed.1002555)
Supplement: S1 Text — (PDF) [file pmed.1002555.s006.pdf]

THE EARLY CHILDHOOD DEVELOPMENT PARTICIPATORY THEATRE BASED CURRICULUM [BUNTU MU BO'ONGO]

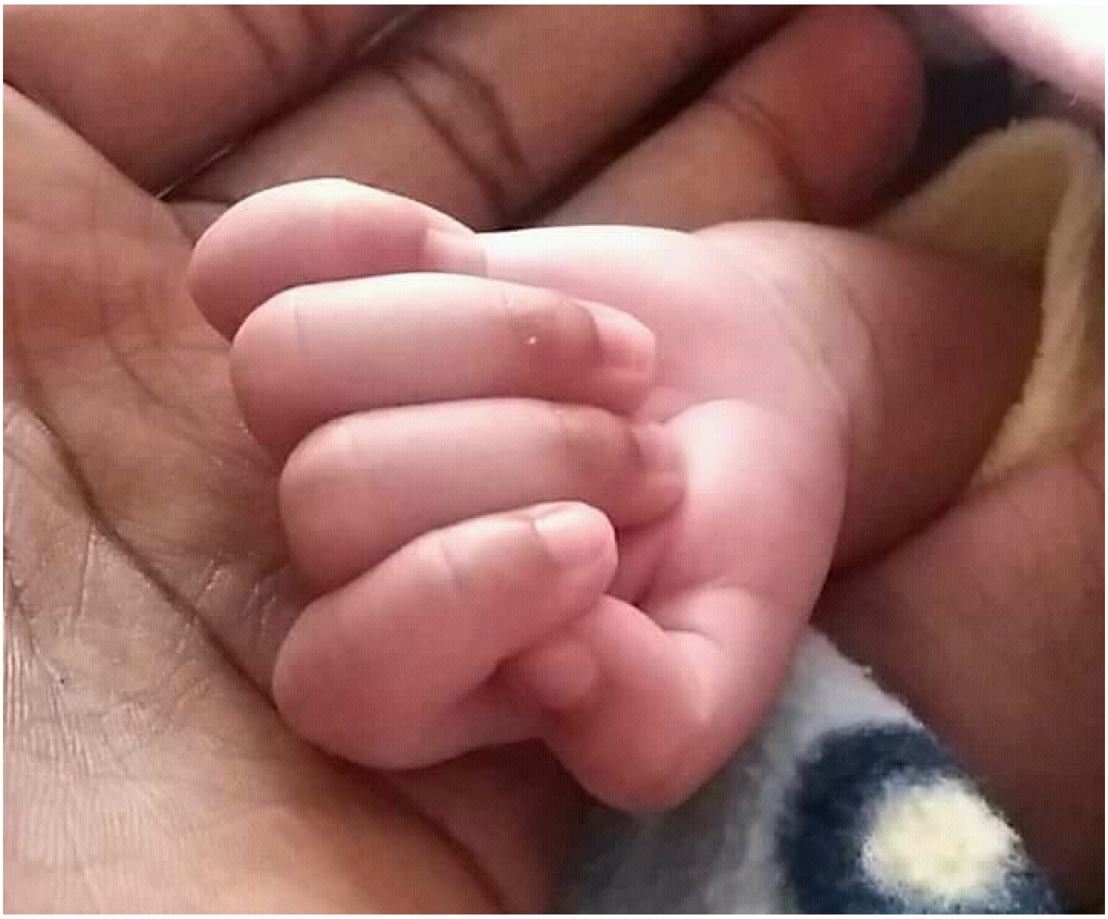

FACILITATOR'S MANUAL

A researcher's guide

[Draft Version 2]

Mwaba Moono Chipili*, Erricah Silukaye, Susan Ng'andu, Loveness Shanabuchinga, Lydia Muleya, Mirror Michelo, Arianna Zanolini and Bowen Banda.

2016/7

In Collaboration with AIR and ZCHARD for the Saving Brains Project

Buntu Mubo'ongo Facilitator's Manual: A researcher's guide.

∗Primary Author: Mwaba Moono Chipili

The main participatory workshop consisted of : all the authors

Cover page picture taken by Mwaba Moono Chipili

***In Collaboration with AIR and ZCHARD for the Saving Brains Project Phase* II**

2016/7

Contents

Foreword

Acknowledgements

Background i

Training with the Buntu Muboongo curriculum ii

Training Requirements iii

Course Outline iv

Facilitator [Requirements] vii

Who is a facilitator? viii

What will this manual help you do? x

Lesson 1: Introduction to early childhood development 1

Lesson 2: Stunting 16

Lesson 3: Cognitive development 30

Lesson 4: Language development 44

Lesson 5: Water, hygiene and sanitation 54

Lesson 6: Physical development 66

Lesson 7: Diverse diets for the growing child 74

Lesson 8: Diverse diets (creating a balanced meal) 84

Lesson 9: Social emotional development (a family affair) 91

Lesson 10: Social emotional development (raising the healthy child) 100

Additional Lessons [optional]

Lesson 11: Into the looking glass firsthand 109

Lesson 12: Sharing Knowledge 117

Additional Material

Activity List 123

Materials Checklist [Not yet completed]

Participant’s manual [Not yet completed]

Background

Zambian children under age 5 face multiple obstacles with respect to their survival and development.[1] Currently, weight and height for age measures show that forty percent of children under age 5 are stunted, 6 percent are wasted, and 15 percent are underweight. [2] It is a critical issue in addition to exposure to infectious diseases, poor sanitation, unhealthy environments and various problems growing children are likely to face. This implies that there is a need for early childhood care and education (ECCE) programs with an integrated and holistic approach[3] as a large body of evidence that shows that such programs – including kindergartens and parenting programs have a powerful effect on improving children's health, cognitive development, and social and emotional development.[4] As such ECD programmes are few in the Zambian context[5] interventions such as the *Improving Early Childhood Development (ECD) in Zambia project* have been embarked on. It is in the second phase of this study [also known as Saving Brains] that this curriculum was developed.

Studies have shown that the level of knowledge a mother has about child growth and a development has an impact on her child's wellbeing.[6] As non interactive methods of information dissemination have proved not to be as effective with regard to interventions focusing on behaviour change[7][8], creative and visual stimuli like pictures, diagrams, colours and physical activities have been adopted as these tend to increase the effectiveness of dialogue in any group regardless of education levels.[9] This curriculum is an attempt to provide support and guidance to help increase caregiver knowledge on child nutrition and stimulation so as to enhance child development in children under the age of five.

The *Buntu Mubo'ongo curriculum* served as a medium through which ECD knowledge on nutrition and stimulation was implemented in the Saving Brains II intervention. This curriculum is based and centred on participatory theatre, available knowledge on ECD as provided by various sources of research as cited and inquiries conducted during this research. It also provides an alternative [not prescriptive] mode of creating dialogue in an intervention.

This curriculum *Buntu Mubo'ongo* prepares the researcher (as a facilitator) as s/he in corroboration with the target participants (or co-learners) to promote child health using local funds of knowledge. This participatory approach gives the target population a sense of ownership which may in turn reduce the sense of alienation that participants may feel in learning environments as an exchange of knowledge occurs.

---

[1]Central Statistical Office (CSO) [Zambia], Ministry of Health (MOH) [Zambia], and ICF International. 2014. *Zambia Demographic and Health Survey 2013-14*. Rockville, Maryland, USA: Central Statistical Office, Ministry of Health, and ICF International.
[2] Central Statistical Office (CSO) [Zambia], Ministry of Health (MOH) [Zambia], and ICF International. 2014. *Zambia Demographic and Health Survey 2013-14*. Rockville, Maryland, USA: Central Statistical Office, Ministry of Health, and ICF International.
[3] Ministry of Health (MoH) [Zambia]. 2012a. *Annual Statistical Bulletin*. Lusaka, Zambia: Ministry of Health.
[4] United Nations Children's Fund (UNICEF; 2014) Study of parental knowledge, attitudes and practices related to early development: Solomon islands. UNICEF
[5] Education for All 2015 National Review Report: Zambia.

[6] Central Statistical Office (CSO) [Zambia], Ministry of Health (MOH) [Zambia], and ICF International. 2014. *Zambia Demographic and Health Survey 2013-14*. Rockville, Maryland, USA: Central Statistical Office, Ministry of Health, and ICF International.
[7] Serpell, R. (1993). *The significance oj schooling: life-journeys in an African society*. Cambridge, UK: Cambridge University Press. (Digitally reprinted in 2010).

[8] Rockers, P., Fink,G. & Levenson, R. (2016, June 2) Tools to Improve Parental Recognition of Developmental Deficits in Children. Paper presented at the Innovations for Behavioural Change in Health: Evidence from Zambia conference organised by American institute for Research (AIR) in collaboration with Millennium Challenge Account Zambia, Lusaka, Zambia.
[9] VSO (n.d) Participatory approaches: A facilitator's guide. Retrieved on 29th July, 2016 from:
http://community.eldis.org/.59c6ec19/VSO_Facilitator_Guide_to_Participatory_Approaches_Principles.pdf

Training with the Buntu Mubo’ongo Curriculum

Materials needed to support learning using this curriculum:

- A researcher’s manual [required] to provide guidance and rationale for activities in the curriculum.
- A participant’s manual [required] for use by participants during learning sessions[10] and for home use.
- An activity list [optional] for use by both the facilitator and participants.
- Materials checklist [optional] for use by facilitator.
- A facilitator trained in facilitation in participatory theatre (this includes theatre of the oppressed techniques and popular theatre)

Curriculum Goals and Constraints

Goals

The overall goals of this curriculum are to:

- Provide a mode of learning that is not only interactive but incorporates indigenous modes of information dissemination in an ECD intervention.
- To act as a guide for the researcher who seeks to use participatory theatre as a means of dissemination in an ECD intervention.

Other goals are to:

- Provide additional information with regard to child nutrition and stimulation.
- Encourage playful interactions between child and caregiver [mother, sibling, father or adult involved in child’s care].
- Use materials from the immediate environment to provide adequate nutrition and stimulation for development.

In this case a participatory approach as well as the use of participatory theatre helps achieve these goals.

Constraints

This curriculum is adapted to specific settings (Choma and Pemba) in the southern province of Zambian. It is therefore, important that the researcher takes note of their context of study before employing some of the activities in this curriculum. Some activities have also been renamed to avoid or reduce issues arising from cultural superstitions; hence the researcher will need to go through original sources to be able to effectively incorporate the activities into their target contexts of study.

[10] Called learning sessions as it is bidirectional in this course, both the facilitator and the participant will acquire and share knowledge.

Training

- Course Outline
- Course needs:
  - Facilitator
  - Facilities
  - Materials Checklist [not yet included]

Course Outline

This curriculum consists of lessons centred on child development stimulation and nutrition. Activities largely include the use of participatory theatre (PT) and cooperative enquiry through prompts (a participatory action research (PAR) method) as a means of gaining information when conducting the learning sessions and workshops.

Lesson One: Introduction to Early Childhood Development

Synopsis: This lesson talks about the community's involvement and role in raising a child. It will serve as an introduction to what will be taught about child development as well as the method to be employed as well as a form of gauging what the participants know about nutrition and stimulation with regard to child development. Three huts and a dice will be moulded to create a homework giving activity.

Activities include: games and exercises from the *arsenal of theatre of the oppressed* and *popular theatre* have be adapted by the primary investigator to suit this context, word games, clay moulding, singing and dancing as well as active story telling.

Lesson Two: Stunting

Synopsis: This lesson seeks to address the issue of nutrition status as indicated by the child's height. Here it is noted that being underweight, discoloration of hair and other basic indicators of malnutrition in children are not the only forms through which it can be noted. Causes of stunting as well as ways of prevention are discussed. Height charts will be guided by the World Health Organisation (WHO) charts for girls and boys by age.

Activities include: games and exercises from the *arsenal of theatre of the oppressed* have be adapted by the primary investigator to suit this context, height chart making exercise, word and dice games, singing and dancing as well as active story telling.

Lesson Three: Cognitive development

Synopsis: this lesson talks about the role of a mother in stimulating good child development using games as well as talking to the child. This is largely encouraging of playful interaction between child and family members as well as discussing and doing activities that enhance child cognitive and language development that can be done with the child even as the caregiver goes about their daily chores.

Activities include: games and exercises from the *arsenal of theatre of the oppressed* have be adapted by the primary investigator to suit this context, word and dice games, butume exercises, book making, singing and dancing as well as active story telling.

Lesson Four: Language Development

Synopsis: this lesson looks at how language development can be stimulated through mother to child talk about past events, songs, rhyming and poetry. Encourages mother to make feeding time an adventure for the child to encourage child to eat as well as promote language development at a critical time (since feeding is one of the child's most important events aside from play).

Activities include: games and exercises from the *arsenal of theatre of the oppressed* have be adapted by the primary investigator to suit this context, feeding exercise game, word and dice games, singing, poetry, rhymes and dancing as well as active story telling.

Lesson Five: Water, sanitation and hygiene

Synopsis: this lesson looks at the dangers of unsanitary habits or practices and their link to stunting. It also discusses alternative methods water purification using readily available cost free methods.

Activities include: games and exercises from the *arsenal of theatre of the oppressed* have be adapted by the primary investigator to suit this context, physical, word and dice games, erranding exercise, book making, singing, poetry, rhymes and dancing as well as active story telling.

Lesson Six: Physical development

Synopsis: this lesson talks about how exercise is integral to the physical wellbeing of the child. It notes that play is how this exercise is carried out, and thus it is important that all children play in one form or the other. At this age, mother serves as the guide for how long this play goes on and what type of play it is.

Activities include: relaxation and trust building exercises (by Augusto Boal though they will be adapted by the primary investigator to suit this context), feeding, exercise, word, physical and dice games, singing and dancing as well as active storytelling and play acting.

Lesson Seven: Nutrition (feeding practices)

Synopsis: this lesson looks at how to prepare a healthy balanced meal for the child, number of meals a day that the child should have, and planning for a meal.

Activities include: games and exercises from the *arsenal of theatre of the oppressed* and *popular theatre* have be adapted by the primary investigator to suit this context, feeding exercise, word, physical and dice games, singing and dancing as well as active storytelling and play acting.

Lesson Eight: Preparing a balanced meal

Synopsis: this lesson act s as a practical for lesson 7. Here a meal is prepared correctly using locally available foods depending on the season. Preparation includes observation on how to make sure food is clean and nutrients are not lost due to overcooking.

Activities included: cooking and feeding

Lesson Nine: Social emotional development *[focus on the mother's well being]*

Synopsis: this lessons looks at the emotional health of the family, especially the mother who in most cases is the primary caregiver, will affect the child's emotional regulation abilities. It asserts that a family has a role to play in the child's social emotional development.

Activities include: games and exercises from the *arsenal of theatre of the oppressed* have be adapted by the primary investigator to suit this context, word, physical and dice games, singing and dancing as well as active storytelling and play acting.

Lesson Ten: Social emotional development [*focus on the child's well being***]**

Synopsis: this lesson looks at how a well regulated child, that is, one whose environment has been made conducive for healthy growth, is able to develop meaningful and healthy relationships and interactions.

Activities include: games and exercises from the *arsenal of theatre of the oppressed* and *popular theatre* have be adapted by the primary investigator to suit this context, word, physical and dice games, singing and dancing as well as active storytelling and play acting.

Lesson Eleven: Recap on lessons learnt

Synopsis: this lesson serves as an evaluation the curriculum according to the participant's views. Here lessons are discussed and favourite activities are repeated.

Activities include: Any activities from previous lessons as selected by the participants.

Lesson Twelve: Performance [Optional]

Synopsis: This curriculum is embedded in participatory theatre, hence the entire process is treated as if the participants are preparing for a big show. In this case, the entire group prepares performances and a date for the show is set. All the activities, especially the arsenal of theatre of the oppressed and popular theatre are meant to improve group cohesion and provide the participants with skills as actors in the final show.

Facilitator [Requirements]

- Facilitators need to have an understanding of a comprehensive participatory theatre (PT) and it's aesthetics before they are allowed to facilitate these learning sessions.

- If working as part of a participatory workshop, refresher trainings for facilitators should be organized at least every 6 weeks of optimal delivery of information to occur, especially if they have had no prior experience with this method. The first workshop should last at least a week; then follow-ups can occur.

  Note: These sessions do not need to be as intensive as the first ones and serve as a means of monitoring if methods of facilitation taught are what is being done; unless the trainer observes the need. However, it is expected and of highest importance that all facilitators keep themselves updated about technical issues covered in participatory theatre as part of their development.

- Each session requires two facilitators. The team of two facilitators per group should clarify who is facilitating which part of the activities/discussions beforehand. It is fine if one of the facilitators takes a lead role in conducting the activities while the other one assists (e.g. by note taking, demonstrations of certain activities, distributing learning manuals and ensuring materials needed for that session are available) or if both share the activities and change roles throughout the station. It is crucial that the two facilitators agree and rehearse in advance who does what. Both facilitators are responsible for what happens during the learning session.

- The facilitators act as a well coordinated and strong team in order to optimally support the participants and serve as role models for a constructive way of communication. During each session, the facilitators are to guide the participants through the lesson, which consists of different activities and materials to promote a discussion and interaction among participants and the facilitators related to the respective key messages.

  Note: Each facilitator has a different personality with strengths and weaknesses. It is recommendable to be aware of your own strengths when taking over the role of a facilitator in this curriculum. Often it is particularly easy for two facilitators to successfully cooperate, if their strengths complement each other.

Facilities

- This curriculum can be taught in any safe environment [either outdoors or indoors]. It has no structural requirements. Location is dependent on the researcher, facilitator or participants

Who is a facilitator?

In this course anyone who guides the learning process of this curriculum is a facilitator.[11] This facilitator will help participants learn the skills presented in the course *The Early Childhood Development Participatory Theatre Based Curriculum [Buntu Mu Bo'ongo].* Participants in this course will learn the skills to needed to raise healthy, capable, and happy children; as well as serve as information base for the facilitator on local aesthetics that can be incorporated in this curriculum so that it may better serve them.

In this curriculum, you will demonstrate what a mother [or primary/secondary caregiver] needs to do, lead discussions, help participants practise skills and give feedback to them as well as receive feedback from them. You will not offer any medical help unless you are qualified to do so. You will give participants any required help they may need to successfully complete the course and learn the skills that will help them improve the development of their children in the community.

The **Participants' manual and the activity checklist** structure the process of learning the information the caregiver will need. Your task is to facilitate their use of these materials and a ratio of two facilitator to 12 or 20 participants is recommended for facilitators to give enough attention to participants in the course to learn information and skills. Two facilitators work as a team with a group of participants.

What do you do, as a facilitator?

Your major responsibilities of the facilitators are to:[12]

- encourage participants to talk and share their own knowledge and to enjoy this;
- encourage participants to reflect their own behaviour and actions;
- provide participants with correct information during discussion segments ONLY if the group does not know the correct answer to a question;
- give options where to get further information and encourage participants to access (family-friendly) health services;
- Give options for behaviour change: widening of choices for protective behaviour taking into consideration the needs of the mother and her child.

Note: *Facilitators are the heart of any good learning session as the success of lessons depends on good facilitation. A facilitator needs to know and practice facilitation skills such as creating a good learning environment, asking the right questions and practice probing and bouncing back of questions during facilitation. A good facilitator should avoid lecturing! Please regularly practice self-reflection based on the recommended participatory facilitation methods.*[13]

[11] The terms researcher and facilitator will be interchangeable in this manual.

[12] VSO (n.d) Participatory approaches: a facilitator's guide. Retrieved from: http://community.eldis.org/.59c6ec19/VSO_Facilitator_Guide_to_Participatory_Approaches_Principles.pdf

[13] Refer to VSO (n.d.) participatory approaches: a facilitator's guide; and Boal, A. (2005) games for actors and non-actors.

Always Remember to:

- ***Read through the lessons a day or two before you share it with the participants.***
- ***take note of the materials you need to access before the lesson occurs.***
- ***Read through the information bank so that you can summarise the content during the lesson. You don't need to read it word for word.***

For your eyes: some colour codes in the lessons

Purple: All notes or guidelines or instructions are directed at you.

Light Blue: Notes in light blue are directed at both you and the participant.

Dark blue: these notes can be shared if you feel the need to.

What will this manual help you to do?

This ***manual*** will guide you through the learning sessions in this curriculum. They indicate how to use the ***lead*** each session. They also describe the ***objectives*** of the lessons, rationale for activities used and a list the ***materials needed*** for the session. They guide you through the ***process*** of a session with the participants.

To prepare yourself for a session:

- Read the ***facilitator's manual***.
- Meet with your co-facilitator to identify what the session requires and who will prepare for which activities.
- Gather and organize the supplies and other materials needed for the session.
- Practise activities, demonstrations, and other activities which are new for you. Especially '***the play of sorts'*** activities.
- Refer to the recommended readings noted in the footnotes for the further clarification, if necessary.
- Identify possible questions participants may ask, and practise how you will answer them.

Lesson 1: A Re-introduction to Early Childhood and Development

It takes a village to raise a child

Objectives:

By the end of this session participants should:

- Reduce anxiety and build trust
- Reaffirm hopes for child's growth
- Learn what early child development is
- Discuss the importance of maternal involvement in ECD
- Know why it is important to integrate ECD learning with nutrition
- Reaffirm why they are here and developmental benefits

Materials Needed:

- Drum [old pots or buckets and sticks can be used in the absence of a drum], clay, grass or twigs, old juice bottle [preferably a plastic coke bottle], knife or scissors, plastic wrappers [blue, green and brown], old plank [a plastic or metal sheet can also be used], ball [or soft cloth] and lots of care ☺.

NOTE: *Always keep a bucket of clean water, a cup, soap or ash, a clean piece of cotton chitenge and dish for hand washing.*

Introduction

Facilitator:

Always remember to begin a session by:

- Greeting the participants and their children.
- Introduce yourself and briefly talk about what the meeting is about.
- Encouraging co-learners to sit in a circle, imitative of a village meeting. If possible the facilitator can have a drum[1] (old pots or buckets can serve as alternatives) present to begin the session.
- Thanking participants for attending this meeting and telling them you are happy to be meeting with them.

[1] A drum is an aesthetic of popular theatre as a part of this context's oral culture, I felt it would be an effective means for signalling to the participants that the session has began as well as getting everyone involved through sound and movement.

NOTE: *Always be welcoming, smile, be friendly and open (approachable). It is important to be welcoming so that participants can open up and feel free to express themselves, and also be able to approach you when they need help.*

Relaxation and trust building exercises

Facilitator:

- Ask all to stand, maintaining a circle, and do a stretch exercise.[2] For instance, stretch arms upwards, sideways, back to front, touch your toes, bend your knees, jump and try to touch the sky (reach for the heavens) and move head and neck.
- Encourage mothers to encourage children to stretch as well.

Always: pair these with breathing exercises (make funny faces and sounds with each stretch).

NOTE*: We are doing this to relieve tension, to relax because a mother is supposed to be stress free (be in a good mood, balanced mood/ state) so that she can pay better attention to her child.*

Sing-Along Time

Facilitator, say:

- "Does anyone know a song that talks about raising a healthy child?"
- If so, can *[insert name of person who assents]* teach it to the rest us? If not, let's make one up?

  Always encourage use of local songs.

[2] This exercise was inspired by notes in Boal, A. (2005) Games for actors and non-actors and Anderson, J., Michel, J & Silverberg, J (2001) Ready for action: A popular theatre/popular education manual, encouraging a warm up before a lesson begins so as ready the learners for learning.

Circle of knots exercise[3a]

Facilitator:

- Ask mothers to make a big circle (do not hold hands): stretch out arms until the fingertips are almost touching.
- Then tell mothers to make a small circle or a huddle until no space is left.
- Ask mothers to chant the ritual or group name (e.g. say: wise mothers! Or clap hands together or both) when circle is small.[4]
- Then make a big circle once more.

Q&A

Q: Why are we doing this exercise?

A: We are doing it to relieve tension, to relax because a mother is supposed to be stress free (be in a good balanced mood) so that she can pay better attention to her child. Don't worry, be happy!

NOTE: *This activity helps boost the morale and reminds learners that these sessions have a positive goal. You can also use this exercise any time the morale is low.*

Getting to Know Each Other

The Name Game[b]

Please note: This game is most effective for a group that does not know each other very well or a new group However, you still do it even if you already know the co-participant's names. This game helps co learn each other's names in an interesting and active manner. It is important for the facilitator to theme the game. For example, we are going to say our names and act out what we like to do. Or act out what we wish for our children.

[3] Only the first part of this activity, the elastic circle is used.

[4] It is important to allow participants to create a group name or ritual (activity or signal) with which they can identify with This should be done during the lesson or meeting. This should not be confused with the ritual activity (sound, gesture) in Boal, A (2005) Games for actors and non-actors..

Facilitator, say:

- Here, one of us will say their name together with an action. For instance, I can say, 'my name is Luyando' and because I like birds, 'I will imitate a bird by fluttering my wings' (spread out arms and pretend to fly) as I say my name. [So if my name is Luyando and I like to or wish to do something (e.g. cooking) or be able to be someone in future (e.g. a kind mother). I say my name and choose an action that best describes my likes or wishes, then the co-participant either lifts or points or refers to child and encourages child to say their own name (child may imitate mother's action)].
- The group will then repeat the mother's and or child's name with action. [This helps child develop sense of self (I am me) and others (they are and we are) other than his/her name and those around].
- Now let us all seat down. [In the absence of chairs use vitenges or mats.] Don't forget to maintain the circle.

Variations: game can change, over time in that facilitator can include vocal sounds, clapping, song during which co-participant shouts name, then the child's name and others repeat afterwards. Here other participants can sing back the name and do the action. Another variation could be that participant says their name, or just says out what they wish or hope for themselves and child.

This activity allows mothers to actually think about what they truly want for their child and themselves. It also helps develop concentration, memorization, creativity and motion skills as well as character development (an imagination skill).

Inquiry Questions:

How are you feeling? What do you think about the exercise? Did you understand each other's actions? What did you think about as you did the action? Did it help you think about what they really want for yourself and your child? Do you know why they are here? Why are we meeting? Or what made you come or want to be part of this meeting? Have you had any lessons talking about child health in the past? If yes, what information do you remember?

A Throw Down Memory Lane

Materials: a home-made ball [you can tie a soft cloth into a ball if this is not available]

Facilitator:

- Briefly introduce what the groups are about.
- Then add: 'We've all come to learn from each other. Feel free to ask, correct, teach, etc.'

In this case we are here to talk about what early childhood is and what role mothers play in a child's growth.

A Tale [to be told by facilitator or co-participant] [5cdef]

Once upon a time, in a not so far away land a story is told about a chief called Juulu. Chief Juulu was a very wise old man. One day as he was learning from the stars he realized in a vision that children are the key to the future. So he searched far and wide for the wisest people to help him understand this vision. Of all these people, he found out that mothers were the wisest and most likely to know what would be best for their children. Soon, he sent his emissaries to learn from these mothers and share their knowledge in groups that would allow for the sharing of this "wisdom" so that it could be used by all, as it takes a village to raise a child. We welcome you to the meetings focused on how to improve our children's development.

Then say:

- Now, we will take this time to use the ball of remembrance[6g].
- Here, we will say what we know or remember about child development. For instance, facilitator can use these questions as guides: what do you know about early childhood development? Or what do you think early childhood development means? etc.

[5] Banda (2002) argues that storytelling (a key component of African oral tradition) is a local fund of knowledge through which learning can occur. In this case it also serves as an aesthetic of popular theatre along with songs, fables and other oral forms of communication that are indigenous to African tradition (Kabaso, 2013; Manukonda, 2013;Mlama, 1991) including Zambia.

[6] This activity is named for the passing technique used in acting during character development or learning script (Barton, 2009). In this case, I felt it would work well in gauging how much the participants knew on child development.

- After, I ask a specific question, I will pass the ball to one of you.
- The person holding the ball has to give an answer; then pass the ball to next person who also has to answer.
- We will do this while sitting [or standing, depending on what position we feel like being in] and whoever has the ball says something about child development.

Note: This game can be accompanied by a song and person with the ball when song ends says something.

INFORMATION BANK: EARLY CHILDHOOD DEVELOPMENT (ECD)

Early childhood development [h]

Early childhood is a critical stage of development (from zero to three years) that forms the foundation for the child's future well-being and learning. It is in this phase that children learn the most, and things that they experience in early childhood will shape who they become. This does not mean that development cannot be stimulated after the age of three.

What are the main areas of development? [i]

The areas of development are– cognitive (thinking ability of child), language (talking), physical or motor (walking, hand movements, and other body movements), and social/emotional (talking, playing with others) – which all contribute to the long-term well-being of the child. It is therefore important to take steps to ensure the growth of child development.

Why is it Important? [j]

These early years of life are a window of opportunity to lay a strong foundation for a child's life. Proper health, nutrition, and early stimulation play a critical role for brain development and child well-being. Around the world, poor children under 5 lag behind their more advantaged peers in physical, language, cognitive, and socio-emotional development. Without access to quality ECD, poor children often fall behind their more advantaged peers before they even begin school. As they get older, the gaps widen: they are likely to perform poorly in school, earn less as adults, and engage in risky social behaviours.

---

[7] In this segment, not much information was added as the target participants had already received prior information on ECD. If need be, the facilitator is free to add more detail as long as it is scientifically sound, or reduce the content. This lesson, in this context served more as an introduction to the theatre component.

Supporting early childhood development improves equity improving the health, nutrition, and education outcomes of children.

Children who participate in quality ECD programs **are more ready to learn** when they begin school and are less likely to repeat grades or drop-out of school, which reduces the overall costs of the education system. When they get older, they are more likely to earn more and less likely to engage in crime.

Why should you be involved?[k]

It has been noted that learning is enhanced when parents/family and the early childhood setting work together. By being involved in your children's early learning, parents can improve their children's motivation to learn and thereby enhance their growth potential.

Luyando and Luano: A Story Stem Activity[8l]

Facilitator:

- Here you create a fictional character 'Luyando or Luano' and make a bit of a back story or beginning. For instance: "Luyando was a happy little, who lived in Pemba. Every morning her mother would……."
- Then, each person will take turns saying something or adding to this story. For instance, saying something that led to good development of Luyando or the poor development of Luano.
- Then discuss the good or bad aspects of this story and allow for corrections where mistakes were made (misinformation).

Do not be judgemental. Your role as a facilitator is to guide.

Remember*: Use prompts especially if co-participants cannot decide which attributes are healthy or unhealthy. For instance, you could say, 'Do you think that action was good for Luyando or Luano? Or what about that action made you think that it was a healthy or unhealthy choice?*

NOTE: *This story can serve as a basis for discussion on what is known as good nutritional and care giving practice in your context of study. It allows you as a researcher to take note of the basic information that the participants have on a given topic.*

[8] This is inspired by the story stem assessment. In this instance, I used it as a source for a verbal representation of what the participants know about what practices lead to healthy or healthy development in a child.

The Mother Hen Activity [9m]

Facilitator:

- Ask all to stand in two lines (queues) then face each other.
- Then tell them that the person they are facing is now their mother in this exercise. This mother must take care of her "young child" and make sure no harm comes to them, for instance bumping into someone, or a stone or tree or wall. The chick is supposed to follow the mother's voice and trust that the mother hen will keep her safe.
- Ask the mother hens, to make chicken sounds (clucking) and approach their chicks (who have to close their eyes), then begin to walk. Mother must make sure that their child is following, able to hear her clearly. The chick must follow the sounds of their mother hen.
- The mother hen make three sounds: a quiet, calm noise for the child to follow, one loud noise to warn of danger so that the chick can stop when faced with "danger"(please note that is no actual danger, do not cause harm or encourage it) and then a humming sound to act as a pretend feeding break.
- The goal is for the mother hen to guide, and protect their chicks without touching them with her hands. Do not force, guide.
- Once this is done (duration, 5-7 minutes), the facilitator can ask the following questions per pair:

Questions

How did you (the 'chick') feel as you were being led? How did you (the 'mother hen') feel as you led your chick? What did you fear? At what point where you really worried? What did you think or do, in that instance?

- And then the one who was the mother hen can now play the role of the young chick and the one who was the chick at first can now become the mother hen.
- Repeat the questions after the switch and exercise is done.

[9] This activity is originally known as Noises in Boal's Games for actors and Non-actors, second edition. However, I renamed it, after observing a hen and its chicks, and added to the rules to suit the goals of this course. In this case this activity proved very effective in explaining what a mother's role is in her child's life. The mothers and community health workers also felt it was a very relatable concept.

> ***NOTE***: *You as the facilitator act as a guide through this process. You will decide when to start and when it's feeding time. You will also explain the game to other participants as it begins. Perceived danger however will be determined by the" mother hen" who will lead the chick. Only the chick closes its eyes, the mother hen needs her eyes to remain open so that she can see where they (mother and chick) are going. If children are present the mother hen also takes responsibility for the children as well. She must encourage them to take part in the movements as well.*
>
> **Additional questions [ask after this exercise is done]**: Did you learn anything from this exercise? Is this similar to the relationship between a mother and her child? If yes, why? If no, why? What is a parent's role in the child's development?

Discussion: Guide, Protect and Feed[10]n

Facilitator:

- *treat this discussion as a continuation of the mother hen activity. Feel free to refer to this activity, especially if participants appear at a loss for answers.*

Question: Is Nutrition important?

Response: Yes or No [ask participants to give a reason why regardless of the response]

> ***NOTE***: *In our case, our argument is for 'Yes'. However, give the participants a chance to air out their views before making this case.*

Why is it important?

Nutrition should be considered when feeding a child. This is because, when a child is not well fed (diverse diets and balanced diets) then the child will not grow properly. **Development is therefore affected if a child is malnourished or undernourished.**

Inadequate nutrition before and after birth (in the first years of life) can seriously interfere with brain development and lead to neurological and behavioural disorders such as learning disabilities and mental retardation, stunting and many other negative defects.

---

[10] This discussion is guided by the mother hen activity, and acts as a response to the question on a parent's role. Using this activity to drive the information home is easy as it acts as a visual and practical example for the mother.

The malnourished child's immune system may become weak, making the child more susceptible to illness. The child's physical growth is also affected.

Question: Can a crop (e.g. maize or sweet potatoes) grow properly without the sunlight, water and fertilizer?

Response: No [in this context this response was unanimous as agrarian life is the norm]

Add: and this is the same case with children.

Additional Question: What are some of the locally available foods that are good for the child's healthy growth?

Always Remember: *Good nutrition and adequate psychosocial stimulation (through playful interactions between mother and child and child's peers and other adults) are necessary for a child to grow well. So feed child well, talk to them, and so on.*

Creating the Village of Dreams[11]

Facilitator: create a checklist before you begin to ensure that all the required materials are available.

Materials: 1 medium to large cardboard box or plank (any safe clean item that can serve as a base), liquid glue (or tree glue), a pair of scissors (or knife), **clay[12]**, twigs / and dry grass, colourful paper (small pieces to paste on door), ruler and an old plastic bottle (preferably one with a pointed top e.g. a Mazoe or coca cola bottle).

NOTE*: If you do not know how to mould clay, introduce someone either from within the group or outside who can act as the guide for this activity. All must participate. The village doesn't need to be perfect, only that it must be a group effort, so as to allow for social interaction.*

During this activity, the mother must also help her child or children make a clay dice and she must guide them through this activity. It is important that the mother talks to and with the child, smiles a lot and praises the child's efforts as well as encourages that child throughout the process. This serves as an opportunity to encourage maternal guidance (scaffolding) of child as they (mother and child) create something.

[11] In the Zambian clay moulding is something almost all adults did during childhood. I created this activity after observing children in my neighbourhood, Kalingalinga; play with clay outside my gate. This idea was further brought to by studies such as the Panga Munthu test (Kathuria and Serpell, 1999) where moulding of wet clay into a person is used as a mode of assessment. During the curriculum development, mothers and child development agents (CDAs) also agreed that clay moulding and hence the making of huts (and other clay figures) is a common trait in this context as well. The CHWs went on to inform the researcher, that the art of hut making using clay had evolved and children where now using old bottles instead of making the huts block by block as was done in the past by the researcher and also as observed in Kalingalinga

[12] Clay dough can be used but it is important to allow participants to use materials that are readily available in their context. In this case, it was actual clay.

Some specifications: there must be three huts: 1) hut 1_ blue door or plastic, is the hut of wishes, hopes dreams the mother has for her child; 2) hut 2_ brown door or plastic, is the hut of activities that mother will do with the child at home to stimulate good development, even with a busy schedule, and 3) hut 3_ green door or plastic, is the hut where the mother is asked to do something for herself that will help her be more relaxed and healthy. For instance, does she have any one who can help her take care of her children or help with the house chores? Does she have someone she can talk to? In this scenario, the facilitator can encourage the mother to share some of her problems (one or two) and the group can provide emotional support (especially if she does not have any close friends) or help her reach a solution using the resources available to her.

Demo from Simbulo, Kasiya: village of dreams and two die.

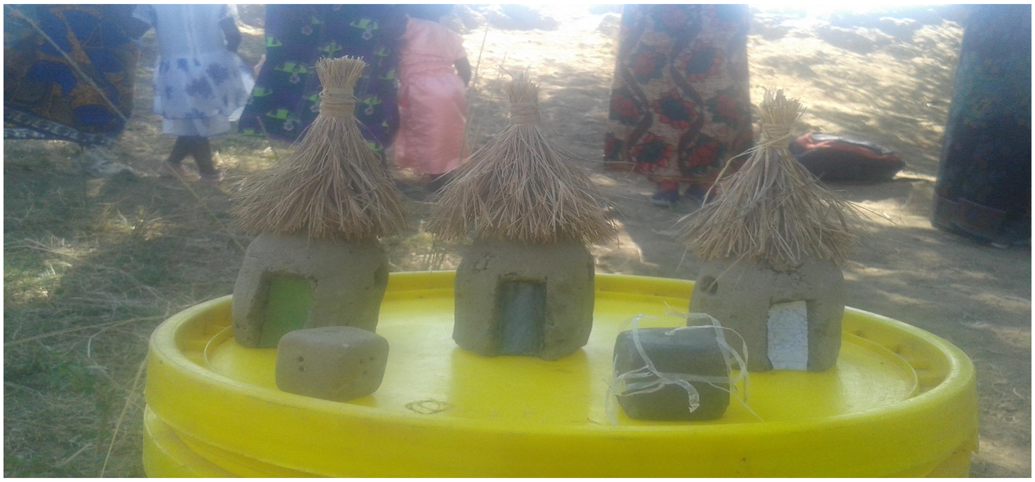

Picture taken by Mwaba Moono Chipili

Instructions [from activity guide[13] or facilitator]:

Making the huts

- Sand the plank or reinforce card boards.
- Wet the clay (if dry although it is usually wet), then mix it with your bare hands.
- Cut the plastic bottle in half with a knife, and then push the clay into the top half while it is upside down. Make sure that it fills this part completely. Feel free to press the clay with your palms or fist.

[13] This refers to person who will guide this activity in case the facilitator does not know how to make these huts. In a context where this activity is not common you can replace it with one that is and still maintain the representations.

- Then turn the bottle down (so that the small part faces up) and hit the top, while the clay is still wet, until the moulded clay comes out, onto the sanded plank or card board or plastic lid (flat surface required).
- Next, cut a few twigs, and stick them to the pointed part of the mould until no clay be seen from the top and a roof is made. Then tie a piece of sack string or tree bark at the point where the twigs meet. [You can use water to make the twigs stick better, or sew a roof using reeds and cotton and simply place it on top instead.]
- Afterwards, cut a strip of coloured paper to stick as a door.
- Then leave to dry.
- Repeat this process until you have three huts.
- Next create the dice

Making the dice:

- Use remaining clay, to mould a square block.
- Then stick, tiny pebbles on each side [starting from one to three only].
- Leave to dry
- Once done, wash hands and clean up working stations.

> ***NOTE****: Once, the huts and dice are dry a game will be attempted. Here, the mother will throw the dice. Then depending on which point the dice falls (1, 2 or 3) that is the activity (her homework) she will do that week. An activity list is provided for huts 1 and 2, while hut 3 activities are subject to the mother's wishes for herself.*
>
> *This game is aimed at helping the mother think of her child and herself in a positive light, as well as provides activities that a mother can do with the child. It also encourages a mother to talk about what milestones the child may have attained depending on their ability to do certain tasks. Discussions can also be held on what techniques the mother used to encourage her child in that task and the difficulties faced when doing the activity (in the next session). This also creates a form of emotional support group/ session for the mother.*

General Homework:

Facilitator, say:

- **Talk to your child about the day to day activities as you do them. For instance, you can talk about sweeping, why you are doing it, whether he or she would like to learn how to do it?**

Sharing Knowledge: 15 minutes of fame

Note: this activity could be optional as it depends on the group's willingness to do so. However, it is a good tool for informing the community on what was done during the intervention or programme. It also gives the participants a sense of purpose.

Facilitator, say:

- Now, we have learnt, recalled and even applied some information relating to early child development, it is our duty to share this information just as the chief did. Therefore, let us take this time to plan a final performance to be shared with the community.

Good day exercise [the final act]º

- Greet or hug each other and tell each other something nice for the day. Here, each mother hugs another mother and says positive things about the other. For instance, when Jane greets Mary, she will say, 'Hi my name is Mary and I am very kind.'
- Then May will return the favour, by saying her name is Jane and…....

Note: this allows mothers to leave group with positive feelings about themselves and each other.

NOTE TO FACILITATOR

Things to consider during the sharing knowledge segment:

Personal goals: will be obtained from the name exercise, wishes, dreams for self and child and other discussions that focus on the mothers' personal hopes.

Group goal: should be discussed in the sharing knowledge segment

Planning for the final Performance:

[Guidelines follow the popular theatre seven step process of performance planning]

Why: discuss personal and group goals?

Who: choose who this performance will be for, e.g. community, friends and family, etc.

When: what will you do? What is the time frame?

Where: will the performance be outdoors or indoors, under a community tree, at someone's home, etc.?

Purpose: what do you want to learn or recall from your performance? What do you want others (your audience) to learn?

What for: what will your play talk about?

How: how will you achieve your group goals in your play and/or presentation?

[14] Refer to Michael, J., Michel, J. & Silverberg, J. (2001) Ready for action: A popular theatre/popular education manual, page 30 for further guidelines on how to plan a performance using participatory theatre.

References

[a] Boal, A (2005) Games for actors and non-actors, second edition. Taylor and Francis online publication. [Only the first part of this activity was used as the second part was deemed too complex at this stage.]

[b] Michael, J., Michel, J. & Silverberg, J. (2001) Ready for action: A popular theatre/popular education manual. Retrieved from: http://wwwwpirg.org

[c] Banda, D (2008) Education for All (EFA) and the "African Indigenous Knowledge Systems (AIKS)": The case of the Chewa people of Zambia. *International Journal of Educational Development*. Retrieved from https://www.researchgate.net/publication/37245744

[d] Kabaso, S (2013) *Theatre for Development in Zambia*. Zambia: Kabsy Digital Media.

[e] Manukonda, R. (2013) Theatre- Communication that captivates and enchants. *Global Media Journal-Indian Edition*, 4(2), 1-11

[f] Mlama,P(1991).*Culture and development : the popular theatre approach in Africa*.Uppsala,Sweden: Nordiska Afrikainstitutet.

[g] Barton, R. (2009) Acting: Onstage and Off, Fifth edition. Wadsworth, Cengage learning: Boston.

[h] United Children's Fund (2006) Programming experiences in early childhood development, first edition. UNICEF

[i] UNICEF (n.d) Early childhood development. Retrieved from: http://www.unicef.org/dprk/ecd.pdf

[j] The World Bank (2010) FAQs: early childhood development. Retrieved from: http://siteresources.worldbank.org/EXTAFRREGTOPEDUCATION/Resources/444707-1291071725351/ECCD-factsheet-final.pdf

[k] French, G. (2007). *Children's Early Learning and Development,* Research paper. Dublin: National Council for Curriculum and Assessment. Retrieved from: http://www.ncca.ie/early-learning.

[l] Lees, G. (2016) The Story Stem Assessment. Retrieved from: http://www.lastehaigla.ee/public/vaimse_tervise_keskus/6.10.16_Gabrielle_Lees_lecture.pdf

[m] Boal, A (2005) Games for actors and non-actors, second edition. Taylor and Francis online publication.

[n] Communications Support for Health (n.d) CSH Nutrition promoter guide 1st 1000 most critical days: the best start to life. CSH & USAID: Zambia.

[o] Boal, A (2005). Games for actors and non-actors, second edition. Taylor & Francis online publication.

Lesson 2: Stunting_ A Crisis

Raising a child is like building a house

Objectives:

By the end of this session participants should:

- know what stunting is
- be able differentiate between stunting, dwarfism and wasting
- know the risk factors that cause stunting
- be able to tell if their child is stunted or not
- be able to know the steps to take if child is stunted

Materials Needed:

- Drum [old pots or buckets and sticks can be used in the absence of a drum], manila paper (or old carton boxes), clear cello tape, 1 meter board ruler (or measuring tape), coloured markers (one or more), a pair of scissors, ball stick, a clay village (village of dreams), activity list and a dice.

> ***Remember:*** *Always keep a bucket of clean water, a cup, soap or ash, a clean piece of cotton chitenge and dish for hand washing.*

Introduction

Facilitator:

Always remember to begin a session by:

- Greeting the participants and their children.
- Introduce yourself and briefly talk about what the meeting is about.

- Encouraging co-learners to sit in a circle, imitative of a village meeting. If possible the facilitator can have a drum[1] (old pots or buckets can serve as alternatives) present to begin the session.
- Thanking participants for attending this meeting and telling them you are happy to be meeting with them.

NOTE: *Always be welcoming, smile, be friendly and open (approachable). It is important to be welcoming so that participants can open up and feel free to express themselves, and also be able to approach you when they need help.*

Relaxation and trust building exercises

Facilitator:

- Ask all to stand, maintaining a circle, and do a stretch exercise.[2] For instance, stretch arms upwards, sideways, back to front, touch your toes, bend your knees, jump and try to touch the sky (reach for the heavens) and move head and neck.
- Encourage mothers to encourage children to stretch as well.

Always: pair these with breathing exercises (make funny faces and sounds with each stretch).

Facial expressions[3] help teach the child how to express emotions.[a]

Remember: *We are doing this to relieve tension and relax so that the mother is to be stress free (in a good, balanced mood/ state) so that she can pay better attention to her child.*

Sing-Along Time

Facilitator, say:

- "Does anyone know a song that talks about the effect of a mother's care on the child? For example, an action song like iluyando lupati (talks about love).

---

[1] Refer to footnote '1' in lesson 1

[2] This exercise was inspired by notes in Boal, A. (2005) Games for actors and non-actors and Anderson, J., Michel, J & Silverberg, J (2001) Ready for action: A popular theatre/popular education manual, encouraging a warm up before a lesson begins so as ready the learners for learning.

[3] Facial expressions are an essential survival emotional skill, especially during the first years of life, through which the child is able to elicit the caregiver's help and attention. Children also have a predisposition to attend and respond to facial expressions from their primary caregivers, hence the mother's face serves as the child's learning interface in this case.

- If so, can *[insert name of person who assents]* teach it to the rest us? If not, let's make one up?

Always encourage use of local songs.

Circle of knots exercise[4b]

Facilitator:

- Ask mothers to make a big circle (do not hold hands): stretch out arms until the fingertips are almost touching.
- Then tell mothers to make a small circle or a huddle until no space is left.
- Ask mothers to chant the ritual or group name (e.g. say: wise mothers! Or clap hands together or both) when circle is small.[5]
- Then make a big circle once more.

Q&A

Q: Why are we doing this exercise?
A: We are doing it to relieve tension, to relax because a mother is supposed to be stress free (be in a good balanced mood) so that she can pay better attention to her child. Don't worry, be happy!

NOTE: *This activity helps boost the morale and reminds learners that these sessions have a positive goal. You can also use this exercise any time the morale is low or when learners seem tired or attention seems to be wandering to bring their focus back to the lesson at hand.*

Getting to Know Each Other

The Name Game[c]

Please note: This game is most effective for a group that does not know each other very well or a new group although; you can still do it even if you already know the co-participant's names. This game helps persons each other's names in an interesting and active manner. It is important for the facilitator to theme the game. For example, we are going to say our names and act out what we like to do. Or act out what we wish for our children.

[4] Only the first part of this activity, the elastic circle is used.

[5] It is important to allow participants to create a group name or ritual (activity or signal) with which they can identify with This should be done during the lesson or meeting. This should not be confused with the ritual activity (sound, gesture) in Boal, A (2005) Games for actors and non-actors..

Facilitator, say:

- Here, one of us will say their name together with an action. For instance, I can say, ‘my name is Luyando’ and because I like birds, ‘I will imitate a bird by fluttering my wings’ (spread out arms and pretend to fly) as I say my name. [So if my name is Luyando and I like to or wish to do something (e.g. cooking) or be able to be someone in future (e.g. a kind mother). I say my name and choose an action that best describes my likes or wishes, then the co-participant either lifts or points or refers to child and encourages child to say their own name (child may imitate mother’s action)].
- The group will then repeat the mother’s and or child’s name with action. [This helps child develop sense of self (I am me) and others (they are and we are) other than his/her name and those around].
- Now let us all seat down. [In the absence of chairs use vitenges or mats.] Don’t forget to maintain the circle.

Variations: game can change, over time in that facilitator can include vocal sounds, clapping, song during which co-participant shouts name, then the child’s name and others repeat afterwards. Here other participants can sing back the name and do the action. Another variation could be that participant says their name, or just says out what they wish or hope for themselves and child.

This activity allows mothers to actually think about what they truly want for their child and themselves. It helps develop concentration, memorization, creativity and motion skills as well as character development (an imagination skill). It’s also fun to do!

Homework Review: Talking to your child

Facilitator, ask:

- Did any of us, do the homework we said we would do in the previous lesson?
- If yes, how did we feel about the exercise? Was it helpful? Was it easy or difficult to?
- If no, why? [Do not be judgemental; instead take this time to encourage the mother together with the team to do it next time. Also give other mothers a chance to share how they managed to do the task.]

> **NOTE**: *although this activity will have been given by you. Remember that your role is to facilitate. Here, the idea is to provide a platform whereby mothers can encourage each other to perform activities that stimulate positive child development. If a mother did not do anything with the child, you will not only encourage her to do an activity with the child but also take this opportunity to allow mothers to suggest activities that she can do to stimulate her child's development as they work (do household chores, e.g. talk to child about the work, ask child "to help" with chores, for instance, hold this cup for me).*

The Life of Luano: A play of sorts

Ball-pass activity[d6]

Materials: a home-made ball [or soft cloth]

Facilitator, say:

- Now, we're going to play catch. Here a ball (or soft cloth), will be thrown from one person to the next, while we are seated in a circle. In this game, no one is allowed to talk unless they are holding the ball. I will ask a question, and then throw the ball to any one and that person has to say something in response to that question. Afterwards, you will then pass the ball or throw it to someone else. We will keep doing this until I ask another question.
- Good nutrition and playful interaction is very important for child development to happen. For optimal physical and cognitive development to occur, a child requires adequate nutrition, but this should occur in addition to physical and emotional stimulation from a caregiver.[e] *[You can add more information relating to nutrition or desired topic].*

---

[6] Simply known as 'passing' in the original text (Barton, 2009.Pp. 273). This activity is usually used to add more colour to learning lines in a play. However, as both hearing and receiving cues are activity, I felt it provided an interactive and stress free context in which activities can be reflected on and reviewed. This activity will be intertwined with the 'village of dreams' review sessions in the coming lessons.

Questions [to ask:]

Do you remember the story of Luyando and Luano? What do you remember about it? Was it useful to you? Did you learn anything from it? Do you think that could happen in real life? Why do you think so? And so on.

NOTE: *you can ask a participant to clarify a response and even add or direct a second question towards the person holding the ball (e.g. what do you mean by that? How? What? And so on)*

Then the play [7]***:***

Facilitator, say:

- From what we have discussed on what we recall about the story we are now going to make a simple play about *the effects of poor nutrition.*
- You can place yourselves into two groups: group A and group B [you can also guide this process to avoid the formation of cliques]. Then, group B will make a short play that shows or tells us of the negative effects of either *under-nutrition* or *inadequate stimulation* (in short choices that led to poor development in Luano).
- Once the play is done, the audience (Group A) will comment on what they saw in the play. Members of Group A will also point out, the following:
    - The choices made by the main character, in this case the 'mother.'
    - Whether the choices she made were good or poor.
    - Whether the choices made could have been different. Was there an alternative reaction or action that the character of focus could have chosen?

[7] This activity is called the 'performance game' and is a form of Forum Theatre. This is a theatrical game in which a problem is shown in an unsolved form, to which the audience, again spect-actors, is invited to suggest and enact solutions.

- What actions could be done to chance the final outcome as depicted in the play.

- Then, I will tell Group B to redo the play and as the play reaches a point where an alternative reaction was warranted I will shout stop. Here the actors must freeze, then someone from Group A will replace the character of focus and continue the play but with the suggested alternative. In this scenario, the ending changes because the reaction changes. That member of Group A now becomes a spect-actor.

Then the issues are further discussed. An element of hot seating[8] can be added then or at a later stage.

Further Questions:

Why do we sometimes make poor choices? How can we avoid doing so? Can we share our information with friends or the community? How would you like to share it? Can such a 'play' work?

INFORMATION BANK: STUNTING

What is stunting?

Stunting is when the child is very much shorter than the required or average of the children (0-59 months) in that area (e.g. country) due to long term practice of poor nutritional practices.[g] In Zambia, 40 % of children below the age of 5 years are stunted.[h] This is a critical issue because stunting is an indicator of malnutrition. It is different for both girls and boys.[ij]

Table 1: indicates average height and stunted height as noted in both girls and boys

[8] This is when the fictitious character is interviewed. Here, the audience gets to ask any questions they want of the character, but should focus on getting her to justify her behaviour in the play. As this activity can get quite intense, it would be advisable to only attempt it once the group has been together for a longer time and developed stronger ties within (when trust has been well formed).

Sex	Two year old	Three year old
**Boy**	Average height: is 86-87 cm Stunted height: at 81 cm and below.	Average height: 96 cm Stunted height: 88 cm and below.
**Girl**	Average height: 85-86 cm Stunted height: 80 cm and below.	Average height: 95 cm Stunted height: 87 cm and below.

Question [rhetorical]: Is stunting the same as dwarfism?

Response: No.

Dwarfism[k] is a genetic disorder or defect, while stunting[l] is a result of chronic malnutrition (if noticed early it can be corrected) Stunting is preventable.

Stunting is height deficit resulting from:[mn]

- Poor nutrition: unbalanced diets, low diversity of diets, junk foods, etc. Therefore, a child must have a balanced meal, high protein diets (goat milk, beans, eggs, etc.) with a variety of colourful fruits and green vegetables.

  Reminder: refer to the feeding card and feeding mat.[9]

- inadequate child stimulation and activity, plus non-responsive feeding.

[9] Refer to the charts in the CSH Nutrition promoter guide. Mothers received these in phase I of the Saving Brains project. It is however, the facilitator's own prerogative on whether to use it or not in the presence of other similar or related materials.

Inadequate child stimulation and activity is when a caregiver does not talk with the child, or play with them, or guide them through interactive activities. While non- responsive feeding is when the mother can't understand what child is saying with their actions. For instance, can't tell when child is hungry and if she does, cannot tell when child is satisfied. This may lead to overfeeding or underfeeding.

Positive Alternative: A mother needs to talk to their child, even if the child can't respond yet. Mother makes a happy face and then can point to an object around the house (or outside_ in the field) and tell the child about that object. For instance, she can begin like: Oh, look at this shiny thing. Do you know what this is? This is a pot, we use it for cooking (Can do the same with trees, maize and other crops, livestock, family pets, etc.). *If the child can, it is important for the mother to allow the child to talk or answer (verbal response) and touch the object (non-verbal response).* Mothers should have playful interactions with their child as play is the way that children learn (that is their work).

Responsive feeding can be achieved by talking to the child as you feed them and reading the non-verbal cues. It allows baby to regulate his own appetite and eating, which has been associated with a healthy weight status.

- Poor care practices, inadequate sanitation and water supply, food insecurity, low dietary diversity, inappropriate food allocation within the household (for instance, belief that children should eat less because they are small), short birth spacing which may cause mother to stop breastfeeding early because there's another baby.

Question [rhetorical]: Is stunting the same as Wasting?

Response: No

Stunting results from chronic undernourishment, which retards linear growth, whereas wasting results from inadequate nutrition over a shorter period, and underweight encompasses both stunting and wasting. Typically, growth faltering begins at about six months of age, as children

transition to solid foods that are often inadequate in quantity and quality, and increased exposure to the environment increases their likelihood of illness.

Another Sing- Along![10]

Facilitator:

- Let's all sing a song that talks about love being the key to a child's good growth and the lack of it being a cause for bad growth.
- Let's do all the actions with this song, for example, clapping hands, moving body, etc.

Growth Measuring Exercise[11]: Making the Height Measuring tape reminder

Facilitator:

- All, that is the facilitator and other mothers should work together towards making a tape that will help them to remember to check their children's height as it is essential to their development.
- You will guide and lead this process, while mothers will follow your lead as they also make their own tapes.

Materials: manila paper [number depends on number of participants][12], clear cello tape, 1 meter board ruler (or measuring tape or string), coloured markers (blue, green, purple, black or yellow),[13]Pair of scissors (or knife) **and** ball stick[14]

[10] Another song so as to reanimate the mother and child. It is most likely that after being seated or dormant for a while the children might get restless, hence the need for this interlude.

[11] This activity is inspired by the study by Rockers, P., Fink, G. & Levenson, R. (2016) Tools to improve parental recognition of developmental deficits in children. However, as we did not have access to the height charts, a height tape was used instead guided with height measurements guided by the WHO child growth charts.

[12] During this session, each A3 manila sheet created three measuring tapes, in our case. Old carton boxes can also be used, as was the case in Simakatu, thus staying true to the theme of using locally available materials and reducing litter. It is also a cost effective alternative.

[13] Charcoal can also be used instead of markers.

[14] Tree glue can be used as an alternative if accessible.

Instructions:

- Place the ruler against the chart paper, to measure a length of 1 meter. Press down hand and cut through the paper vertically. Measure a width of about 10 cm.
- Then use marker to write markings according to the ruler measurements (0 to 100 cm).
- Use a coloured marker or pencil/pen (highlight if possible) to mark the required heights for 2 year olds and 3 year olds.
- Cover the marked tape with cello tape.
- And stick it to your door post or on any wall surface
- Cut circles with a smiley face which you will stick.
- Then measure child's height against this Vertical Growth Measuring Tape. Stick a smiley face next to your child's height if it is good and stick a sad face next to your child's height if it is not good.

Note to the Caregiver [from the facilitator]: *If your child's height is good, that's awesome but you need to continue giving your child a good diverse nutritional diet and keep on stimulating his or her development in the four areas as we discussed. If it's not good (that is at 79 cm for girls and 81cm for boys) that means you may need to go to the clinic so that they can check if your child's health is okay. Do not be disheartened as this can be corrected with a good diverse diet and stimulation. Be positive minded and continue to do your part as a mother.* ***Do not forget that your child's growth has just been delayed and not stopped.***

The tortoise race°[15]

Facilitator, say:

- Now I will draw two straight lines on the ground on two opposite ends. One will be the starting point and the other finishing point on the ground using a stick.
- Then we are all (including our children), going to race. The rule is that one must move from one end to the other as slowly as they can (as slow as or slower than a tortoise). [Don't forget the funny faces.]
- The last person to reach the mark is the winner.

> **NOTE**: *The goal here is to remind mothers (caregivers) that not all children grow at the same pace; the most important thing is that you attain the main goal in the end. Which is to have a developmentally healthy and highly stimulated child as this will influence (have a positive impact) the child's future performance, academically, socially and physically. Encourage mothers to talk about how they feel.*

Homework: the village of dreams[16]

> **NOTE**: *Use this game as a means of giving homework. It is meant to make having an extra task to do a fun process.*
> ***Also keep a written record of the task each mother chooses to do***

Materials: a clay dice and three huts

Facilitator:

- Give the mother a dice, and ask her to throw it. Once it falls, whichever side it falls on represents a hut (and type of task).
- Each hut has a specific task representation, that is: 1) hut 1_ blue door or plastic, is the hut of wishes, hopes dreams the mother has for her child; 2) hut 2_ brown door or plastic,

[15] Known as 'Slow motion'

[16] I created this game as I was looking for a way to make homework fun and also have a process delegation of tasks that was left to chance. No one can accuse another of giving them a difficult task or favouring another because selection is personal. It also encourages mother to truly think about their personal goals, their goals for their child and provides an emotional support system for the mother. Although it was not assessed, it is my hope that this activity (through hut 3) will help reduce maternal depression.

is the hut of activities that mother will do with the child at home to stimulate good development, even with a busy schedule, and 3) hut 3_ green door or plastic, is the hut where the mother is asked to do something for herself that will help her be more relaxed and healthy.

- For huts 1 and 2, the tasks are highly subjective in that the mother bears all control on whether the task will be done or not. There is also no exact way of knowing if it was done or not. However, hut 2 tasks are more objective and it is easier to see if it was done. *Always note, that for hut 2_ what mother can do with the child, refer to the activity list*.

Sharing Knowledge: 15 minutes of fame[17]

Note: this activity could be optional as it depends on the group's willingness to do so. However, it is a good tool for informing the community on what was done during the intervention or programme. It also gives the participants a sense of purpose.

Facilitator, say: Now, we have learnt, recalled and even applied some information relating to early child development, it is our duty to share this information just as the chief did. Therefore, let us take this time to plan a final performance to be shared with the community.

Good day exercise [the final act][p]

- Greet or hug each other and tell each other something nice for the day. Here, each mother hugs another mother and says positive things about the other. For instance, when Jane greets Mary, she will say, 'Hi my name is Mary and I am very kind.'
- Then Mary will return the favour, by saying her name is Jane and…....

NOTE: this activity allows mothers to leave group with positive feelings about themselves and each other.

[17] Refer to Michael, J., Michel, J. & Silverberg, J. (2001) Ready for action: A popular theatre/popular education manual, page 30 for guidelines on how to plan a participatory performance.

References

[a] Izard, E. C., Youngstrom, A. E., Fine, E. S., Mostow, J. A., & Trentacosta, J. C. (2006) Emotions and the development of psychopathology in infancy and early childhood. In D. Cichetti and D. J. Cohen (Eds.), *Developmental psychopathology: Theory and method, second edition (Pp 254- 59)*. John Wiley & Sons: New Jersey.
[b] Boal, A (2005) Games for actors and non-actors, second edition. Taylor and Francis online publication. [Only the first part of this activity was used as the second part was deemed too complex at this stage.]
[c] Michael, J., Michel, J. & Silverberg, J. (2001) Ready for action: A popular theatre/popular education manual. Retrieved from: the Waterloo Public Interest Research Group website_ http//:wwwwpirg.org
[d] Barton, R. (2009) Acting: Onstage and Off, fifth edition. Wadsworth, Cengage learning: Boston.

[e] McGrath, M. & Schaffer, A. (2014) Integrating psychosocial support into nutrition programmes in West Africa during the Sahel food crisis. Retrieved from: http://www.interventionjournal.com/sites/default/files/Integrating_psychosocial_support_into_nutrition.13.pdf

[f] Boal, A (2005). Games for actors and non-actors, second edition. Taylor and Francis online publication.
[g] Caulfield, E. L., Richard, A. S., Rivera, A. J., Musgrove, P. & Black, E. R. (2002). Stunting, wasting and micronutrient disorders. Retrieved from: http://www.ncbi.nlm.nih.gov/books/NBK11784/pdf/Bookshelf_NBK11784.pdf

[h] Central Statistical Office, Ministry of Health & Inner City Fund International. (2014). *Zambia Demographic and Health Survey 2013-14*. Rockville, Maryland, USA: CSO, MoH, & ICF International.

[i]WHO (n.d) Training course on child growth assessment WHO child growth standards: boys growth record. Retrieved from: http://www.who.int/childgrowth/training

[j] WHO (n.d) training course on child growth assessment WHO child growth standards: girls growth record. Retrived from: http://www.who.int/childgrowth/training

[k] Gilberg, C. (2011) Diagnistic systems. In J L. Matson, P. Sturmey (Eds.), *International Handbook of Autism and Pervasive Developmental Disorders, Autism and Child Psychopathology Series*. Retrieved from: http://bookzz.org/book/1224654/021d5c\nhttp://bookzz.org/book/2195946/58d8a8

[l] Caulfield, E. L., Richard, A. S., Rivera, A. J., Musgrove, P. & Black, E. R. (2002). Stunting, wasting and micronutrient disorders. Retrieved from: http://www.ncbi.nlm.nih.gov/books/NBK11784/pdf/Bookshelf_NBK11784.pdf

[m] Stewart, C. P., Iannotti, L., Dewey, K. G., Michaelsen, K. F. & Onyango, A. W. (2013) Contextualising complementary feeding in a broader framework for stunting prevention. *Maternal and Child Nutrition.* 9(2), Pp.27-45.
[n] De Onis, M. & Blossner, M. (1997) WHO Global Database on Child Growth and Malnutrition. Geneva : World Health Organisation.
[o] Boal, A (2005). Games for actors and non-actors, second edition. Taylor and Francis online publication.
[p] Boal, A (2005). Games for actors and non-actors, second edition. Taylor and Francis online publication.

Lesson 3: Cognitive Development

A child's brain is like a field

Objectives:

By the end of this segment participants should:

- Know that a child learns from their environment
- Know that a child's thoughts are cultivated by things they learn from their environment through the mother, family, peer and other interactions.
- That a child's mental development is dependent on playful interactions

---

Materials Needed:

- Drum [old pots or buckets and sticks can be used in the absence of a drum], piece of sack string, 10 pieces of paper (cut out), pencil or charcoal, glue, a clay village (village of dreams), activity list and a dice.

Remember: *Always keep a bucket of clean water, a cup, soap or ash, a clean piece of cotton chitenge and dish for hand washing.*

Introduction

Facilitator:

Always remember to begin a session by:

- Greeting the participants and their children. Ask about their day, week, etc.
- Introduce yourself and briefly talk about what the meeting is about.
- Encouraging co-learners to sit in a circle, imitative of a village meeting. If possible the facilitator can have a drum[1] (old pots or buckets can serve as alternatives) present to begin the session.

---

[1] A drum is an aesthetic of popular theatre as a part of this context's oral culture, I felt it would be an effective means for signaling to the participants that the session has begun as well as getting everyone involved through sound and movement.

- Thanking participants for attending this meeting and telling them you are happy to be meeting with them.

> **NOTE**: *Always be welcoming, smile, be friendly and open (approachable). It is important to be welcoming so that participants can open up and feel free to express themselves, and also be able to approach you when they need help.*

Relaxation and trust building exercises

Facilitator:

- Ask all to stand, maintaining a circle, and do a stretch exercise.[2] For instance, stretch arms upwards, sideways, back to front, touch your toes, bend your knees, jump and try to touch the sky (reach for the heavens) and move head and neck.
- Encourage mothers to encourage children to stretch as well.

Always: pair these with breathing exercises (make funny faces and sounds with each stretch).

Facial expressions[3] help teach the child how to express emotions.[a]

> ***Remember**: We are doing this to relieve tension and relax so that the mother is to be stress free (in a good, balanced mood/ state) so that she can pay better attention to her child.*

Sing-Along Time!

Facilitator, say:

- "Does anyone know a song that talks about the effect of a mother's care on the child? For example, an action song like iluyando lupati (talks about love).
- If so, can *[insert name of person who assents]* teach it to the rest us? If not, let's make one up?

[2] This exercise was inspired by notes in Boal, A. (2005) Games for actors and non-actors and Anderson, J., Michel, J & Silverberg, J (2001) Ready for action: A popular theatre/popular education manual, encouraging a warm up before a lesson begins so as ready the learners for learning.

[3] Facial expressions are an essential survival emotional skill, especially during the first years of life, through which the child is able to elicit the caregiver's help and attention. Children also have a predisposition to attend and respond to facial expressions from their primary caregivers, hence the mother's face serves as the child's learning interface in this case.

Always encourage use of local songs.

Circle and cross (X) exercise[4b]

Facilitator:

- Here you simply draw a circle or a cross (or an X) the air. First tell mothers and their children to draw a circle with the left hand, and then tell them to draw an X with their right hand draw in air, and finally get them to draw the circle and the cross at the same time.
- The goal is **'think it, don't see it'**

Variation: try to do this exercise using your legs, while standing or sitting.

NOTE: *this cognitive exercise encourages one to be attentive as they try to do a simple but complex task. Please, be aware that most, including yourself may not be able to do this task perfectly but that is okay. We are simply trying to make one focus their attention towards achieving one goal.*

Circle of knots exercise[c]

Facilitator:

- Ask mothers to make a big circle (do not hold hands): stretch out arms until the fingertips are almost touching.
- Then tell mothers to make a small circle or a huddle until no space is left.
- Ask mothers to chant the ritual or group name (e.g. say: wise mothers! Or clap hands together or both) when circle is small.[5]
- Then make a big circle once more.

---

[4] This exercise requires both psychological and physical activation. It is extremely difficult to achieve in practice, therefore do not feel the need to compel participants nor yourself to succeed. The record so far is 3 people out of thirty according to Boal (2005). It is however a good game for exercise the mind as well as your limbs.

[5] It is important to allow participants to create a group name or ritual (activity or signal) with which they can identify with This should be done during the lesson or meeting. This should not be confused with the ritual activity (sound, gesture) in Boal, A (2005) Games for actors and non-actors..

Q&A

Q: Why are we doing this exercise?

A: We are doing it to relieve tension, to relax because a mother is supposed to be stress free (be in a good balanced mood) so that she can pay better attention to her child. Don't worry, be happy!

Village of dreams: pass the ball activity[6]

Materials needed: a ball or soft cloth

Facilitator:

- Say: "this is an activity that we will do every week. Here, we want to reflect on what we have accomplished so far. This will encourage us to commit to the activities that we chose to do during the *'village of dreams game'* with our children or by ourselves."
- Ask all to sit in a circle pass the ball or a soft cloth.
- Ask everyone to reflect on the "village of dreams" activity that we chose. Say: "Which activity did you commit to do by yourself or with your child last week? Did you do it? If yes, when is your turn to hold the ball arrives, you should say what activity you did and how. If not, you should still say the activity we were supposed to do and activity that we have done with our child."
- Now let's pass the ball.
- Then, talk about how the homework went. What was easy/difficult or fun/not fun? Discuss the challenges faced by the individuals as a group and then allow mothers to give suggestions on how to overcome these challenges before you propose new activities or alternative ways of doing them.

[6]The passing game from Barton's (2009) Acting: Onstage and off book, serves as the base for this task. I created this activity so as to create a stress free environment for reflection on homework tasks. As the mother passes the ball and responds to facilitator's questions and the discussion proceeds, the mother is disarmed and appears less likely to worry about reflection activity at hand. She is free to play with the ball (e.g. squeeze or throw in air) before passing it on.

- Say: “One important thing to remember is that we can also do these activities as we work or do chores, especially if we do not have time to spare: for example you can talk to your child about the work at hand, ask child “to help” with chores (for instance, stir this cibwantu for me).

Getting to Know Each Other

The Name Game[7d]

> Please note: This game is most effective for a group that does not know each other very well or a new group However, you can still do it even if you already know the co-participant’s names. This game helps co learn each other’s names in an interesting and active manner. It is important for the facilitator to theme the game. For example, we are going to say our names and act out what we like to do. Or act out what we wish for our children.

Facilitator, say:

- Here, one of us will say their name together with an action. For instance, I can say, ‘my name is Luyando’ and because I like birds, ‘I will imitate a bird by fluttering my wings’ (spread out arms and pretend to fly) as I say my name. [So if my name is Luyando and I like to or wish to do something (e.g. cooking) or be able to be someone in future (e.g. a kind mother). I say my name and choose an action that best describes my likes or wishes, then the co-participant either lifts or points or refers to child and encourages child to say their own name (child may imitate mother’s action)].
- The group will then repeat the mother’s and or child’s name with action. [This helps child develop sense of self (I am me) and others (they are and we are) other than his/her name and those around].
- Now let us all seat down. [In the absence of chairs use vitenges or mats.] Don’t forget to maintain the circle.

[7] Note that even though; this may be the third session, depending on choice of lesson order, no changes were made as participants of this curriculum tended to like this activity a lot. It seemed to be the most liked in this case, hence I did not feel the need to change it as yet.

Variations: game can change, over time in that facilitator can include vocal sounds, clapping, song during which co-participant shouts name, then the child's name and others repeat afterwards. Here other participants can sing back the name and do the action. Another variation could be that participant says their name, or just says out what they wish or hope for themselves and child.

True or False Exercise[8]

Facilitator:

- Before you begin you can do the ritual or circle of trust or circle and cross activity, as a warm up.
- Base the quiz on general knowledge as well as what has been taught [according to your study or programme's course content].[9]
- Draw a line on the ground with truth on one end and false on the far end and undecided on the middle.
- You will ask the question, and participants will respond by rushing to the position that states their answer (true, false or undecided).
- Then, ask participants to justify their answer. For instance, why do you think it is true or false or unknown, etc?
- Once the justification is given, those that agree with the participant are free to change position to the person whose statement they agree with. Those that do not, can maintain their original positions.
- If no one can arrive at the correct answer, you are free to provide it.

True/False Questions and Answers:

i) Children learn through play? True
Play gives the child many opportunities to think and solve problems. Also helps them note what is socially accepted and what is not. To children, play is their work.

ii) Children need store bought toys or games to enjoy playing? False
Children don't need store bought items to enjoy play, home-made ones can be enjoyed just as well. Give examples: Home-made puzzle from Magazine cut outs, drawing in the sand, trace their own hand in a home-made book, etc.

iii) Stunting (chronic energy protein malnutrition) does not affect the child negatively? False

---

[8] This game is based on the, agree or disagree, number line used in Theatre for development (TfD). I have however, modified it to suit my purpose, which is discourse, in this case.

[9] Questions in this quiz are based on content in lesson1, 2 of this course; the previous curriculum (in Saving Brains phase I: Cognitive development lesson) and Smith, K. P. & Pellegrini, A. (2013) Play Synthesis: Learning through play.

Stunting is an indicator of chronic malnutrition. The presence of stunting therefore means the child is not being well fed, or stimulated. Malnutrition can lead to severe developmental problems, e.g. stunting affects ongoing cognitive processes (attention, working memory, executive functions) during childhood.

iv) Playing with a child is a waste of time? False

It is not a waste of time to take time to play with the child as such interactions serve to teach the child not only about their environment, but how to cope when faced with certain problems and so on.

v) At 2 years, babies can listen and understand what is being said? True

Asking simple questions can help them develop, or improve their language skills.

vi) You should look into your child's eyes and talk to them while feeding? True

A mother needs to talk to her child, even if the child can't respond yet. Responsive feeding helps the mother know when the child is not only hungry but when the child is full by reading his or her facial and body language.

vii) Stunting is the same thing as dwarfism? False

Stunting is when a child is shorter than the average required age in a region, while dwarfism relates to a genetic disorder. Stunting is corrective during early childhood by a change in diet and environmental stimulation while dwarfism is not.

viii) A child will still grow healthy even if they don't have diverse diets? False

Inadequate nutrition before and after birth (in the first years of life) can seriously interfere with brain development and lead to neurological and behavioural disorders such as learning disabilities and mental retardation, stunting and many other negative defects. Child's immune system may become weak, making the child more susceptible illness. The child's physical growth is also affected.

Circular Rhythm[e]

Facilitator:

- Start by introducing an action that everyone in the circle is to repeat, one after the other until the last person in the circle.
- You can then introduce a games such as 'do like I do' or 'Guntswana'(similar to the latter) which both employ the use of circular rhythms.
- However, in these games, a person goes to the centre a sings (it's a call and response scene):

Do like I do (goes something like this)

Caller: do like I do

Responders: I do, I do

Call: do like I do

Responders: I do, I do

Caller: I do this [does an action]

Response: I do this [imitates caller's action]

Caller: I do this [does an action]

Responders: I do this [imitates caller's action]

Caller: and I do that

Respondents: and I do that

- Then the next person takes over with a different action and repeats the song.
- The game continues until all persons in the circle have participated.

NOTE: Allow participants to include similar local action games that follow this pattern.

INFORMATION BANK: COGNITIVE DEVELOPMENT

What is cognitive development?[f]

This is the development of a child's thinking skills. By age two a child knows that things continue to exist even if they cannot see them. They not only listen but understand what is being said to an extent, that's why facial expression and tone are important as they convey a positive affect, attitude to the child thus influencing his or her interest in what is being done.

Why is it important?[g]

It helps the child develop important problem solving skills as well as reasoning ability (from simple to complex form later in life).

What can be done to enhance cognitive development?

Other than good nutrition, playful interactions between mother and child, child and peers or other adults, and child and the environment are key in stimulating cognitive development.[h] It is important to note the feeding[i] and play are a child's most important events during early childhood.

Let's pretend[10]

Toss the Object[j][11]

Facilitator, say:

- Now, let's play a game. I am going to throw you an object (it can be anything, e.g. a ball, a bottle, etc depending on how I seem hold it). Remember, we are just pretending.
- Then, the person I throw it to be able to pretend to catch the imagined object with all the required actions. For instance, must be able to pretend if object is heavy and catch it as a heavy object; then toss it to another person and so on.
- You can change what the object is, with every throw.
- Make eye contact.

A Book Making Activity[k][12]

Materials: piece of sack string ,a pencil, 5-10 pieces of paper (cut out), pencil or charcoal, glue and old magazines or newspapers.

Demo from Siasikabole, Simakutu

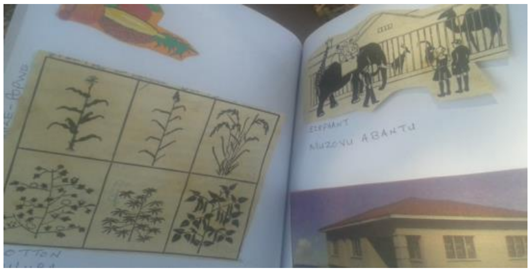

Picture taken by Mwaba Moono Chipili

---

[10] Pretend play is very important at this stage. Toddlers engage in pretend play when they imitate actions and events they have experienced in their family life. Play allows children to use their creativity while developing their imagination, dexterity and physical, cognitive and emotional strength."(Ginsberg, 2007, pp. 182-183).Thus a game like toss the object plays an important role in stimulating the mother and child's

[11] This game is good for creating awareness, improvisation skills and making creative movements that are essential in this learning process and also develop the participant's participation skills as a whole.

[12] Activity is partly derived from previous curriculum in Saving Brains phase I. This is so as this lesson was not conducted during that phase due to time factor, hence the need to do so once the study resumed. However, it 's content is also supported by Nyhout & O'Neil's (2014)studies relating to storybooks, where aspects such as structure and type of content in children's books are analysed.

Instruction:

- Cut pieces of paper into small sizes (use your palm as a guide for the size: upright for length and ¾ of your palm sideways for width).
- Then cut small holes on one edge (one long side) through which the sack string can pass, then tie it to bind the book.
- Cut the pictures into smaller pieces if large or shape them.
- You can now stick the pictures in this book with tree glue. Name each picture or write a word that best describes what is in the picture.
- In this book, you can trace[13] parts of your child, e.g. child's hand, foot or toes, etc and encourage the child to name[14] those parts.
-

NOTE: *Always remind the caregiver that it is not only important to talk to the child, but it is also important to describe and name objects or parts (while pointing to them).*

Butume Activity[15] (erranding)

Facilitator:

- You will instruct participants on what to do.

Instructions:

- First a caregiver, tells child to go and sit near an object [the child should be free to play with object if he or she choose to. Encourage caregiver not to scold her or him for that.]

[13] Tracing component is based on tracing activity from Sturm Niz, E. (n.d.) expert recommended activities that will enhance your 2 year old's development. Retrieved from: http://www.parents.com/toddlers-preschoolers/activities/indoor/activities-for-two-year-olds/. Although this task has been scaled down from the whole body just certain parts, e.g. hands.

[14] Naming of objects component is based on this study by Buris, E. S. & Brown, D. D. (2014) When all children comprehend: increasing the external validity of narrative comprehension development research; as well as kathuria and Serpell's (1999) naming task in the Panga Munthu test The mother is the child's guide, through the discovery process by the child of content in the homemade book or in their physical environment.

[15] This activity is inspired by Nsamenang's theory of African Social Ontogeny (1992), Barry & Zeitlin (2013) and Tchombe & Nsamenang (2013) in their introduction for the book *Cross-cultural Psychology: An Africentric perspective*. Their overall arguments for erranding as an African mode of socialization helped me form this activity. As this lesson looks at cognition, I felt the erranding task allowed for the stimulation of social cognition using a routine and indigenous activity, in this context.

- Then, the caregiver should tell the child to bring the object and give it to her. Caregiver can ask: what is it? What is it used for? Do you want to share it with me?
- If child says yes, s/he wants to share then caregiver should show positive affect (happy facial expressions) and praise the action. For instance: Oh wow, thank you very much. If not, caregiver can say: Oh that's okay. I see you still want to play with it. Positive facial expressions and gestures should be shown here too
- Then caregiver can ask child to share or take the object to another child or familiar adult. Here you simply observe the interactions that will happen.

NOTE: *Variations in activity can be made but keep the activities simple. Encourage caregivers not to worry if child does not do this task exactly during the first few tries. Remember he or she is only two years old, however, overtime the child will be able to do it. This task is a gate way for erranding and encourages internal verbalisations (thinking or reasoning) in the child as well as socialisation.*

GENERAL HOMEWORK

Facilitator, say:

- **try sending your child to do some simple exercise, e.g. take the cup to daddy, or draw water for mommy in a cup, do you want to help mommy cook or do you want to help daddy in the garden (apprenticeship), etc.**

Homework: the village of dreams[16]

NOTE: *Use this game as a means of giving homework. It is meant to make having an extra task to do a fun process.*

Also keep a written record of the task each mother chooses to do

Materials: a clay dice and three huts

Facilitator:

- Give the mother a dice, and ask her to throw it. Once it falls, whichever side it falls on represents a hut (and type of task).
- Each hut has a specific task representation, that is: 1) hut 1_ blue door or plastic, is the hut of wishes, hopes dreams the mother has for her child; 2) hut 2_ brown door or plastic, is the hut of activities that mother will do with the child at home to stimulate good development, even with a busy schedule, and 3) hut 3_ green door or plastic, is the hut where the mother is asked to do something for herself that will help her be more relaxed and healthy.
- For huts 1 and 2, the tasks are highly subjective in that the mother bears all control on whether the task will be done or not.
- There is also no exact way of knowing if it was done or not. However, hut 2 tasks are more objective and it is easier to see if it was done. *Always note, that for hut 2_ what mother can do with the child, refer to the activity list*.

[16] I created this game so the delegation of tasks was left to chance. No one can accuse another of giving them a difficult task or favouring another because selection is personal. It also encourages mother to truly think about their personal goals, their goals for their child and provides an emotional support system for the mother. Although it was not assessed, it is my hope that this activity (through hut 3) will help reduce maternal depression.

Sharing Knowledge: 15 minutes of fame[17]

Note: this activity could be optional as it depends on the group's willingness to do so. However, it is a good tool for informing the community on what was done during the intervention or programme. It also gives the participants a sense of purpose.

Facilitator, say:

Now, we have learnt, recalled and even applied some information relating to early child development, it is our duty to share this information just as the chief did. Therefore, let us take this time to plan a final performance to be shared with the community.

Good day exercise [the final act][l]

- Greet or hug each other and tell each other something nice for the day. Here, each mother hugs another mother and says positive things about the other. For instance, when Jane greets Mary, she will say, 'Hi my name is Mary and I am very kind.'
- Then May will return the favour, by saying her name is Jane and…....

Note: this allows mothers to leave group with positive feelings about themselves and each other.

---

[17] Refer to Michael, J., Michel, J. & Silverberg, J. (2001) Ready for action: A popular theatre/popular education manual, page 30 for guidelines on how to plan a participatory performance.

References

[a] Izard, E. C., Youngstrom, A. E., Fine, E. S., Mostow, J. A., & Trentacosta, J. C. (2006) Emotions and the development of psychopathology in infancy and early childhood. In D. Cichetti and D. J. Cohen (Eds.), *Developmental psychopathology: Theory and method, second edition (Pp 254- 59)*. John Wiley & Sons: New Jersey.

[b] Boal, A. (2005) Games for actors and non-actors, second edition. Taylor and Francis online publication. [Only the first part of this activity was used as the second part was deemed too complex at this stage.]

[c] Boal, A (2005) Games for actors and non-actors, second edition. Taylor & Francis online publication.

[d] Michael, J., Michel, J. & Silverberg, J. (2001) Ready for action: A popular theatre/popular education manual. Retrieved from: the Waterloo Public Interest Research Group website_ http//:wwwwpirg.org

[e] Boal, A. (2005) Games for actors and non-actors, second edition. Taylor & Francis online publication.

[f] Moussoui, el N. & Braster, S. (2011) Perceptions and Practices of Stimulating Children's Cognitive Development Among Moroccan Immigrant Mothers. *Journal of child and family studies*, 20 (3), Pp. 370-83.

[g] UNICEF (n.d) Early dchildhood development: the key to a full and productive life. Retrieved from: http://www.unicef.org/dprk/ecd.pdf

[h] Smith, K. P. & Pellegrini, A. (2013) Play Synthesis: Learning through play, In Tremblay, R. E., Boivin, M., Peters, R. De V. (Eds) *Encyclopedia on Early Childhood Development* [online]. Montreal, Quebec: Centre of Excellence for Early Childhood Development and Strategic Knowledge Cluster on Early Child Development, i-iii. Available at: http://www.childencyclopedia.com/pages/PDF/synthesis-play.pdf. Accessed [20th April, 2016]

[i] Yi Hui Liu & Stein, M. T. (2013) Feeding Behaviour of infants and young children and its impact on child psychosocial and emotional. Retrieved from: http://www.child-encyclopedia.com/child-nutrition/according-experts/feeding-behaviour-infants-and-young-children-and-its-impact-child

[j] Michael, J., Michel, J. & Silverberg, J. (2001) Ready for action: A popular theatre/popular education manual. Retrieved from: http://wwwpirg.org

[k] Nyhout, A. & O' Neil, K. D. (2014) Storybooks aren't just for fun: narrative and non-narrative picture books foster equal amounts of generic language during mother-toddler book sharing. *Frontiers in Psychology,5 (325).* Retrieved from: http://www.frontiersin.org/journal/10.3389/fpsyg.2014.00325/

[l] Boal, A (2005). Games for actors and non-actors, second edition. Taylor and Francis online publication.

Lesson 4: Language Development

The knowledge of speech

Objectives:

By the end of this session participants should:

- Recall that children learn to speak by being spoken to.
- Know that children think through language.
- Know that children need to be talked to in order for them to grow well.
- That conversation and songs are important for child's memory and thinking.

Materials needed:

- Drum [old pots or buckets and sticks can be used in the absence of a drum], two spoons, a piece of string (or bark or tape), a clay village (village of dreams), activity list and a dice.

> ***Remember:*** *Always keep a bucket of clean water, a cup, soap or ash, a clean piece of cotton chitenge and dish for hand washing.*

Introduction

Facilitator:

Always remember to:

- Greet the mothers and their children as they walk in.
- always be friendly and welcoming
- Encourage all to sit in circle, as you continue this "village meeting."
- Beat the drum to signal that the meeting has begun.
- Thank mothers for coming to today's session

> **NOTE**: *Always be welcoming, smile, be friendly and open (approachable). It is important to be welcoming so that participants can open up and feel free to express themselves, and also be able to approach you when they need help.*

Formation: The nature choir

Facilitator:

- Ask all to stand, maintaining the circle, and do a stretch exercise: stretch arms upwards, sideways, back to front, touch your toes, bend your knees, jump and try to

touch the sky (reach for the heavens) and move head and neck.[1] Encourage mothers to encourage children to stretch as well!

[This exercise then transitions into the zoo house game]

The Zoo House Game[2a]

Facilitator:

- Ask, each mother one at a time to imitate the sounds of nature (e.g. trees, crickets, cows, goats, chickens, cockerels, guinea fowls, wild birds, etc.) or sounds they have heard in the mornings as they wake up. They should accompany them with a funny face!
- Once that is done, ask the mothers to move closer together if they have made the same sound so you can form a few groups.
- Each group *must* encourage the children[3] to do the same sound too,
- Now the group is ready for a choir!
- I will be the first conductor, then anyone who wants to can takeover once I step away from the front join the choir.
- Tell each group to go one at a time, then together, make it loud, make it soft etc.
- Let's have fun!

Q & A

Q: *Why did we do these series of activities?*

A: First, we did it to start off with good energy. It is always important to communicate good energy to the kids and to ourselves. We also want to remind ourselves that we are a group. In terms of child development, facial expressions help teach the child how to express emotions; sounds create a basis for child to develop their language.

[1] This is the relaxation exercise. Here it is merged with a series of activities that will lead to the nature choir.
[2] This task has been adapted to suit context. Since most mothers could not read, the writing of animal to imitate was eliminated. The zoo house has also been combined with sound environment and the human orchestra activities of popular theatre.

[3] Mothers come with their children to these lessons. Therefore, each group here will consist of mothers and children. As this curriculum includes both, parents must encourage the children to be involved in the process.

Circle of trust exercise[4b]

Facilitator:

- Ask mothers to make a big circle (do not hold hands): stretch out arms until the fingertips are almost touching.
- Then tell mothers to make a small circle or a huddle until no space is left.
- Ask mothers to chant the ritual or group name once the circle is small.[5]
- Then make a big circle once more.

Q&A

Q: Why are we doing this exercise?
A: We are doing it to relieve tension, to relax because a mother is supposed to be stress free (be in a good balanced mood) so that she can pay better attention to her child. Don't worry, be happy!

NOTE: *This activity helps boost the morale and reminds learners that these sessions have a positive goal. You can also use this exercise any time the morale is low or when learners seem tired or attention seems to be wandering to bring their focus back to the lesson at hand.*

Village of dreams: pass the ball activity[6]

Materials: a ball or soft cloth

Facilitator:

- Say: "this is an activity that we will do every week. Here, we want to reflect on what we have accomplished so far. This will encourage us to commit to the activities that we chose to do during the *'village of dreams game'* with our children or by ourselves."
- Ask all to sit in a circle pass the ball or a soft cloth
- Ask everyone to reflect on the "village of dreams" activity that we chose. Say: "Which activity did you commit to do by yourself or with your child last week? Did you do it? If yes, when is your turn to hold the ball arrives, you should say what activity you did and how. If not, you should still say the activity we were supposed to do and activity that we have done with our child."

[4] Also known as the Joe Egg
[5] It is important to allow participants to create a group name or ritual (activity or signal) with which they can identify with This should be done during the lesson or meeting. This should not be confused with the ritual activity (sound, gesture) in Boal, A (2005) Games for actors and non-actors..
[6]The passing game from Barton's (2009) Acting: Onstage and off book, serves as the base for this task. I created this activity so as to create a stress free environment for reflection on homework tasks. As the mother passes the ball and responds to facilitator's questions and the discussion proceeds, the mother is disarmed and appears less likely to worry about reflection activity at hand. She is free to play with the ball (e.g. squeeze or throw in air) before passing it on.

- Now let's pass the ball.
- Then, talk about how the homework went. What was easy/difficult or fun/not fun? Discuss the challenges faced by the individuals as a group and then allow mothers to give suggestions on how to overcome these challenges before you propose new activities or alternative ways of doing them.
- Say: "One important thing to remember is that we can also do these activities as we work or do chores, especially if we do not have time to spare: for example you can talk to your child about the work at hand, ask child "to help" with chores (for instance, put this plate in that dish for me)."

Rhyme-Along Time!

Facilitator:

- Say: "Does anyone know a rhyming[c] song, choral (ciyabilo) or poem suitable for children?"
- If so, can *[insert name of person who assents]* teach it to the rest us? If not, let's make one up?

  Please encourage use of local rhymes, chorals or poems.[7]

Q&A

Q: *Why are rhymes and songs important?*

A: Songs are important because children learn words and language best when it is musical and when it has a rhyme. This is how children learn language best

NOTE*: You have the task of looking for traditional poems, chorals or rhymes that are suitable for children in your context study before this session occurs.*

[7] Please note that children learn words best through rhyme (Read, 2014; zerotothree.org) and music (zerotothree.org). Read asserts that rhyme and the pause effect (in story books) has a positive effect on the child's ability to learn words or terms, hence it can be argued that when dealing with language development_ the rhyme acts as an effective stimulatory factor. Poems were included as they bare the pause effect which is useful in a context where most mothers are not literary. These aesthetics are also indigenous to the African context, thus there inclusion allows the use of local funds of knowledge as described by Banda (2008, 2009).

INFORMATION BANK: LANGUAGE DEVELOPMENT

Language in Children

At age two, children are able to speak.[d] Their vocabulary may range from 2-3 words to 50 words and more. However this vocabulary is only increased or created as a result of verbal interactions with caregivers, peers and other adults (or older children) around them.[e] The more actively interactive a discussion is the more likely the child will remember the words.

All mothers talk to their children; perhaps some do it more than others. *Why?* Because, most see talking to babies as pointless: he or she is a just a baby so what do they know? But that is not true. **Babies learn through being spoken to, this encourages not only thinking (internal verbalization) but language (external verbalization) as well.**[f] In order for language to develop mothers should alternate turn-taking rhythm and rhythmic chorusing and bodily stimulation.[g] This means aside from asking child their names, telling them what it means, talking about their family in positive terms (social roles), and allowing them a turn to talk as well (respond): singing to children with actions is helpful as well.[h]

Importance of language stimulation[i]

Talking to children is crucial for their development. For instance talking about past events helps improve memory: children learn how to structure their memory narratives (recount their memories to other people) in a culturally appropriate way.

Conversations with the child help the child's emerging self concept and their understanding of self and others. Do not forget that stimulation in all areas of development is important in preventing stunting. In this case verbal stimulation must occur in line with non-verbal stimulation (actions) in order to make a more effective impact. For example, singing songs with actions and so on.

Games for language development

Facilitator:

- After the information bank session. Follow up with this activity to help drive the information learned home.
- Encourage each mother and child to pair up, in their own space, but not too far to hear what is being said.

Makani Ajilo (Stories from the Past)[8j]

Facilitator, say:

- Now that you're comfortable, we are going to do an activity that is very important for your child's language development.
- After today, you can continue to do this type of activity at home as well.
- Today, I want you to talk to your child about two events that happened no longer than 4 weeks ago.
- For instance, talk about the time grandma came to visit or when you went to the market or visited a relative or when a relative came to visit.
- You can talk about events that happened when you were with your child or those that happened when you were not with your child. You can talk about things that happened to you or you can talk about what you saw.
- Make facial and hand gestures as you talk to your child. You can make funny faces; make sounds (e.g. cow's moo or cat's meow, or how daddy sounded).
- Be in character, pretend to be the person or the animal. Make this conversation as visual as possible.

This is how children learn, by looking at your face and connecting with the words.

Suggested Activity for Language Development

> **NOTE:** *This activity will not be done as part of the group but individually. Explain the task as described below and encourage the mothers to practice it during the next two weeks. Tell mothers that they are free to share information on how it goes at the next meeting.*

Responsive Feeding Exercise[k9]

Materials: two spoons and tree bark or sack string

Facilitator, say:

- In the second lesson we learnt that responsive feeding helps in the prevention of stunting (especially with relation to underfeeding). A mother must therefore monitor what her child eats and the amount.
- Here your *task* is to talk to your child as you feed them.

[8] Tulviste et al (2013) argue that children at this age have no proper concept of the future, hence stories told must be based on past events that happened as this helps the child form perceptions about the people and things around them. It is important to note that as the context of study is similar to that of the Tulviste African rural context it was easy to adopt the instructions used to this context.

[9] Adapted from the responsive feeding activity on: http://www.parents.com

- You can pretend that the spoon is an train trying to deliver goods (the food) into a special destination (the child's mouth). Be excited, happy!
- You must tie two spoons tightly together by their handles, so that when the child eats the mother can also use the other end to feed herself.
- This will encourage the child to eat as mummy is doing it too.

Walk, stop, justify[l10]

Facilitator, say:

- First, let's walk the way we usually walk on a daily basis [your normal walk]. Here we will be imitating ourselves.
- Then, when I say STOP you have to stay frozen the position, the command found you in. For example, if your leg is up when I say STOP, leave it up.
- Then, I will come and ask you why you are in that position. You response should make sense. You cannot say it is because I said stop, but you can say, 'I saw an ant so I lifted my leg' or 'I am playing hop scotch (locally known as pade) with my child.' In this case you position has been justified.

Variations can be added, for instance: walk how you walk when you are tired, happy, and sad, bored or feeling lazy. Allow mothers and their children to walk around,

- Then tell them to stop.

> **NOTE**: *This activity helps the mother look to herself in decision making instead of blindly following what others are doing. Here, we encourage the mother to reason within herself before seeking outside help. The child also learns not only about body parts and positions but that their several states of being and that those actions have explanations. As the child is still young they not fully grasp this concept, however it a fun activity that also helps stimulate their physical development and incorporates listening, observation and response.*

General Homework: Storytelling (Twaano) is important[11]

Facilitator, say:

- Storytelling is important. So next time you are at home you can tell your children a folktale.

[10] This task encourages language development through action and explanation of the said action.

[11] Shiel, G., Cregan, Á., McGough, A. & Archer, P. (2012) assert that shared reading practice studies show that oral language (mainly receptive vocabulary) has relatively strong effects for children between the ages of 2-3 (our target audience). As our context is not very literary, I felt a traditional activity could be used instead to elicit the same results.

- Don't forget to use lots of gestures, facial and vocal expressions. You should also point to objects in the environment that maybe the same as objects or characters in your story.
- If possible, ask the grandmother or grandfather or father to tell the story instead. You can then ask, the children (not only the two year olds) what was the moral? Or what they learnt from the story?
- Then, when we meet for the next lesson each mother can tell us how this exercise went.

Homework: the village of dreams[12]

NOTE: *Use this game as a means of giving homework. It is meant to make having an extra task to do a fun process.*
Also keep a written record of the task each mother chooses to do

Materials: a clay dice and three huts

Facilitator:

- Give the mother a dice, and ask her to throw it. Once it falls, whichever side it falls on represents a hut (and type of task).
- Each hut has a specific task representation, that is: 1) hut 1_ blue door or plastic, is the hut of wishes, hopes dreams the mother has for her child; 2) hut 2_ brown door or plastic, is the hut of activities that mother will do with the child at home to stimulate good development, even with a busy schedule, and 3) hut 3_ green door or plastic, is the hut where the mother is asked to do something for herself that will help her be more relaxed and healthy.
- For huts 1 and 2, the tasks are highly subjective in that the mother bears all control on whether the task will be done or not. There is also no exact way of knowing if it was done or not. However, hut 2 tasks are more objective and it is easier to see if it was done. *Always note, that for hut 2_ what mother can do with the child, refer to the activity list*.

Sharing Knowledge: 15 minutes of fame[13]

Note: this activity could be optional as it depends on the group's willingness to do so. However, it is a good tool for informing the community on what was done during the intervention or programme. It also gives the participants a sense of purpose.

[12] I created this game as I was looking for a way to make homework fun and also have a process delegation of tasks that was left to chance. No one can accuse another of giving them a difficult task or favouring another because selection is personal. It also encourages mother to truly think about their personal goals, their goals for their child and provides an emotional support system for the mother. Although it was not assessed, it is my hope that this activity (through hut 3) will help reduce maternal depression.
[13] Refer to Michael, J., Michel, J. & Silverberg, J. (2001) Ready for action: A popular theatre/popular education manual, page 30 for guidelines on how to plan a participatory performance.

Facilitator, say:
-Now, we have learnt, recalled and even applied some information relating to early child development, it is our duty to share this information just as the chief did. Therefore, let us take this time to plan a final performance to be shared with the community.

Good day exercise [the final act][m]

- Greet or hug each other and tell each other something nice for the day. Here, each mother hugs another mother and says positive things about the other. For instance, when Jane greets Mary, she will say, 'Hi my name is Mary and I am very kind.'
- Then Mary will return the favour, by saying her name is Jane and…....

NOTE: this allows mothers to leave the group with positive feelings about themselves and each other.

Have a lovely day…

References

[a] Michael, J., Michel, J. & Silverberg, J. (2001) Ready for action: A popular theatre/popular education manual. Retrieved from: http://wwwwpirg.org

[b] Boal, A (2005) Games for actors and non-actors, second edition. Taylor and Francis online publication. [Only the first part of this activity was used as the second part was deemed too complex at this stage.]

[c] Read, K (2014) Cues the smooze: rhyme, pausing and prediction help children learn new words from story books. Frontiers *in psychology, 5 (149)*. Retrieved from: *http://www.frontiersin.org/Psychology/editorialboard*

[d] UNICEF (n.d) Early dchildhood development: the key to a full and productive life. Retrieved from: http://www.unicef.org/dprk/ecd.pdf

[e] Bloom, P. (2001) Precis of how children learn the meaning of words. *Behavioural and brain science*, 24, Pp. 1095-1103

[f] Demuthm, C.(2013) Protoconversation and protosong as infant's socialization environment. In Tchombe, M. S. T., Nsamenang, B. A., Keller, H. & Fulop, M. (Eds) *Cross-cultural Psychology: An Africentric perspective*. Design house: Limbe, Cameroon.

[g] Demuthm, C.(2013) Protoconversation and protosong as infant's socialization environment. In Tchombe, M. S. T., Nsamenang, B. A., Keller, H. & Fulop, M. (Eds) *Cross-cultural Psychology: An Africentric perspective*. Design house: Limbe, Cameroon.

[h] Demuthm, C.(2013) Protoconversation and protosong as infant's socialization environment. In Tchombe, M. S. T., Nsamenang, B. A., Keller, H. & Fulop, M. (Eds) *Cross-cultural Psychology: An Africentric perspective*. Design house: Limbe, Cameroon.

[i] Tulviste, T., Tougu, P., Keller, H., Schroder, L., & De Geer, B. (2013) Different faces of autonomy: Mother-child past event conversations across cultural developmental pathways. In Tchombe, M. S. T., Nsamenang, B. A., Keller, H. & Fulop, M. (Eds) *Cross-cultural Psychology: An Africentric perspective*. Design house: Limbe, Cameroon.

[j] Tulviste, T., Tougu, P., Keller, H., Schroder, L., & De Geer, B. (2013) Different faces of autonomy: Mother-child past event conversations across cultural developmental pathways. In Tchombe, M. S. T., Nsamenang, B. A., Keller, H. & Fulop, M. (Eds) *Cross-cultural Psychology: An Africentric perspective*. Design house: Limbe, Cameroon; and Schroder

[k] Castle, J. (n.d.). Why you need to practice responsive feeding. Retrieved from: https://www.bundoo.com/articles/why-you-need-to-practice-responsive-feeding/

[l] Boal, A (2005). Games for actors and non-actors, second edition. Taylor and Francis online publication.

[m] Boal, A (2005). Games for actors and non-actors, second edition. Taylor and Francis online publication.

Lesson 5: Water, Sanitation and Hygiene.

A mother's role is to protect [It's About Us, It's All In Our Hands]

Objectives:

By the end of this lesson the participant should:

- Recall the importance of hygiene and sanitation
- Have a glance at the effects of poor hygiene and sanitation
- Be able to know which practices are not hygienic.
- Be able to pinpoint alternative sanitation and hygiene practices
- Be able to relate child growth with hygiene practices

Materials needed:

- Drum [old pots or buckets and sticks can be used in the absence of a drum], two buckets, sticks, sieve (the one used to when making mealie meal), a clean cloth, river sand, charcoal , a clay village (village of dreams), activity list and a clay dice.

> ***Remember:*** *Always keep a bucket of clean water, a cup, soap or ash, a clean piece of cotton chitenge and dish for hand washing.*

Introduction

Facilitator:
Always remember to:

- Greet the mothers and their children as they walk in.
- always be friendly and welcoming
- Encourage all to sit in circle, as you continue this "village meeting."
- Beat the drum to signal that the meeting has begun.
- Thank mothers for coming to today's session

> **NOTE**: *Always be welcoming, smile, be friendly and open (approachable). It is important to be welcoming so that participants can open up and feel free to express themselves, and also be able to approach you when they need help or want to make suggestions.*

Relaxation and trust building exercises

Facilitator:

- Ask all to stand, maintaining a circle, and do a stretch exercise.[1] For instance, stretch arms upwards, sideways, back to front, touch your toes, bend your knees, jump and try to touch the sky (reach for the heavens) and move head and neck.
- Encourage mothers to encourage children to stretch as well.

Always: pair these with breathing exercises (make funny faces and sounds with each stretch).

Facial expressions[2] help teach the child how to express emotions.[a]

> ***Remember****: We are doing this to relieve tension and relax so that the mother is to be stress free (in a good, balanced mood/ state) so that she can pay better attention to her child.*

Sing-Along Time!

Facilitator, say:

- "Does anyone know a song that talks about cleanliness?
- If so, can *[insert name of person who assents]* teach it to the rest us? If not, let's make one.

Always encourage use of local songs.

Circle of trust exercise[3b]

Facilitator:

- Ask mothers to make a big circle (do not hold hands): stretch out arms until the fingertips are almost touching.
- Then tell mothers to make a small circle or a huddle until no space is left.
- Ask mothers to chant the ritual or group name once the circle is small.[4]
- Then make a big circle once more.

---

[1] This exercise was inspired by notes in Boal, A. (2005) Games for actors and non-actors and Anderson, J., Michel, J & Silverberg, J (2001) Ready for action: A popular theatre/popular education manual, encouraging a warm up before a lesson begins so as ready the learners for learning.

[2] Facial expressions are an essential survival emotional skill, especially during the first years of life, through which the child is able to elicit the caregiver's help and attention. Children also have a predisposition to attend and respond to facial expressions from their primary caregivers, hence the mother's face serves as the child's learning interface in this case.

[3] Also known as the Joe Egg

[4] It is important to allow participants to create a group name or ritual (activity or signal) with which they can identify with This should be done during the lesson or meeting. This should not be confused with the ritual activity (sound, gesture) in Boal, A (2005) Games for actors and non-actors..

Q&A

Q: Why are we doing this exercise?
A: We are doing it to relieve tension, to relax because a mother is supposed to be stress free (be in a good balanced mood) so that she can pay better attention to her child. Don't worry, be happy!

NOTE: *This activity helps boost the morale and reminds learners that these sessions have a positive goal. You can also use this exercise any time the morale is low or when learners seem tired or attention seems to be wandering to bring their focus back to the lesson at hand.*

A Game about Sanitation

Kalambe a Bonga[c5]

Facilitator:

- Here four circles will be drawn on the ground. These four circles should be placed at four corners that will act as safety zones (Clean water, Healthy food, Use pit latrine and Hospital).
- One person will be chosen using picki na piki doli[6] exercise and that person will be it (the germ). [It is at this point that the aspect of tag is introduced].[7]
- The tagger stays at the centre of the circles. She can only tag someone if that person is out of the circle (safety zones)
- The (for each person) mission is to run from one circle to the next without being tagged. If you are tagged (touched) then you are it, and the game starts again and so on.

Questions to ask after game:
How do you feel? What do you think this game is trying to tell us?

Remember that*: when one person is affected it is likely that all may become affected. Therefore, it is important that we take the issue of water, sanitation and hygiene seriously.*

[5] Known as tag or touch and rounder's in English. I merged these two games as I felt it could be more effective in that form to teach about the ills of poor sanitation and positive aspects about good sanitation. This idea was further expanded on and some of the kinks practically worked out as we tried to play it, by Erricah, Lydia and Susan [CHWs key players in the participatory component of this curriculum].

[6] Game used to pick the one who will be last e.g. 'there's a pie in the bedroom.' Any suitable game of chance can be used to be who gets to be the germ.

[7] The person who is it plays the role of the tagger (as in tag/touch) or the ball in rounders

INFORMATION BANK: WATER SAFETY, SANITATION AND HYGIENE

Facilitator:

- Ask participants, what are some of the problems related to water safety, sanitation and hygiene we see in our communities?[8]
- Give them time to respond
- So now, before we begin to talk about why having clean water, good hygiene and good sanitation are important, let us go through the following definitions:[de]

  Water safety *refers to ways of having or getting access to clean water supply.*

  Sanitation *refers to ways that promote proper disposal of human and animal wastes, proper use of toilet and avoiding open space defecation.*

  Hygiene *refers to bodily cleanliness [personal grooming] issues such as bathing, brushing teeth etc.*

Why should this be important to us?[f]

The absence of clean **water, hygiene and sanitation** has negative effects on the nutrition status of children especially when they are very young. Access to clean water and adequate sanitation are important in reducing child mortality therefore increasing chances of survival, especially given the prevalence of diarrhoea and waterborne diseases. Learning about water, hygiene and sanitation is important because it will helps use know or increase our knowledge on good sanitary health practices in preventative and sustainable responses.

Water supply and sanitation improvement, together with improvements in people's behaviours can have very positive effects on the people and their health through reduction of diseases such as diarrhoeal diseases, intestinal (usually worm related) infections and skin diseases. Therefore, creating clean environments for children averts threats to their health and supports the best chance at a prosperous life by reducing disease and child mortality.

What is the Link between water safety, sanitation and hygiene to stunting?[g]

Poor water, sanitation, and hygiene slow the growth of children. Poor growth (stunting) that occurs in about one quarter to one third of children below 5 years is as a result of intestinal infections most of which are as a result poor sanitation and hygiene. This intestinal infections and diarrhoea diseases lead to loss or inability to absorb much needed nutrients thus the child becomes malnourished.

---

[8] In this context of study issues included: poor use of pit latrines, poor hand washing practice, children not bathed frequently left in dirty nappies, mattresses not aired especially when bed has been wetted, open defaecation, poor water sources, and many more.

Questions: *What should be done about this? What can we do? How can we change these behaviours?*

More about Cleanliness: the Sanitation Game[9]

Facilitator:

- Here you are going to draw a maze (lines that will form a box or square) on the ground, make sure there is a lot of space.
- It should look squared, and there should be a starting point and a finishing point. The goal for each mother is to get to the end (the finishing line) where it will show that your family has grown well, due to good sanitation.
- *Y*ou will ask questions and each mother has to give an answer, for them to move forward. [You can ask all, the same question if participants are many and you have few questions. They can then whisper the answer.][10] Or each mother can be asked a different question if you have enough questions, this can also shorten the games' duration.
- If the answer is correct she and her child will move forward to the next corner, if not they are to stay stagnant.
- **A variation can be added**, whereby if the answer is way off the mark [according to your scale] or a leakage ["cheating"] is given, the mother-child pair has to go back by a step.

NOTE: *This game has no winners or losers, to say, therefore it only finishes when all have managed to reach the finishing line. The target is so that each participant reaches the inner box of good health. It is helpful in getting the group to think on the ultimate course goal.*

[9] The levels in this game was inspired by the snakes and ladders game, in that an individual moves from one step to the next after overcoming a hurdle. However, in this context, it seemed too complex an activity for both the mother and child to do; hence the simple maze was adopted. The child can easily follow the mother's steps.

[10] This can be done although it is advisable to source as many statements as possible on the target topic.

Statements for the Game [True or false][h]

a. **Open defaecation is not good; therefore people should not do it.**

   Answer: True

   This is because excreta left in the open often finds its way into sources of drinking water and food and may lead to disease such as typhoid, cholera, diarrhoea and dysentery. Flies are main spreaders of this.

b. **The latrine must be used always for urinating and defaecation.**

   Answer: True

   To prevent the spread of excreta related diseases.

c. **I should drink water from safe sources only.**

   Answer: True

   To prevent diseases like worms, cholera, diarrhoea, typhoid and to remain healthy. Protected springs and boreholes, treated pipe water and protected hand dug wells are good sources of safe water.

d. **It is not possible to make water safe to drink.**

   Answer: False

   By boiling it or adding chlorine (solid or liquid) water can be treated and made safe to drink.

e. **We must live in a clean and healthy environment.**

   Answer: True

   To prevent hygiene and sanitation related diseases and enjoy good health for us and our children.

f. **It is not possible to make our environment a clean and healthy place.**

   Answer: False

   If we all get involved, by taking individual and collective actions such as sweeping, cleaning, proper disposal of human and animal wastes, proper use of toilet and avoiding open space defecation we can take a step towards make our environment clean and healthy. A mother protects her child, and her community.

g. **Good Hygiene means keeping yourself and your surroundings clean.**

Answer: True

Hygiene is the practice of keeping yourself and your surroundings clean, especially to avoid illness or the spread of preventable diseases.

h. **I should wash my hands regularly.**

Answer: True

Your hands can carry seen and unseen dirt that cause diseases. Always wash hands with soap or ash and water to prevent infections.

i. **It is not important for me to wash my hands many times; that is wasting water.**

Answer: False

It is important to wash hands as often as possible, especially after:

- *After going to the toilet and after changing baby's nappies*
- *Before handling and eating food*
- *After blowing or cleaning nose*

Good hand washing helps prevent a lot of diseases

j. **I only need to use soap when washing my hands for them to be truly clean.**

Answer: False

You can use ash for hand washing if soap is not available because ash helps kill germs and bacteria. You can even use it for washing your plates and pots.

k. **I should take bath everyday if possible.**

Answer: True

To prevent skin diseases and bad body odour

l. **I should clean my teeth daily.**

Answer: True

To prevent tooth decay and mouth odour. You can chew the end of a mulberry stick and use that as your toothbrush, if you don't have one.

m. **Children can participate in these activities.**

Answer: True

Children can be encouraged to develop knowledge, attitudes and skills that support the adoption of good hygiene behaviours and healthy living.

Children can be agents of change to their peers, families and their communities and promote the importance of drinking safe water, good hygiene practice and use of safe sanitation facilities.

n. Only fathers should be responsible for hygiene and sanitation in the family.

Answer: False

Every member of the family has responsibility for Hygiene and sanitation.

Questions [to ask after the game]:

How did this exercise make you feel? What did you think about? Was is helpful?

NOTE: *when this game is done feel free to read out the statements again, but this time accompanied by the answer and additional explanations.*

As you discuss the game, remind mothers about these Questions: What should be done? What can we do? How can we change these behaviours?

You can also add, "mothers help me solve this problem: If my child wets the bed what can I do clean up? What should I do for the child?"

Discussion: The Sanitation problem

Facilitator, say:

- **Let's have a discussion!** I know that there are many issues that affect us when it comes to the topic of water safety, sanitation and hygiene; none of which can be addressed without your involvement. You are the ones who know what happens in your community.
- Therefore, let's take this time to look at them in detail.

Questions:
What are the issues truly affecting us in our communities? What things have you seen other people do that you know need to be addressed? What can we [you and me] do to solve this issue?

NOTE: *Feel free to add more questions to help mothers bring out the real issues at hand.*

- I'm sure all of you still remember Luano: she or he is the child that did not grow well because of poor care; poor feeding and now we have just found out, today after visiting her home, that poor water safety, hygiene and sanitation were also a contributing problem.

- ***Now, let's make a short play!*** You are going to make a play about the poor sanitation practices in Luano's home which lead to her sickness, her family's sickness and finally the whole village.

> **NOTE: Refer to the play_ the life of Luano in lesson 2.** *Create another forum as conducted in lesson 2.*

Joe Egg:[11i] Whose Problem is it?

Facilitator:

- You are going to lead an exercise that helps answer the above question.
- Here a mother goes to the centre, closes their eyes while the other mothers stand behind her, as she falls backwards.
- The other mothers, are going to catch her as she falls and then propel her gently back towards the centre, where she doesn't come to rest, because she starts to fall in another direction, and thus it goes on.
- It is very important that there are always at least three people at any given moment taking care of the person in the middle.
- At the end, if you want, you can roll the person around the circle, instead of immediately standing her up in the middle again.
- The goal is not only for the remaining mothers to catch her as she falls, but that she should have trust enough to know that they will catch her when she falls.

> **Questions:**
>
> *What did you feel? What do you think is being said to us in this activity? Did you learn anything from this exercise? Does it have any connection to the above question?*
>
> **NOTE***: you are doing this exercise to show that all should take part in resolving issues in our communities. We should all take responsibility in creating the change we want to see in our environment.*

[11] Also known as the trust circle in Boal's (2005) arsenal of theatre of the oppressed. Used to build trust in the group, in this case it is used to encourage community support and involvement in resolving issues such as sanitation

Suggested home activity for clean water

Natural water purifier[12j]

Materials: two buckets, sticks, sieve (the one used to when making mealie meal), a clean cloth, river sand and charcoal.

Instructions:

- Make holes in one bucket, then place sticks at the bottom of the bucket so that the all meet at the centre;
- then place a sieve [like the one used for sieving mealiemeal] on top of sticks, and place clean cloth on top of sieve.
- Place the river sand and charcoal on top of the cloth.
- Get a second bucket (without holes) and place sticks on top of that bucket so that they extend outward. These sticks must be strong and firm, enough to sit the punctured bucket.
- Then pour water into the top bucket, and clean safe to drink water will filter into bucket below.
- Now you have germ free, clean water!

Homework: the village of dreams[13]

NOTE: *Use this game as a means of giving homework. It is meant to make having an extra task to do a fun process.*
Also keep a written record of the task each mother chooses to do

Materials: a clay dice and three huts

Facilitator:

- Give the mother a dice, and ask her to throw it. Once it falls, whichever side it falls on represents a hut (and type of task).
- Each hut has a specific task representation, that is: 1) hut 1_ blue door or plastic, is the hut of wishes, hopes dreams the mother has for her child; 2) hut 2_ brown door or plastic, is the hut of activities that mother will do with the child at home to stimulate good development, even with a busy schedule, and 3) hut 3_ green door or plastic, is the hut where the mother is asked to do something for herself that will help her be more relaxed and healthy.

[12] During the participatory workshop, a question on alternative methods of water purification was paused. After brainstorming, Susan [CHW] brought to light that during one of her training endeavors they taught under 'Peacecorps' how to purify water as described above. We all reached a consensus to try this method, as at now during a focus group discussion with mothers from the Saving brains study, it was said that this alternative was very useful and helped mothers have water that had no aftertaste.

[13] I created this game as I was looking for a way to make homework fun and also have a process delegation of tasks that was left to chance. No one can accuse another of giving them a difficult task or favouring another because selection is personal. It also encourages mother to truly think about their personal goals, their goals for their child and provides an emotional support system for the mother. Although it was not assessed, it is my hope that this activity (through hut 3) will help reduce maternal depression.

- For huts 1 and 2, the tasks are highly subjective in that the mother bears all control on whether the task will be done or not. There is also no exact way of knowing if it was done or not. However, hut 2 tasks are more objective and it is easier to see if it was done.
- *Always note, that for hut 2_ what mother can do with the child, refer to the activity list*.

Sharing Knowledge: 15 minutes of fame[14]

Note: this activity could be optional as it depends on the group's willingness to do so. However, it is a good tool for informing the community on what was done during the intervention or programme. It also gives the participants a sense of purpose.

Facilitator, say:
-Now, we have learnt, recalled and even applied some information relating to early child development, it is our duty to share this information just as the chief did. Therefore, let us take this time to plan a final performance to be shared with the community.

Good day exercise [the final act][k]

- Greet or hug each other and tell each other something nice for the day. Here, each mother hugs another mother and says positive things about the other. For instance, when Jane greets Mary, she will say, 'Hi my name is Mary and I am very kind.'
- Then Mary will return the favour, by saying her name is Jane and….....

> **NOTE**: *this allows mothers to leave group with positive feelings about themselves and each other.*

Have a lovely, germ free Day…

[14] Refer to Michael, J., Michel, J. & Silverberg, J. (2001) Ready for action: A popular theatre/popular education manual, page 30 for guidelines on how to plan a participatory performance.

[a] Izard, E. C., Youngstrom, A. E., Fine, E. S., Mostow, J. A., & Trentacosta, J. C. (2006) Emotions and the development of psychopathology in infancy and early childhood. In D. Cichetti and D. J. Cohen (Eds.), *Developmental psychopathology: Theory and method, second edition (Pp 254- 59)*. John Wiley & Sons: New Jersey.

[b] Boal, A (2005) Games for actors and non-actors, second edition. Taylor and Francis online publication. [Only the first part of this activity was used as the second part was deemed too complex at this stage.]

[c] Sport In Action (2004) Sport in the development process: Leadership manual. SIA.

[d] The National Food and Nutrition Commission of Zambia (n.d.) Improving water supply, sanitation and hygiene: A prerequisite to improving child nutrition policy brief. Retrieved from: http://www.parliament.gov.zm/sites/default/files/documents/committee_reports/Water%20and%20Sanitation%20Report.pdf

[e] UNICEF & WaterAid (2008) frequently asked questions: sanitation and hygiene.

[f] UNICEF (n.d.). Water, sanitation and hygiene. Retrieved from: http://www.unicef.org/cambodia/8.WASH.pdf

[g] The National Food and Nutrition Commission of Zambia (n.d.) Improving water supply, sanitation and hygiene: A prerequisite to improving child nutrition policy brief. Retrieved from: http://www.parliament.gov.zm/sites/default/files/documents/committee_reports/Water%20and%20Sanitation%20Report.pdf

[h] UNICEF & Water Aid (2008) frequently asked questions: sanitation and hygiene.

[i] Boal, A. (2005) Games for actors and non-actors, second edition. Taylor & Francis online publication

[j] Chen, Chilufya & Young (2012) appropriate technology manual: a step by step guide to building appropriate technologies. Zambia: peace corps/Dlab [need to further clarify]

[k] Boal, A (2005). Games for actors and non-actors, second edition. Taylor and Francis online publication.

Lesson 6: Physical Development.

Activating your whole body

Objectives:

By the end of the session participants should:

- Recall that exercise is important for the child's physical growth
- Reflect on the role that physical development has in a child's growth.
- Know that this growth is obtained through play.

Materials needed:

- Drum [old pots or buckets and sticks can be used in the absence of a drum], a clay village (village of dreams), activity list and a dice.

> ***Remember*:** *Always keep a bucket of clean water, a cup, soap or ash, a clean piece of cotton chitenge and dish for hand washing.*

Introduction

Facilitator:
Always remember to:

- Greet the mothers and their children as they walk in.
- always be friendly and welcoming
- Encourage all to sit in circle, as you continue this "village meeting."
- Beat the drum to signal that the meeting has begun.
- Thank mothers for coming to today's session

> **NOTE**: *Always be welcoming, smile, be friendly and open (approachable). It is important to be welcoming so that participants can open up and feel free to express themselves, and also be able to approach you when they need help or have suggestions.*

Game: the Action game

Facilitator:

- Ask all to stand, maintaining the circle, and do a stretch exercise: stretch arms upwards, sideways, back to front, touch your toes, bend your knees, jump and try to touch the sky (reach for the heavens) and move head and neck. Encourage mothers to encourage children to

stretch as well!

[This exercise transitions into the action game]

The Action Game[1]

Facilitator:

- Ask each mother (one at a time) to simply go into the middle and act out or imitate an animal or something they like.
- Then tell everyone else to imitate what was done [like in the name game only this time it is just actions]. Encourage mothers to try to get their child to imitate their action at this point.
- As mothers imitate the animal they like, they is free to make animal sounds (e.g. oink, oink), not forgetting the all important funny faces.

Q & A

Q: Why did we do this game?

A: It is always important to stretch and get ready to learn. In terms of child development, facial expressions help teach the child how to express emotions, sounds help the child with language and the physical activities create a basis for child to develop their muscles (physical development).

Sing-Along Time!

Facilitator, say:

- "Does anyone know a traditional song that talks about or helps with physical development?"
- If so, can *[insert name of person who assents]* teach it to the rest us? If not, let's make one up? Feel free!

Always encourage use of local songs and creativity[2].

Circle of trust exercise[3a]

Facilitator:

- Ask mothers to make a big circle (do not hold hands): stretch out arms until the fingertips are almost touching.
- Then tell mothers to make a small circle or a huddle until no space is left.
- Ask mothers to chant the ritual or group name once the circle is small.[4]
- Then make a big circle once more.

---

[1] This game is inspired by Boal's (2005) circle of rhythms of Toronto and Anderson, J., Michel, J & Silverberg, J's (2001) Who are you?/ What are you doing improvisation exercises. However, I eliminated the circle of rhythm 'pairs' and the who are you?/ What are you doing?, verbal interlude to suit the overall theme of the physical activation of the body in this lesson.

[2] Creating their own songs may help increase their sense of ownership of the programme at hand.

[3] Only the first part of this activity, the elastic circle is used.

[4] It is important to allow participants to create a group name or ritual (activity or signal) with which they can identify with This should be done during the lesson or meeting. This should not be confused with the ritual activity (sound, gesture) in Boal, A (2005) Games for actors and non-actors..

Q&A

Q: Why are we doing this exercise?
A: We are doing it to relieve tension, to relax because a mother is supposed to be stress free (be in a good balanced mood) so that she can pay better attention to her child. Don't worry, be happy!

NOTE: *This activity helps boost the morale and reminds learners that these sessions have a positive goal. You can also use this exercise any time the morale is low.*

Village of dreams: pass the ball activity[5]

Materials: a ball or soft cloth

Facilitator:

- Say: "this is an activity that we will do every week. Here, we want to reflect on what we have accomplished so far. This will encourage us to commit to the activities that we chose to do during the *'village of dreams game'* with our children or by ourselves."
- Ask all to sit in a circle pass the ball or a soft cloth.
- Ask everyone to reflect on the "village of dreams" activity that we chose. Say: "Which activity did you commit to do by yourself or with your child last week? Did you do it? If yes, when is your turn to hold the ball arrives, you should say what activity you did and how. If not, you should still say the activity we were supposed to do and activity that we have done with our child."
- Now let's pass the ball.
- Then, talk about how the homework went. What was easy/difficult or fun/not fun? Discuss the challenges faced by the individuals as a group and then allow mothers to give suggestions on how to overcome these challenges before you propose new activities or alternative ways of doing them.
- Say: "One important thing to remember is that we can also do these activities as we work or do chores, especially if we do not have time to spare: for example you can talk to your child about the work at hand, ask child "to help" with chores (for instance, stir this cibwantu for me).

[5]The 'passing' game from Barton's (2009) Acting: Onstage and off, serves as the base for this task. I created this activity so as to create a stress free environment for reflection on homework tasks. As the mother passes the ball and responds to facilitator's questions and the discussion proceeds, the mother is disarmed and appears less likely to worry about reflection activity at hand. She is free to play with the ball (e.g. squeeze or throw in air) before passing it on.

INFORMATION BANK: PHYSICAL DEVELOPMENT

Physical development[b]

This refers to the development of muscles and motor (gross and fine) skills as a child grows.

Importance of Physical Development[c]

Physical development is important because it provides children with the abilities they need to explore and interact with the world around them. It involves activities such as running around outside, jumping on the bed, holding a cup or spoon, parent's finger or using a stick to drawing in the sand or colour in a book if possible.

Physical development makes play possible, which is essential for the child's entire development through stimulation. Don't forget that play is the work of childhood.[d]

Developmental Milestones: knowing what my child should be able to do[e]

Knowing the developmental milestones helps a mother know whether her child is growing at a typical rate. Since our children are two years old we know that physically they should be able to:

- ✓ Run, jump, hop and walk well. May also be able to kick ball, though not that well.
- ✓ Able to catch (upper body) and throw ball.
- ✓ Hold spoons, container, cups and can stack a tower of plates or cups (at least 6 or 7 plus).[6] Moulding nshima during a real meal or pretend clay meal (fine motor skills). This so as by this stage they have gained control of hands and fingers.
- ✓ Can climb chicken ladders (no stairs in our homes).[7] But most two year olds are able to or will be able to climb chicken keep stairs (not high) by this age and later on can climb into the maize shelter which also has a ladder (stairs).

> ***NOTE**: children grow at individual rates with some being faster or slower than others. Therefore encourage mothers not to be quick to worry (as long a diverse balanced diet, clean environment and stimulation is available).*

[6] Adapted to suit this context as towers are not available in this context

[7] It was noted that climbing stairs is not an applicable milestone in this context. However, during the participatory workshop the CHWs advised that chicken steps be the alternative as these are quite common the this rural setup and that most children are able to climb these steps by age two.

Physical developmental benefits of play[f]

• Positive emotions increase the efficiency of immune, endocrine, and cardiovascular systems.

• Decreases stress, fatigue, injury, and depression due to deprivation in children.

• Increases range of motion, agility, coordination, balance, flexibility, and fine and gross motor exploration

> **NOTE**: *Advise parents [mothers in this case] that this does not mean that a mother should leave child to play all day. Simply, that a mother can allocate a specific amount of time for the child to play with peers on a regular basis. A parent should also take the time to play with her child.*

Games for Physical Development

Facilitator, say:

- We are now going to play a few games that help enhance physical development.
- Let's begin:

The Duck tag[8g]

- Here, each mother is going stick their knees tight together with feet facing sideways so that they waddle like a duck. The arms will also be squeezed against the body with hands sticking out like flippers.
- Everyone is "it" all will waddle like ducks and try to tag each other.
- In this game, you tag others by waddling over and tagging them lightly with your flippers.
- The goal here is for each one to try to tag others without moving arms away from your body.
- The game continues until all have managed to tag each other.

This game is not only useful for stress relief and interaction but improves reflexes and running skills (especially in children) as well as control.[h] Encourage children to imitate these actions. It's also fun!

The Crazy walk[9]

Facilitator:

- Ask group members to take a breath and stretch a bit.
- Then tell them to spread out.
- Then ask all to walk around the space slowly while walking the way they usually walk on a daily basis.

[8] Renamed this game from Penguin tag to Duck tag as the penguin is a foreign bird in this context and none seemed to know what it is.

[9] The Crazy walk is combination of 'as you like it', 'imitating others', 'the demon' , 'dissociate coordinated movements' and 'mirror sequence' exercises from Boal's (2005) arsenal of theatre of the oppressed.

- Each one should make sure that no two legs are on the ground at the same time. For example, if the left leg is down then the right leg should be up and vice-versa.
- Participants can then observe how others are walking and select one person to imitate. One can follow behind the selected person so that she appears like shadow or face the selected person so that she acts as her reflection (like a mirror).These imitations can be accompanied by sounds and facial expressions.
- Then participants can increase speed to walk at a normal pace, and the pace can alternate between fast and slow movements. Hand and body movements should be present and visible.
- Now ask participants to show a happy walk, an angry walk, a bored walk, a lazy walk, a sad walk.
- Then ask them to walk as if they are crazy or how a crazy person walks. For instance, walk in zig-zag motion, never straight, throw hands in all directions, and make crazy looking faces and animal noises. These can be slow or fast or normally paced.
- *If you want, variations can also be made to imitate how a chicken, duck, dog or monkey, etc walks as well as the sounds they make.*
- Afterwards, mothers can stretch to relax and form the circle of knots in conclusion.

Questions

How did you feel? What do you think about these exercise games? Why did we do them?

After getting responses re-emphasise the aspect physical development and its importance For instance, in these games: we exercised our gross motor skills, self control, thinking skills, pretend play abilities and so many other aspects of development which are essential in child growth.

NOTE: *Always make sure that there is enough space so that no one hurts the other, especially during the crazy walk.*

Homework: the village of dreams[10]

NOTE: *Use this game as a means of giving homework. It is meant to make having an extra task to do a fun process.*
Also keep a written record of the task each mother chooses to do

Materials: a clay dice and three huts

Facilitator:

- Give the mother a dice, and ask her to throw it. Once it falls, whichever side it falls on represents a hut (and type of task).
- Each hut has a specific task representation, that is: 1) hut 1_ blue door or plastic, is the hut of wishes, hopes dreams the mother has for her child; 2) hut 2_ brown door or plastic, is the hut

[10] I created this game as I was looking for a way to make homework fun and also have a process delegation of tasks that was left to chance. No one can accuse another of giving them a difficult task or favouring another because selection is personal. It also encourages mother to truly think about their personal goals, their goals for their child and provides an emotional support system for the mother. Although it was not assessed, it is my hope that this activity (through hut 3) will help reduce maternal depression.

of activities that mother will do with the child at home to stimulate good development, even with a busy schedule, and 3) hut 3_ green door or plastic, is the hut where the mother is asked to do something for herself that will help her be more relaxed and healthy.

- For huts 1 and 2, the tasks are highly subjective in that the mother bears all control on whether the task will be done or not. There is also no exact way of knowing if it was done or not. However, hut 2 tasks are more objective and it is easier to see if it was done. *Always note, that for hut 2_ what mother can do with the child, refer to the activity list*.

Sharing Knowledge: 15 minutes of fame[11]

Note: this activity could be optional as it depends on the group's willingness to do so. However, it is a good tool for informing the community on what was done during the intervention or programme. It also gives the participants a sense of purpose.

Facilitator, say:
-Now, we have learnt, recalled and even applied some information relating to early child development, it is our duty to share this information just as the chief did. Therefore, let us take this time to plan a final performance to be shared with the community.

Good day exercise [the final act][i]

- Greet or hug each other and tell each other something nice for the day. Here, each mother hugs another mother and says positive things about the other. For instance, when Jane greets Mary, she will say, 'Hi my name is Mary and I am very kind.'
- Then Mary will return the favour, by saying her name is Jane and…....

> ***NOTE***: this allows mothers to leave the group with positive feelings about themselves and each other.

Have a lovely day…

[11] Refer to Michael, J., Michel, J. & Silverberg, J. (2001) Ready for action: A popular theatre/popular education manual, page 30 for guidelines on how to plan a participatory performance.

[a] Boal, A (2005) Games for actors and non-actors, second edition. Taylor and Francis online publication. [Only the first part of this activity was used as the second part was deemed too complex at this stage.]

[b] Brotherson, S. (2006) Understanding physical development in young children. North Dakota State University Extension Service

[c] Brotherson, S. (2006) Understanding physical development in young children. North Dakota State University Extension Service

[d] The power of play: learning through play from birth to three years. Retrieved from: http://www.zerotothree.org

[e] The Early Childhood Direction Centre (2012) Developmental checklists birth to five. Retrieved from: http://ecdc.syr.edu/wp-content/uploads/2013/01/Developmental_checklists_Updated2012.pdf

[f] Whitebread, D., Basilio, M., Kuvalja, M. & Verma, M. (2012) The importance of play: A report on the value of children's play with a series of policy recommendations. Retrieved from: http://www.importanceofplay.eu/IMG/pdf/dr_david_whitebread_-_the_importance_of_play.pdf

[g] Michael, J., Michel, J. & Silverberg, J. (2001) Ready for action: A popular theatre/popular education manual. Retrieved from: http://wwwpirg.org

[h] Michael, J., Michel, J. & Silverberg, J. (2001) Ready for action: A popular theatre/popular education manual. Retrieved from: http://wwwpirg.org

[i] Boal, A (2005). Games for actors and non-actors, second edition. Taylor & Francis online publication.

Lesson 7: Diverse Diets for the Growing Child

How and what a child eats

Objectives:

By the end of this session, participants should:

- Recall that a balanced diet is important for the child's healthy growth
- Recall that diverse diets will help prevent stunting
- Know that children model the parent's eating behaviours as well.
- Be able make a balanced meal using locally available foods

Materials Needed:

- Drum [old pots or buckets and sticks can be used in the absence of a drum], piece of sack string, 10 pieces of paper (cut out), pencil or charcoal, glue, a clay village (village of dreams), activity list and a dice.

> ***Remember:*** *Always keep a bucket of clean water, a cup, soap or ash, a clean piece of cotton chitenge and dish for hand washing.*

Introduction

Facilitator:
Always remember to:

- Greet the participants and their children. Ask about their day, week, etc.
- Introduce yourself and briefly talk about what the meeting is about.
- Encourage co-learners to sit in a circle, imitative of a village meeting. If possible the facilitator can have a drum[1] (old pots or buckets can serve as alternatives) present to begin the session.
- Thank participants for attending this meeting and telling them you are happy to be meeting with them.

[1] A drum is an aesthetic of popular theatre as a part of this context's oral culture, I felt it would be an effective means for signaling to the participants that the session has began as well as getting everyone involved through sound and movement.

> **NOTE**: *Always be welcoming, smile, be friendly and open (approachable). It is important to be welcoming so that participants can open up and feel free to express themselves, and also be able*

Relaxation and trust building exercises

Facilitator:

- Ask all to stand, maintaining the circle, and do a stretch exercise: stretch arms upwards, sideways, back to front, touch your toes, bend your knees, jump and try to touch the sky (reach for the heavens) and move head and neck. Encourage mothers to encourage children to stretch as well!

Always: pair these with breathing exercises (make funny faces and sounds with each stretch).

Remember facial expressions[2] help teach the child how to express emotions.[a]

> ***Remember****: We are doing this to relieve tension and relax so that the mother is to be stress free (in a good, balanced mood/ state) so that she can pay better attention to her child.*

Sing-Along Time

Facilitator, say:

- “Does anyone know an action song that talks about healthy foods/ local foods? Or maybe a song that talks about the good outcomes of good care/ feeding?”

- If so, can *[insert name of person who assents]* teach it to the rest us? If not, let's make one up?

  Always encourage use of local songs along with mother-child interaction.

Circle of knots exercise[3b]

Facilitator:

- Ask mothers to make a big circle (do not hold hands): stretch out arms until the fingertips are almost touching.
- Then tell mothers to make a small circle or a huddle until no space is left.
- Ask mothers to chant the ritual or group name once the circle is small.[4]
- Then make a big circle once more.

---

[2] Facial expressions are an essential survival emotional skill, especially during the first years of life, through which the child is able to elicit the caregiver’s help and attention. Children also have a predisposition to attend and respond to facial expressions from their primary caregivers, hence the mother’s face serves as the child’s learning interface in this case.

[3] Only the first part of this activity, the elastic circle is used.

[4] It is important to allow participants to create a group name or ritual (activity or signal) with which they can identify with This should be done during the lesson or meeting. This should not be confused with the ritual activity (sound, gesture) in Boal, A (2005) Games for actors and non-actors..

Q&A

Q: Why are we doing this exercise?
A: We are doing it to relieve tension, to relax because a mother is supposed to be stress free (be in a good balanced mood) so that she can pay better attention to her child. Don't worry, be happy!

NOTE: *This activity helps boost the morale and reminds learners that these sessions have a positive goal. You can also use this exercise any time the morale is low or when learners seem tired or attention seems to be wandering to bring their focus back to the lesson at hand.*

A child of hope

The Name Game[5c]

Facilitator, say:

- By now, we all know this game. Many of us have added or made a few changes and that is okay.
- Today, each one of us will say our child's names instead. We are going to pretend that we are our children.
- For example, one of us will enter the circle say our child's name, then shout or act out what we want our children to be, and the rest will follow once asked: "who is she?"
- The group will then repeat the "child's" name with action. [This helps mother view the child as something more than a dependent but someone with a bright future and a place in this our world].
- Now let us all seat down. [In the absence of chairs use vitenges or mats.] Don't forget to maintain the circle.

---

[5] In this lesson, the name game incorporates the element of pretend play to encourage mothers to plan what they want for that child or who they hope this child will become. These individual goals may therefore enhance the role the mother plays in her child's development as she begins to see that hope as a possible reality.

Village of dreams: pass the ball activity[6]

Materials: a ball or soft cloth

Facilitator:

- Say: "this is an activity that we will do every week. Here, we want to reflect on what we have accomplished so far. This will encourage us to commit to the activities that we chose to do during the *'village of dreams game'* with our children or by ourselves."
- Ask all to sit in a circle pass the ball or a soft cloth.
- Ask everyone to reflect on the "village of dreams" activity that we chose. Say: "Which activity did you commit to do by yourself or with your child last week? Did you do it? If yes, when is your turn to hold the ball arrives, you should say what activity you did and how. If not, you should still say the activity we were supposed to do and activity that we have done with our child."
- Now let's pass the ball.
- Then, talk about how the homework went. What was easy/difficult or fun/not fun? Discuss the challenges faced by the individuals as a group and then allow mothers to give suggestions on how to overcome these challenges before you propose new activities or alternative ways of doing them.
- Say: "One important thing to remember is that we can also do these activities as we work or do chores, especially if we do not have time to spare: for example you can talk to your child about the work at hand, ask child "to help" with chores (for instance, stir this cibwantu for me).

INFORMATION BANK: NUTRITION AND FEEDING

Facilitator, begin:

- *Everybody eats! In order to survive we all need food, this includes our children as well. We know that at this stage [age 2 years] most of our children cannot only attempt to feed themselves, but they eat what the family eats as well. Feeding and play are important for our children's proper growth and development*[d].

[6]The passing game from Barton's (2009) Acting: Onstage and off book, serves as the base for this task. I created this activity so as to create a stress free environment for reflection on homework tasks. As the mother passes the ball and responds to facilitator's questions and the discussion proceeds, the mother is disarmed and appears less likely to worry about reflection activity at hand. She is free to play with the ball (e.g. squeeze or throw in air) before passing it on.

Importance of feeding

Feeding is a primary event in the infant or young child's life.[e] Growing children, therefore, need plenty of energy (calories) and nutrients (protein, fat, carbohydrate, vitamins and minerals) to ensure they grow and develop well.[f] They also need to eat a good variety of foods, including lots of fruits and vegetables, to make sure they get all the other important dietary components they need.[g] In the previous curriculum, we established that there are three food categories: i) body building, ii) energy giving foods and iii) protective foods.[7] Therefore, when preparing a meal a mother must try to include all of them to make a healthy diet. A good appetite will usually ensure that children get enough energy from the food they eat.

Note that the nutritional status of the child also has an impact on the child's relationship with the primary caregiver and is predictive or indicative of an unhealthy relationship with the caregiver.[h] This is so as how a mother feeds the child and her behaviour during feeding time teach the child how they are to react. If you're shouting at the child or saying mean things because they aren't eating the food or he or she is taking their time it will likely create a negative impact in the child.

Feeding behaviours

A person's eating habits are established as young as 2 to 3 years old.[i] This means a child who eats a diet rich in vegetables at a young age is more likely to eat vegetables as an adult. To help children develop patterns of healthy eating from an early age, it is important that the food and eating patterns to which children are exposed – both at home and outside the home – are those which promote positive attitudes and enjoyment of healthy food.[j]

The teacher in this case is the parent. **Remember, our responsive feeding exercise in lesson 4.** The feeding experience provides not only sustenance but also an opportunity for learning for both the mother and her child.[8k] It affects not only children's physical growth and health but also their psychosocial and emotional development. Responsive feeding therefore, helps the child learn to speak and also teaches the child good eating behaviours. Parents are who they learn from.

Feeding times and portions

Question: How many meals should a two year old child eat a day?

Answer: 5 meals (that is 3 full meals and 2 snacks).[l]

Our children are at least over two years old, hence, they should have the above stated number of meals. We can use the feeding bowl for age appropriate feeding portions.[9] *In the absence of these you can start with giving the child a small portion and tell her that if she is still hungry you will give her some more.* Variety is as important as quantity.

[7]This is referring to the curriculum used in the first phase of the Saving Brains study.

[8] The mother learns to read her child's hungry or satiety cues. She also learns to read her child's discomfort levels and attitudes towards certain foods, while the child learns her mother's facial expressions (social cues) and language expressions.

[9] During the previous curriculum mothers were given feeding bowls that helped them know what portions a child should have per meal according to age. In this phase we had no access to such materials, thus half the levelled adult meal was opted for, in line with Serrano and Powell's (2014) recommendations.

To ensure a child has appetite, the best thing would be to allow them to play as much as possible so that they are hungry at meal time.[m] Remember, play is children's work. Active children tend to be able to get all the nutrients necessary if availed by the mother. Use smaller plates, spoons, and cups for children, to prevent overeating.[n]

> ***NOTE**: Review the feeding card & mat used in the CSH Nutrition Promoter guide for further clarification on feeding portions and meals.*

Link of diets to stunting

Stunting and other forms of undernutrition are clearly a major contributing factor to child mortality, disease and disability. For example, a severely stunted child faces a four times higher risk of dying, and a severely wasted child is at least a nine times higher risk.[o] Undernutrition early in life clearly has major consequences for future educational, income and productivity outcomes. Stunting is associated with poor school achievement and poor school performance.[p] This means that focus on the child's diet is very important as well. A balanced diet along with physical activity (attained through play) will help in the child be healthy.

Creating a balanced diet

There are many local fruits and vegetables that can be used as snacks instead of processed foods. Children must be discouraged from eating non-nutritious foods (e.g. soft drinks, jiggies, sweets, etc.). Instead, you can give your child fruits[10] (e.g. ½ a banana, a cup of Mbula, Masau, a mango etc.). You can even make Mubuyu (baobab), Mbula, or cibwantu drink not only for baby but the entire house hold as well. The mother's nutrition and the entire household's are important too.

> **Questions to ask**:
>
> What do think about this information? Does it make sense to you? If not, where are you not clear? What other local foods can be used as snacks for our children?

A Play of Sorts: Luano versus Luyando's Mother

Facilitator, say:

- We are now going to make a play about an unhealthy child, Luano. Luano was not well fed, resulting in malnutrition.
- When the play ends. As we have done other times, anyone [not part of the play] is free point out what was wrong. Then the play will be acted again, but this time with the correction.

A Twist in the tale….

- Then, we are going to pick the two [or more] mothers, who acted as Luano and Luyando's mothers and interview them in their characters.

[10] Used Serrano, E. & Powell, A.'s (2014) article as a guide for snack measurements.

- Remember, the stop, walk, justify game. This is almost the same. Only here, the actresses [Luano and Luyando's mothers] will have to explain why they did what they did in the play.

> **NOTE**: *the idea is to create a safe target of discussion without pointing directly at real life individuals. The hope is that as this discussion progresses mothers will always want to associate with the good mother while distancing themselves from the bad one, not only in word but in practice as well. It is also a good way of promoting self-reflection through external observation.*

Discussion: Examples of healthy foods.[11]

Facilitator, say:

- Now we are going to take the time to talk about local foods that are important when creating a healthy a diet.

> **NOTE**: *This discussion though guided by the facilitator is left to the participants. The goal here is see what they know about the contents of a balanced meal. You can theme each round, for example you can say: let's talk about body building foods, or energy or protective foods. Or let's say the snacks that are good for our children. If you want, you can vary it and say we are going to talk about unhealthy foods instead.*

The ball of remembrance[12q]

Facilitator, say:

- Here, we will talk about what we know or remember about healthy foods. I will ask a question, then throw the ball to one of you.

> **Some questions to ask:**
> What do you know about good nutrition? Or what do you think good nutrition means? How many food groups are there? What makes a balanced diet? And so on. Feel free to include more.

- Then that person has to mention a food that is good or healthy for eating.
- After answer is given, I will ask all to either agree or disagree with that answer. Then we will continue with the game.
- Now that we recall, the importance of a healthy diet, in the next lesson we are going to prepare a meal.

---

[11] This discussion is one by the participants for the participants. No information is shared unless participants completely have no idea what healthy or unhealthy foods are.

[12] This activity is named for the passing technique used in acting during character development or learning script (Barton, 2009). In this case, I felt it would work well in gauging how much the participants knew on child development.

Note: This game can be accompanied by a song and person with the ball when song ends says something.

General Homework

Facilitator:

- Delegate and ask mothers to come with the ingredients: kaplanga (moringa), mundyoli (pumpkin leaves pounded groundnuts), and mealiemeal.[13] For example, each mother can bring a cup of mealiemeal; while some can bring a cup of pounded groundnuts, some pumpkin leaves, 2 tomatoes & onions, quarter a cup of cooking oil, a bowl of moringa leaves and so on, depending on what is available that season.

NOTE: *the facilitator should find a place to do the cooking demonstration from, e.g. their home before the set dates for that session.*

Homework: the village of dreams[14]

NOTE: *Use this game as a means of giving homework. It is meant to make having an extra task to do a fun process.*
Also keep a written record of the task each mother chooses to do

Materials: a clay dice and three huts

Facilitator:

- Give the mother a dice, and ask her to throw it. Once it falls, whichever side it falls on represents a hut (and type of task).
- Each hut has a specific task representation, that is: 1) hut 1_ blue door or plastic, is the hut of wishes, hopes dreams the mother has for her child; 2) hut 2_ brown door or plastic, is the hut of activities that mother will do with the child at home to stimulate good development, even with a busy schedule, and 3) hut 3_ green door or plastic, is the hut where the mother is asked to do something for herself that will help her be more relaxed and healthy.
- For huts 1 and 2, the tasks are highly subjective in that the mother bears all control on whether the task will be done or not. There is also no exact way of knowing if it was done or not. However, hut 2 tasks are more objective and it is easier to see if it was done. *Always note, that for hut 2_ what mother can do with the child, refer to the activity list*.

[13] This menu was created around what was available that season. The main participatory group and mother heads (leaders of the mother groups in the Saving Brains) agreed upon these ingredients.

[14] I created this game so the delegation of tasks was left to chance. No one can accuse another of giving them a difficult task or favouring another because selection is personal. It also encourages mother to truly think about their personal goals, their goals for their child and provides an emotional support system for the mother. Although it was not assessed, it is my hope that this activity (through hut 3) will help reduce maternal depression.

Sharing Knowledge: 15 minutes of fame[15]

Note: this activity could be optional as it depends on the group's willingness to do so. However, it is a good tool for informing the community on what was done during the intervention or programme. It also gives the participants a sense of purpose.

Facilitator, say:

-Now, we have learnt, recalled and even applied some information relating to early child development, it is our duty to share this information just as the chief did. Therefore, let us take this time to plan a final performance to be shared with the community.

Good day exercise [the final act][r]

- Greet or hug each other and tell each other something nice for the day. Here, each mother hugs another mother and says positive things about the other. For instance, when Jane greets Mary, she will say, 'Hi my name is Mary and I am very kind.'
- Then May will return the favour, by saying her name is Jane and…....

***Note**: this allows mothers to leave group with positive feelings about themselves and each other.*

Have a lovely day....

[15] Refer to Michael, J., Michel, J. & Silverberg, J. (2001) Ready for action: A popular theatre/popular education manual, page 30 for guidelines on how to plan a participatory performance.

[a] Izard, E. C., Youngstrom, A. E., Fine, E. S., Mostow, J. A., & Trentacosta, J. C. (2006) Emotions and the development of psychopathology in infancy and early childhood. In D. Cichetti and D. J. Cohen (Eds.), *Developmental psychopathology: Theory and method, second edition (Pp 254- 59)*. John Wiley & Sons: New Jersey.
[b] Boal, A (2005) Games for actors and non-actors, second edition. Taylor and Francis online publication. [Only the first part of this activity was used as the second part was deemed too complex at this stage.]
[c] Michael, J., Michel, J. & Silverberg, J. (2001) Ready for action: A popular theatre/popular education manual. Retrieved from: http://wwwwpirg.org

[d] Eating well for 1-4 year olds. Retrieved form: http://www.cwt-chew.org.uk/eatingwell1-4years/

[e] Yi Hui Liu & Stein, M. T. (2013). Feeding behaviour of infants and young children and its impact on child psychosocial and emotional development. *Encyclopaedia on early childhood development*. Retrieved from: http://www.child-encyclopedia.com/child-nutrition/according-experts/feeding-behaviour-infants-and-young-children-and-its-impact-child

[f] Eating well for 1-4 year olds. Retrieved form: http://www.cwt-chew.org.uk/eatingwell1-4years/

[g] Eating well for 1-4 year olds. Retrieved form: http://www.cwt-chew.org.uk/eatingwell1-4years/

[h] Waters & Valenzuela (1999). Explaining disorganized attachment: Clues from research on mild-to-moderately undernourished children in Chile. In J. Solomon & C. George (Eds). *Attachment Disorganization*. New York: Guilford Press.

[i] Serrano & Powell (2013). Healthy Eating for Children Ages 2 to 5 Years Old: A Guide for Parents and Caregivers. Virginia Tech/State University. Retrieved from: http://www.ext.vt.edu

[j] Eating well for 1-4 year olds. Retrieved form: http://www.cwt-chew.org.uk/eatingwell1-4years/

[k]Yi Hui Liu & Stein, M. T. (2013). Feeding behaviour of infants and young children and its impact on child psychosocial and emotional development. *Encyclopaedia on early childhood development*. Retrieved from: http://www.child-encyclopedia.com/child-nutrition/according-experts/feeding-behaviour-infants-and-young-children-and-its-impact-child
[l] Communications Support for Health (n.d) CSH Nutrition promoter guide 1st 1000 most critical days: the best start to life. CSH & USAID: Zambia.

[m] Serrano & Powell (2013). Healthy Eating for Children Ages 2 to 5 Years Old: A Guide for Parents and Caregivers. Virginia Tech/State University. Retrieved from: http://www.ext.vt.edu

[n] Serrano & Powell (2013). Healthy Eating for Children Ages 2 to 5 Years Old: A Guide for Parents and Caregivers. Virginia Tech/State University. Retrieved from: http://www.ext.vt.edu

[o] Victora, G. C., Adair, L., Fall, C., Hallal, C. P., Martorell, R., Richter, L. & Sachdev, S. H. *(2008)*Maternal and child undernutrition: consequences for adult health and human capital, *Lancet,* 371, pp. 340–57. DOI:10.1016/S0140-6736(07)61692-4

[p] Grantham-McGregor, S. *Bun Cheung, Y., Cueto, S., Glewwe, P., Richter, L., Strupp, B. & the International Child Development Steering Group* (2007)Developmental Potential in the First 5 Years for Children in Developing Countries', *Lancet*, 369 (9555),pp. 65
[q] Barton, R. (2009) Acting: Onstage and Off, Fifth edition. Wadsworth, Cengage learning: Boston.
[r] Boal, A (2005). Games for actors and non-actors, second edition. Taylor and Francis online publication.

Lesson 8: Diverse Diets

Creating a balanced meal

Objectives:

By the end of this session, participants should:

- be able to make a balanced meal using what they have in the community
- Be able to observe that children observe parent's eating behaviours.

Materials needed:

- Drum [old pots or buckets and sticks can be used in the absence of a drum], two spoons, a piece of string (or bark or tape), pots, pans, plates, cups and ingredients for meal

> ***Remember:*** *Always keep a bucket of clean water, a cup, soap or ash, a clean piece of cotton chitenge and dish for hand washing.*

Introduction

Facilitator:
Always remember to:

- Greet the mothers and their children as they walk in.
- always be friendly and welcoming
- Encourage all to sit in circle, as you continue this "village meeting."
- Beat the drum to signal that the meeting has begun.
- Thank mothers for coming to today's session

> **NOTE**: *Always be welcoming, smile, be friendly and open (approachable). It is important to be welcoming so that participants can open up and feel free to express themselves, and also be able to approach you when they need help.*

The Mother Hen Activity [1a] Again!

Facilitator:

- Ask all to stand in two lines (queues) then face each other.
- Then tell them that the person they are facing is now their mother in this exercise. This mother must take care of her "young child" and make sure no harm comes to them, for instance bumping into someone, or a stone or tree or wall. The chick is supposed to follow the mother's voice and trust that the mother hen will keep her safe.

[1] This activity is originally known as Noises in Boal's Games for actors and Non-actors, second edition. However, I renamed it, after observing a hen and its chicks, and added to the rules to suit the goals of this course. In this case this activity proved very effective in explaining what a mother's role is in her child's life. The mothers and community health workers also felt it was a very relatable concept.

- Ask the mother hens, to make chicken sounds (clucking) and approach their chicks (who have to close their eyes), then begin to walk. Mother must make sure that their child is following, able to hear her clearly. The chick must follow the sounds of their mother hen.

- The mother hen make three sounds: a quiet, calm noise for the child to follow, one loud noise to warn of danger so that the chick can stop when faced with "danger"(please note that is no actual danger, do not cause harm or encourage it) and then a humming sound to act as a pretend feeding break.

- The goal is for the mother hen to guide, and protect their chicks without touching them with her hands. Do not force, guide.

- Once this is done (duration, 5-7 minutes), the facilitator can ask the following questions per pair:

Questions
How did you (the 'chick') feel as you were being led? How did you (the 'mother hen') feel as you led your chick? What did you fear? At what point where you really worried? What did you think or do, in that instance?

- And then the one who was the mother hen can now play the role of the young chick and the one who was the chick at first can now become the mother hen.

- Repeat the questions after the switch and exercise is done.

NOTE: *Remember that as a facilitator, you act as a guide through this process. You will decide when to start and when it's feeding time. You will also explain the game to other participants as it begins. However, perceived danger will be determined by the" mother hen" who will lead the chick. Only the chick closes its eyes, the mother hen needs her eyes to remain open so that she can see where they (mother and chick) are going.* ***If children are present the mother hen also takes responsibility for the children as well. She must encourage them to take part in the movements as well.***

Additional questions [ask after this exercise is done]: Did you learn anything from this exercise? Is this similar to the relationship between a mother and her child? If yes; why? If not, why? What is a parent's role in the child's development?

You have already looked at guide and protect, in this case you are now looking at the practical aspect of feeding.

Sing-Along Time!

Facilitator, say:

- "Does anyone know an action song that talks about healthy foods/ local foods? Or maybe a song that talks about the good outcomes of good care/ feeding?"

- If so, can *[insert name of person who assents]* teach it to the rest us? If not, let's make one up?

Always encourage use of local songs along with mother-child interaction.

Circle of knots exercise[2b]

Facilitator:

- Ask mothers to make a big circle (do not hold hands): stretch out arms until the fingertips are almost touching.
- Then tell mothers to make a small circle or a huddle until no space is left.
- Ask mothers to chant the ritual or group name once the circle is small.[3]
- Then make a big circle once more.

NOTE: *This activity helps boost the morale and reminds learners that these sessions have a positive goal. Once done, move on to the cooking demonstration.*

Let's COOK: Aatu jiike[4]

Please note: all present must wash their hands with soap or ash before you begin preparing the meal. Also wash the vegetables and/ or fruits thoroughly before cutting them.

---

[2] Only the first part of this activity, the elastic circle is used.

[3] It is important to allow participants to create a group name or ritual (activity or signal) with which they can identify with This should be done during the lesson or meeting. This should not be confused with the ritual activity (sound, gesture) in Boal, A (2005) Games for actors and non-actors..

[4] The menu template was downloaded from: http://www.nourishinteractive.com/nutrition-education-printables/category/16-printable-kids-healthy-menu-plans-daily-meal-planner-childrens-healthy-food-groups-balanced-meals

MENU OUTLOOK

[Removed for publication purposes]

TIME	FOOD & GROUP	INGREDIENTS	PREPARATION
**20 min.**	Mundyoli<br>(body building)	Pumpkin leaves(dry) & groundnuts (pounded), ½ onion	Boil water till hot, then add pumpkin leaves and groundnuts, until cooked, then add onion and salt… allow to simmer
**20 min.**	Nshima<br>(energy giving)	Roller meal* (best) & water	Boil water, pour mealiemeal and stir until ready
**7 min.**	Moringa<br>(protective)	2- 3 cups fresh Moringa, 1 tomato, 1 onion, 2 tablespoons of oil	Put oil on hot pan, add 1 tomato & ½ onion fry, and then add moringa leaves. Fry till ready.
	Fruits & drink (protective)	Mbula/Masau<br>Drink: cibwantu/ water	Wash fruits & serve in bowl<br>Cibwantu, will be ready made if available

* Roller meal is rich in protein, carbohydrates, fat and fibre. It also contains iron, zinc & B-vitamins (thiamine & folate)[c]

Let's EAT: Aatu Lye

Facilitator, say:

- *You have done this activity before at home.*
- *Now we are going to eat with our children, feeding them the way we have practised. It's your time.*

Responsive feeding exercise[5d]

Responsive Feeding Exercise

Materials: two spoons and tree bark or sack string

Facilitator, say:

- In lesson 2, 4 & 7 we learnt that responsive feeding helps in the prevention of stunting (especially with relation to underfeeding), develop language & teaches the child good feeding behaviours. The mother is also better able to monitor what her child eats and the amount.
- **Our task is to**: Talk to our child (or children) as we feed them.

[5] Suggested home activity in lesson 4.

- You can pretend that the spoon is an train trying to deliver goods (the food) into a special destination (the child's mouth). Be excited and happy.

- You can tie two spoons tightly together by their handles, so that when the child eats the mother can also use the other end to feed herself. *This will encourage the child to eat as mummy is doing it too. It will also help you be able to monitor what your child is eating, and how much.*

> **NOTE***: there will be no homework review in this session. Neither will any home tasks be given using the village of dreams. Notify mothers that they can practice the task they chose in lesson 7.*

Sharing Knowledge: 15 minutes of fame[6]

Note: this activity could be optional as it depends on the group's willingness to do so. However, it is a good tool for informing the community on what was done during the intervention or programme. It also gives the participants a sense of purpose.

Facilitator, say: Now, we have learnt, recalled and even applied some information relating to early child development, it is our duty to share this information just as the chief did. Therefore, let us take this time to plan a final performance to be shared with the community.

Good day exercise [the final act][e]

- Greet or hug each other and tell each other something nice for the day. Here, each mother hugs another mother and says positive things about the other. For instance, when Jane greets Mary, she will say, 'Hi my name is Mary and I am very kind.'
- Then Mary will return the favour, by saying her name is Jane and…....

> ***NOTE***: this allows mothers to leave the group with positive feelings about themselves and each other.

Have a lovely day…

[6] Refer to Michael, J., Michel, J. & Silverberg, J. (2001) Ready for action: A popular theatre/popular education manual, page 30 for guidelines on how to plan a participatory performance.

References

[a] Boal, A (2005) Games for actors and non-actors, second edition. Taylor and Francis online publication.

[b] Boal, A (2005) Games for actors and non-actors, second edition. Taylor and Francis online publication. [Only the first part of this activity was used as the second part was deemed too complex at this stage.]

[c] Roller meal. Retrieved from: http://www.umoyo.com

[d] Castle, J. (n.d.). Why you need to practice responsive feeding. Retrieved from: https://www.bundoo.com/articles/why-youneed-to-practice-responsive-feeding/

[e] Boal, A (2005). Games for actors and non-actors, second edition. Taylor and Francis online publication.

Lesson 9: Socio-emotional Development.

A family affair

Objectives:

By the end of this session, participants should:

- Recall the importance of taking care of oneself.
- Know that the home environment is critical for good socio-emotional development in the child.
- Know that maternal well-being will influence child's well being
- Be able to relate to others, self and child without being forceful.

Materials Needed:

- Drum [old pots or buckets and sticks can be used in the absence of a drum], a clay village (village of dreams), activity list and a dice.

> ***Remember*****:** *Always keep a bucket of clean water, a cup, soap or ash, a clean piece of cotton chitenge and dish for hand washing.*

Introduction

Facilitator:

Always remember to begin a session by:

- Greeting the participants and their children. Ask about their day, week, etc.
- Introduce yourself and briefly talk about what the meeting is about.
- Encouraging co-learners to sit in a circle, imitative of a village meeting. If possible the facilitator can have a drum[1] [old pots or buckets can serve as alternatives] present to begin the session.
- Thanking participants for attending this meeting and telling them you are happy to be meeting with them.

> **NOTE**: *Always be welcoming, smile, be friendly and open (approachable). It is important to be welcoming so that participants can open up and feel free to express themselves, and also be able to approach you when they need help.*

[1] A drum is an aesthetic of popular theatre as a part of this context's oral culture, I felt it would be an effective means for signaling to the participants that the session has began as well as getting everyone involved through sound and movement.

Face-Off: Pass the face into the trust circle

Facilitator:

- Ask all to stand, maintaining the circle, and do a stretch exercise: stretch arms upwards, sideways, back to front, touch your toes, bend your knees, jump and try to touch the sky (reach for the heavens) and move head and neck. Encourage mothers to encourage children to stretch as well!

Always: pair these with breathing exercises (make funny faces and sounds with each stretch).

Remember facial expressions[2] help teach the child how to express emotions.[a]

Pass the face[3b]

Then say:

- Now, we are going to play a game called pass the face. Here, one person at a time, going clockwise around the circle, will make a face. It can be of any emotion (happiness, sadness, anger, a frown, etc) or a silly face.
- The person to the immediate left of the face-maker must mimic that face; then make a new face to pass on to the mother on their left. Everyone should make at least one face. *[**Variations** centred on sound can be made instead of facial expressions, or both can be done.]*

The trust pillars[4]

- "Now close your eyes and move around carefully. Then I am going to shout out numbers (e.g. 1 or 2 or 3) and each one is supposed to find a partner (with eyes closed). Then I am going to tell you to open your eyes, to see if you've made it. Please, do not rush take your time."
- Let's all now be in threes, two people on each side (two facing each other and one in the middle_ can face any direction).
- The middle person must close eyes and the other two will push her from side to side (feet must not move from the centre). Then you can switch.
- Let's make a circle again; this circle must be very tight. One of us will go on the centre, and we will push person from one side to the next (feet must remain on the centre).

Q & A

Q: Why are we doing this?

A: The faces or sounds teach children affect (how to show emotion) and also teach one to communicate non-verbally with others. The blind walk, teaches us to sense others. While the trust pairs, teach us to trust and rely on each other, and that we must also support and be there for each other as well.

---

2 Facial expressions are an essential survival emotional skill, especially during the first years of life, through which the child is able to elicit the caregiver's help and attention. Children also have a predisposition to attend and respond to facial expressions from their primary caregivers, hence the mother's face serves as the child's learning interface in this case.

3 This game helps develop as well as enhance facial movement skills in acting. In this context it simply serves as an opportunity for the child to learn about different emotions and other facial expressions all important for social emotional growth.

4 This is a variation of the Joe egg. In this instance, I felt it could be used to put across the message of mothers needing a support system through family in raising the child.

Village of dreams: pass the ball activity[5]

Materials needed: a ball or soft cloth

Facilitator:

- Say: “this is an activity that we will do every week. Here, we want to reflect on what we have accomplished so far. This will encourage us to commit to the activities that we chose to do during the *‘village of dreams game’* with our children or by ourselves.”
- Ask all to sit in a circle pass the ball or a soft cloth
- Ask everyone to reflect on the “village of dreams” activity that we chose. Say: “Which activity did you commit to do by yourself or with your child last week? Did you do it? If yes, when is your turn to hold the ball arrives, you should say what activity you did and how. If not, you should still say the activity we were supposed to do and activity that we have done with our child.”
- Now let’s pass the ball.
- Then, talk about how the homework went. What was easy/difficult or fun/not fun? Discuss the challenges faced by the individuals as a group and then allow mothers to give suggestions on how to overcome these challenges before you propose new activities or alternative ways of doing them.
- Say: “One important thing to remember is that we can also do these activities as we work or do chores, especially if we do not have time to spare: for example you can talk to your child about the work at hand, ask child “to help” with chores (for instance, put this plate in that dish for me).”

Circle of knots exercise[6c]

Facilitator:

- Ask mothers to make a big circle (do not hold hands): stretch out arms until the fingertips are almost touching.
- Then tell mothers to make a small circle or a huddle until no space is left.
- Ask mothers to chant the ritual or group name once the circle is small.[7]
- Then make a big circle once more.

Q&A

Q: Why are we doing this exercise?

A: We are doing this so as to bring our focus onto the lesson at hand and remember that team spirit and effort are important.

5 [illegible] te a stress [illegible] proceeds, [illegible] ow in air)

6 [illegible]

7 It is important to allow participants to create a group name or ritual (activity or signal) with which they can identify with This should be done during the lesson or meeting. This should not be confused with the ritual activity (sound, gesture) in Boal, A (2005) Games for actors and non-actors..

Sing-Along Time!

Facilitator, say:

- "Does anyone know a song or game that talks about happiness? Or maybe a song that talks about how to live well with others?"
- If so, can *[insert name of person who assents]* teach it to the rest us? If not, let's make one up?

INFORMATION BANK: SOCIAL-EMOTIONAL DEVELOPMENT

Social emotional development

Social emotional development refers to the child's expressions and management of emotions and the ability to establish positive and rewarding relationships with others[d] in their day to day experience.

Importance of social emotional development

Social emotional development influences how competent the child is in that society as it determines the child's ability to identify and understand feelings, to correctly read and understand emotional states in others, to manage strong emotions and their expression in a constructive manner, to regulate one's own behaviour, to develop empathy for others, and to establish and maintain relationships.[e]

Infants experience, express, and perceive emotions before they fully understand them.[f] In learning to recognize, label, manage, and communicate their emotions and to perceive and attempt to understand the emotions of others, children build skills that connect them with family, peers, teachers, and the community.[g] These growing capacities help young children to become competent in negotiating increasingly complex social interactions, to participate effectively in relationships and group activities, and to reap the benefits of social support crucial to healthy human development and functioning.[h]

Healthy social-emotional development for infants and toddlers unfolds in an interpersonal context, namely that of positive ongoing relationships with familiar, nurturing adults.[i] Young children are particularly attuned to social and emotional stimulation. Even newborns appear to attend more to objects that resemble faces.[j] They also prefer their mothers' voices to the voices of other women.[k] Through nurturance, mothers support the infants' earliest experiences of emotion regulation.[l]

Factors influencing social emotional development

During the village of dreams exercise we encouraged each mother to think on what she wants her child to be when he or she grows up and also for the mother to have good thoughts towards herself. This is to encourage sensitivity (responsiveness) and warmth towards the child's needs. This is also because we have learnt that for healthy social emotional development to occur, ***a child needs a healthy environment (stimulatory and responsive parenting) and a healthy mother.*** *A healthy mother has good thoughts towards herself and her child, and is able to nourish and create a good environment for her child. She also uses good parenting practices to manage her child's behaviour; thus encouraging good behaviours in the child and discouraging bad ones. A mother must be sensitive and warm.*

We have already established that if a mother is not well. For instance, if she is sick or unhappy most of the time then she won't be able to respond to her child properly, leading to neglect or harshness. Therefore, it is important to ask for help in such situations.

Note: If you need help it is best to seek it as soon as possible. Talk to your friends, or elders or go to the hospital, if you are feeling unwell.

Parental warmth and sensitive responsiveness are important in creating a socially competent child.[m] For instance, answering nicely when your child calls, talking to your child or playing with your child enhance your child's social emotional development. This is not limited to the mother but other adults and older children who interact with the child especially regularly_ **family**.

Parenting Styles:

In lesson 1 we talked about how a mother's role is to guide. **Parenting styles are ways we use for guiding our children.** These refer to how we discipline or communicate information to our children.[n] They are important in building healthy social emotional development which leads to social competence in that child. There are four types:

Diagram not included for purposes of publication

Questions: Which of these do you think is best to encourage healthy social emotional development in your child? Why? *[Allow mothers to respond before giving the answer.]*

Answer: See the diagram below[o]

Diagram not included for purposes of publication

***NOTE**: Explain content the diagrams*

As shown, **the parenting style a mother uses determines the child's well being**. A mother therefore must be responsive, not harsh or permissive.

The mother's well being and competence

Now you know that you should take care of yourself and your child. What will you do to ensure that your child has healthy social emotional development? How best can we help each other be better parents to our children?

Remember, that how you raise your child is the way they will raise their children as well.

Games for Socio-emotional development

***Lengu-Lengu**[p]*

Facilitator, say:

- Let's all rise in a circle, all-bending forward patting on our thighs or clapping ours hand singing the song "Lengu- Lengu".
- This game proceeds with mothers 1 and 3 slightly peeping over participant number 2 and looking at each other. 2 and 4 then do the same, 3 and 5, 4 and 6, 5 and 7, etc (only two mothers stretch at once. While the rest remain bending while singing until their turn).
- If one of us fails to rise we start again.

Questions:

What do we learn from a game like Lengu?

NOTE: *This game is good for encouraging cooperation and alertness in that for the cycle to continue players have to be in tune with each other. Here we see both physical, cognitive and social development/ integration are encouraged."*

Lean on me (or count on me)[8q]

Facilitator, say:

- Let's all pair up (in twos) with our backs towards each other.
- We will start with leaning on each other while sitting, and then we will try to stand up. Don't touch the ground or use your hands, just your backs.
- You can bend forwards or backwards or sideways and go up or downwards as long as you don't lose contact.
- Now, let's face each other, seated on the floor, legs apart and slightly stretched, knees bent, soles of the feet flat on the floor and close to your partner's feet; mothers take one another by the arms (not just by the hands, which is much more difficult) and brace each other feet to feet.
- First one partner rises, pulled by the other, and then, as she goes down, the second one begins to rise, in such a way that at a given moment, both will be halfway up – just like two children playing on a real see-saw.

Q & A

Q: Why are we doing this?
A: This exercise is about using all one's strength not to win but to support each other. Mothers, not only are we the support system for our children but we are each other's support system. Let's help each other, listen to each other and encourage each other as we try to raise our children well. Remember, how we behave towards each other is how our children will behave towards others.

General Homework

Facilitator, say:

- **Let's all think about whom we want our children to be and how we think we can help them achieve this.**
- **Questions to ask yourself include: Who is my child? What plans do I have for my child?**
- **We can share this information when we meet next time**

Sharing Knowledge: 15 minutes of fame[9]

[8] This activity is made out of a series of pushing against each other exercises from Boal's arsenal of theatre of the oppressed known as 'back to back and the see-saw.

Note: this activity could be optional as it depends on the group's willingness to do so. However, it is a good tool for informing the community on what was done during the intervention or programme. It also gives the participants a sense of purpose.

Facilitator, say:

-Now, we have learnt, recalled and even applied some information relating to early child development, it is our duty to share this information just as the chief did. Therefore, let us take this time to plan a final performance to be shared with the community.

Good day exercise [the final act][r]

- Greet or hug each other and tell each other something nice for the day. Here, each mother hugs another mother and says positive things about the other. For instance, when Jane greets Mary, she will say, 'Hi my name is Mary and I am very kind.'
- Then May will return the favour, by saying her name is Jane and…....

***Note**: this allows mothers to leave group with positive feelings about themselves and each other.*

Have a lovely day....

Love your family

References

[9] Refer to Michael, J., Michel, J. & Silverberg, J. (2001) Ready for action: A popular theatre/popular education manual, page 30 for guidelines on how to plan a participatory performance.

[a] Izard, E. C., Youngstrom, A. E., Fine, E. S., Mostow, J. A., & Trentacosta, J. C. (2006) Emotions and the development of psychopathology in infancy and early childhood. In D. Cichetti and D. J. Cohen (Eds.), *Developmental psychopathology: Theory and method, second edition (Pp 254- 59)*. John Wiley & Sons: New Jersey.

[b] Michael, J., Michel, J. & Silverberg, J. (2001) Ready for action: A popular theatre/popular education manual. Retrieved from: http://wwwwpirg.org

[c] Boal, A (2005) Games for actors and non-actors, second edition. Taylor and Francis online publication. [Only the first part of this activity was used as the second part was deemed too complex at this stage.]

[d] Cohen, J., and others. (2005) *Helping Young Children Succeed: Strategies to Promote Early Childhood Social and Emotional Development*. Washington, DC: National Conference of State Legislatures and Zero to Three. As cited on the California Department of Education article Social-Emotional Development Domain: California Infant/Toddler Learning & Development Foundations. Retrieved from: http://www.cde.ca.gov/sp/cd/re/itf09socemodev.asp

[e] National Scientific Council on the Developing Child (2004, p. 2) As cited on the California Department of Education article Social-Emotional Development Domain: California Infant/Toddler Learning & Development Foundations. Retrieved from: http://www.cde.ca.gov/sp/cd/re/itf09socemodev.asp

[f] California Department of Education article Social-Emotional Development Domain: California Infant/Toddler Learning & Development Foundations. Retrieved from: http://www.cde.ca.gov/sp/cd/re/itf09socemodev.asp

[g] California Department of Education article Social-Emotional Development Domain: California Infant/Toddler Learning & Development Foundations. Retrieved from: http://www.cde.ca.gov/sp/cd/re/itf09socemodev.asp

[h] California Department of Education article Social-Emotional Development Domain: California Infant/Toddler Learning & Development Foundations. Retrieved from: http://www.cde.ca.gov/sp/cd/re/itf09socemodev.asp

[i] Johnson, M., and others (1991). "Newborns' preferential tracking of face-like stimuli and its subsequent decline," *Cognition,* Vol. 40, Nos. 1-2, 1–19. As cited on the California Department of Education article Social-Emotional Development Domain: California Infant/Toddler Learning & Development Foundations. Retrieved from: http://www.cde.ca.gov/sp/cd/re/itf09socemodev.asp

[j] Johnson, M., and others (1991). "Newborns' preferential tracking of face-like stimuli and its subsequent decline," *Cognition,* Vol. 40, Nos. 1-2, 1–19. as cited on the California Department of Education article Social-Emotional Development Domain: California Infant/Toddler Learning & Development Foundations. Retrieved from: http://www.cde.ca.gov/sp/cd/re/itf09socemodev.asp

[k] DeCasper, A. J. & Fifer, P. W. (1980) Of human bonding: Newborns prefer their mothers' voices, *Science,* 208 (6), pp. 1174–76. as cited on the California Department of Education article Social-Emotional Development Domain: California Infant/Toddler Learning & Development Foundations. Retrieved from: http://www.cde.ca.gov/sp/cd/re/itf09socemodev.asp

[l] Thompson, R. A., & Goodvin, R. (2005) "The Individual Child: Temperament, Emotion, Self and Personality," in *Developmental Science: An Advanced Textbook* (Fifth edition). Edited by M. H. Bornstein and M. E. Lamb. Mahwah, NJ: Lawrence Erlbaum Associates.as cited on the California Department of Education article Social-Emotional Development Domain: California Infant/Toddler Learning & Development Foundations. Retrieved from: http://www.cde.ca.gov/sp/cd/re/itf09socemodev.asp

[m] Cassidy, J. & Shaver, R. P. (2008) Handbook of Attachment: theory, research and practical applications, 2nd edition. Guilford Press: New York.

[n] Hapunda, G. (2015). Parenting styles and strategies. Lecture notes pdf presentation.

[o] Hapunda, G. (2015). Parenting styles and strategies. Lecture notes pdf presentation.

[p] Sport In Action (2004) Sport in the development process: Leadership manual. SIA.

[q] Boal, A (2005). Games for actors and non-actors, second edition. Taylor and Francis online publication.

[r] Boal, A (2005). Games for actors and non-actors, second edition. Taylor and Francis online publication.

Lesson 10: Social emotional Development

Raising the healthy child

Objectives:

By the end of this session, participants should:

- Recall that a mother must play with their child
- Recall that each child is unique
- Know that a good relationship with the mother must exist for child to grow well.

Materials Needed:

- Drum [old pots or buckets and sticks can be used in the absence of a drum]

***Remember**: Always keep a bucket of clean water, a cup, soap or ash, a clean piece of cotton chitenge and dish for hand washing.*

Introduction

Facitator:
Always remember to:

- Greet the participants and their children. Ask about their day, week, etc.
- Introduce yourself and briefly talk about what the meeting is about.
- Encourage co-learners to sit in a circle, imitative of a village meeting. If possible the facilitator can have a drum[1] [old pots or buckets can serve as alternatives] present to begin the session.
- Thank participants for attending this meeting and telling them you are happy to be meeting with them.

NOTE: *Always be welcoming, smile, be friendly and open (approachable). It is important to be welcoming so that participants can open up and feel free to express themselves, and also be able to approach you when they need help.*

[1] A drum is an aesthetic of popular theatre as a part of this context's oral culture, I felt it would be an effective means for signaling to the participants that the session has began as well as getting everyone involved through sound and movement.

GAME: Recognize the Aaah!

Facilitator:

- Ask all to stand, maintaining the circle, and do a stretch exercise: stretch arms upwards, sideways, back to front, touch your toes, bend your knees, jump and try to touch the sky (reach for the heavens) and move head and neck. Encourage mothers to encourage children to stretch as well!

Recognize the Aaah[2a]

Facilitator, say:

- Let's all close our eyes, and start walking around through the room.
- As we do so, I am going to touch one of you on the shoulder and who ever I touch must vocalise a sigh: 'aaah!'
- Then the rest of us must identify who uttered the 'aaah!'

- We will continue until all of us have been touched and been identified.

> **Q & A**
>
> ***Q***: Why are we doing this?
> ***A***: this exercise shows that the way we now know each other, is the way our children should know us. Our eyes were closed but we were able to tell who some (or all) of were.

Village of dreams: pass the ball activity[3]

Materials needed: a ball or soft cloth

Facilitator:

- Say: "this is an activity that we will do every week. Here, we want to reflect on what we have accomplished so far. This will encourage us to commit to the activities that we chose to do during the *'village of dreams game'* with our children or by ourselves."
- Ask all to sit in a circle pass the ball or a soft cloth
- Ask everyone to reflect on the "village of dreams" activity that we chose. Say: "Which activity did you commit to do by yourself or with your child last week? Did you do it? If yes, when is your turn to hold the ball arrives, you should say what activity you did and how. If not, you should still say the activity we were supposed to do and activity that we have done with our child."

[2] This game was easy to apply in this context [as the target focus was only mothers. Please note that in a multiple gender group, the blind series handshake would be optimal. Also take into consideration religious beliefs before attempting activities that require body contact.

[3]The passing game from Barton's (2009) Acting: Onstage and off book, serves as the base for this task. I created this activity so as to create a stress free environment for reflection on homework tasks. As the mother passes the ball and responds to facilitator's questions and the discussion proceeds, the mother is disarmed and appears less likely to worry about reflection activity at hand. She is free to play with the ball (e.g. squeeze or throw in air) before passing it on.

- Now let's pass the ball.
- Then, talk about how the homework went. What was easy/difficult or fun/not fun? Discuss the challenges faced by the individuals as a group and then allow mothers to give suggestions on how to overcome these challenges before you propose new activities or alternative ways of doing them.
- Say: "One important thing to remember is that we can also do these activities as we work or do chores, especially if we do not have time to spare: for example you can talk to your child about the work at hand, ask child "to help" with chores (for instance, put this plate in that dish for me)."

Circle of knots exercise[4b]

Facilitator:

- Ask mothers to make a big circle (do not hold hands): stretch out arms until the fingertips are almost touching.
- Then tell mothers to make a small circle or a huddle until no space is left.
- Ask mothers to chant the ritual or group name once the circle is small.[5]
- Then make a big circle once more.

Q&A

Q: Why are we doing this exercise?
A: We are doing this so as to bring our focus onto the lesson at hand and remember that team spirit and effort are important.

Sing-Along Time!

Facilitator, say:

- "Does anyone know a song or game that talks about happiness? Or maybe a song that talks about how to live well with others?"
- If so, can *[insert name of person who assents]* teach it to the rest us? If not, let's make one up?

[4] Only the first part of this activity, the elastic circle is used.

[5] It is important to allow participants to create a group name or ritual (activity or signal) with which they can identify with This should be done during the lesson or meeting. This should not be confused with the ritual activity (sound, gesture) in Boal, A (2005) Games for actors and non-actors..

A Twist in the Tale[6]

> ***NOTE**: read this before hand and tell it as a story.*

After the search for knowledge came to an end, the chief called for a village meeting where the emissaries would share what they had found out in front of the whole village. Some emissaries confirmed that what the chief had dreamt was true. They talked about how the villages that considered children had very wise and educated people in them. They said that these villages were well developed and there no were no unnecessary illnesses or starvation, so all were healthy.

However, a few emissaries denied this claim. They said, "Oh, great and might chief, we have searched the entire world and we have seen that paying attention to children's well being is not worthwhile. It is a total waste of time; children are not the key to a great future. For greatness, we must increase the amount of time we spend planting and harvesting, have more children and wives so they can spend all day in the fields and we will be rich." And now there was confusion in the village....

Then one of the wise women rose up and said: "Wise one, Oh Chief of our hearts let us call the mothers in the groups in those villages were children were considered to matter. Let them come and show and tell us firsthand the real truth before we come to a decision."

Facilitator, say:

- "Now a letter has arrived, and we are part of the team that has been invited to share this knowledge. So now what are we going to do to show/ tell them what we know?
- Remember whatever, we do or say will determine what that village will do...
- We have decided to do a play, to show how life was before we made children a priority and after.

A play of sorts[7]

Facilitator, say:

- We are now going to make a play about an unhealthy and badly treated child. In this skit, let us include all that we know can result in the poor growth of the child. When the play ends, as we have done other times, anyone can point out what was wrong and it can be acted again, but this time with the correction.

[6] This is a fun way to get learners to recall everything they have learnt and confidently share without feeling like they are being tested.

[7] Forum theatre

-

The Interview[8]

Main characters: the interviewer, mothers [Luano versus Luyando's parents] and the unbelieving messengers [doubting Thomas]

- Then we are going to pick the two (or more) mothers, who acted as Luano and Luyando's mothers [also known as the "bad" and "good" mother] and interview them in their characters. Remember, the stop, walk, justify game. This is almost the same. Only here, the actors will have to explain why they did what they did in the play.
- After this phase let Luano's mother go, and continue with Luyando's mother.
- Now as Luyando's mother explains their reasons, the **unbelieving emissaries** can say: "I don't believe you."

Questions[to ask the 'actors']:

Is what happened true? How did you know what to do? What benefits are there for the children now? What proof do you have? Is that so? So what would be your advice?

NOTE: *the goal is not to attack individuals. No bad comments, only allowed the one phrase.*

- The core idea here is to provide a convincing case for early childhood care services or theme of choice.
- Make sure no personal attacks arise; it is your goal to maintain peace at all times. Only constructive arguments are allowed and all, including emissaries must stay in character until the interview or discussion is done.
- If this becomes too tense, you can pause the discussion. Use the ritual as a morale booster, stretch a bit; and then get back to the discussion.

INFORMATION BANK: SOCIAL COMPETENCE

A focus on social emotional development

Social and emotional skills are important for good mental health and wellbeing, learning, motivation to achieve and cooperate, and the development of values of the child;[c] they also have an impact child's self-concept and wellbeing throughout life.[d] In the previous lesson, we learnt that the parent is the one who creates the environment for the child that will in turn influence his or her behaviours. Therefore, a mother must do her best to be healthy (if possible); 'she must also act as a secure base

[8] This interview is an element of Theatre for Development (TfD) called hot seating used to get the core message across to target audience at a more in-depth level. The characters are to give a convincing argument for what they chose to do. This context also creates a safe monitored environment for discussion which might otherwise been harmful in the real context. It is very important that the facilitator be well trained in the running of a forum before this can be attempted.

and a safe haven for her child.[e] This means that the child must know that mommy will protect me in case of danger and that mom will not hurt me because she loves me.[f] Only through this, circle of security can healthy social emotional development resulting in social competence.[g]

Discussion: What is social competence?[9]

Response: this is one's ability to live well with others in their community.

Who is the socially competent child?[10]

When asked some of you said, this a child who doesn't fight with others, who listens to what adults say (obedience), who shares with others, who respects adults and friends, who is responsible and so on. This of course is all very true.

A socially competent child will show empathy, be able to know when to express specific emotions, interact with other adults and peers, and be kind to others and able to share and so on.

Creating social competence

Our children will show individual differences in temperament, development and personal preferences. Some will be outgoing and sociable.[h] Some will prefer to watch before joining in. Some will be highly independent. It is important to look for and respect these individual differences, while supporting the development of a range of skills in each child. Avoid comparing them. For example, saying: "why can't you be more like your bother?"

To support children's development of social and emotional skills and a positive self-concept:[i]

- ✓ Observe children's verbal and non-verbal signals and get to know them as individuals.
- ✓ Respond in a caring and consistent way to children's physical and emotional needs.
- ✓ Keep expectations appropriate to the child's abilities and stage of development.
- ✓ Provide opportunities for alone time or quiet activity and for play with peers or adults.
- ✓ Give children choices and opportunities for exploration, to learn about their environment and interact with others.
- ✓ Respect and value the personality and individual preferences.
- ✓ Respect and support children's early attempts at connection and communication.
- ✓ Consistently model the behaviour and communication styles you want your children to use.
- ✓ Acknowledge children's achievements and give praise for positive behaviour.
- ✓ Help children to recognise and label their emotions and to express their feelings.
- ✓ Use stories, games or other activities to explore feelings and friendships.
- ✓ Encourage older children to take turns, to share resources and to share adults' attention; and care or play with their younger siblings.
- ✓ Model pro-social skills and praise children for showing empathy and helping others.
- ✓ Help children to solve problems and negotiate with peers when disputes come up.

[9] This definition is based on the mother's responses during cooperative inquiry.

[10] This description was derived through mother's responses during a cooperative inquiry session.

- ✓ Be aware that children may have difficulty using their skills when they are sick or tired; this may explain their being difficult sometimes therefore show patience and be calm.
- ✓ Recognise that learning new skills requires time, practice and positive feedback.
- ✓ Discipline through guidance; explaining why what they have done is wrong or not good.
- ✓ Discipline (or reprimand) should also take place immediately after deliberate mischief has taken place so as not to confuse the child as to what is wrong.

The Mother-child Relationship[j]

When a child's signals are responded to by an adult within their social interactions and relationships in a reliable, predictable and meaningful way over time, the child and adult become 'in tune' with each other. Being 'in tune' with an adult helps the child to regulate emotions and learn what it is like to be calm. Over time the child becomes skilled at regulating their own emotions, a major developmental task of early childhood. Therefore talk to your child (remember the responsive feeding and makani a ajilo exercises) and play with your child. Guide them, without being too permissive or harsh (or controlling).

Questions:

Is this information useful to us? Are there any additions, to what has been discussed or said? Or what do we think or feel does not apply to us?

Catch me if you can: Sheep-sheep[k]

Facilitator, say:

- Let's draw two lines on the ground. Now one group of mothers (sheep) are going to be on one side of the line and others (lions) on the opposite line (lines should be about 6-10m apart).
- Then the lions, say: sheep-sheep come
    - Sheep: we are afraid,
    - Lion: what are you afraid of?
    - Sheep: we are afraid of the lions.
    - Lions: the lions are not here.
- Then the lions and sheep run towards each other. The lions' goal is to catch the sheep, while the sheep must avoid being caught until they reach the home line (formally the lion's home) on the other end.

NOTE: *This game helps with critical thinking and developing problem solving skills. It has an underlying message of ability to solve problems in the face of obstacles. During this game don't forget to include the children as with all other activities in this course. Also encourage participants to make roaring or baa sounds as they play.*

One last roll: the village of dreams

Materials: a clay dice and three huts

Facilitator:

- Give the mother a dice, and ask her to throw it. Once it falls, whichever side it falls on represents a hut (and type of task).
- Each hut has a specific task representation, that is: 1) hut 1_ blue door or plastic, is the hut of wishes, hopes dreams the mother has for her child; 2) hut 2_ brown door or plastic, is the hut of activities that mother will do with the child at home to stimulate good development, even with a busy schedule, and 3) hut 3_ green door or plastic, is the hut where the mother is asked to do something for herself that will help her be more relaxed and healthy.
- For huts 1 and 2, the tasks are highly subjective in that the mother bears all control on whether the task will be done or not. There is also no exact way of knowing if it was done or not. However, hut 2 tasks are more objective and it is easier to see if it was done. *Always note, that for hut 2_ what mother can do with the child, refer to the activity list*.

Sharing Knowledge: 15 minutes of fame[11]

Note: this activity could be optional as it depends on the group's willingness to do so. However, it is a good tool for informing the community on what was done during the intervention or programme. It also gives the participants a sense of purpose.

Facilitator, say:

-Now, we have learnt, recalled and even applied some information relating to early child development, it is our duty to share this information just as the chief did. Therefore, let us take this time to plan a final performance to be shared with the community.

Good day exercise [the final act][1]

- Greet or hug each other and tell each other something nice for the day. Here, each mother hugs another mother and says positive things about the other. For instance, when Jane greets Mary, she will say, 'Hi my name is Mary and I am very kind.'
- Then May will return the favour, by saying her name is Jane and…....

Note: this allows mothers to leave group with positive feelings about themselves and each other.

Have a lovely season.

[11] Refer to Michael, J., Michel, J. & Silverberg, J. (2001) Ready for action: A popular theatre/popular education manual, page 30 for guidelines on how to plan a participatory performance.

[a] Boal, A (2005) Games for actors and non-actors, second edition. Taylor & Francis online publication.
[b] Boal, A (2005) Games for actors and non-actors, second edition. Taylor and Francis online publication. [Only the first part of this activity was used as the second part was deemed too complex at this stage.]
[c] Kids-Matter Early Childhood: A framework for improving children's mental health and wellbeing.
[d] Lucich, M. (2002) Building baby's intelligence: Why infant stimulation is so important. Retrieved from: http://www.responseability.org/__data/assets/pdf_file/0008/4859/Building-
[e] Cassidy, J. & Shaver, R. P. (2008) Handbook of Attachment: theory, research and practical applications, 2nd edition. Guilford Press: New York.
[f] Cassidy, J. & Shaver, R. P. (2008) Handbook of Attachment: theory, research and practical applications, 2nd edition. Guilford Press: New York.
[g] The circle of security. Retrieved from: http://www.brainwave.org.nz/wp-content/uploads/2012/05/Circle-of-security-article
[h]Lucich, M. (2002) Building baby's intelligence: Why infant stimulation is so important. Retrieved from: http://www.responseability.org/__data/assets/pdf_file/0008/4859/Building-
[i] Lucich, M. (2002) Building baby's intelligence: Why infant stimulation is so important. Retrieved from: http://www.responseability.org/__data/assets/pdf_file/0008/4859/Building-
[j] Kids-Matter Early Childhood: A framework for improving children's mental health and wellbeing. Retrieved from: https://www.kidsmatter.edu.au/
[k] Sport In Action (2004) Sport in the development process: Leadership manual. SIA.
[l] Boal, A (2005). Games for actors and non-actors, second edition. Taylor and Francis online publication.

Additional Lessons

Lesson 11: Review of previous lessons

Lesson 12: An acting class

Lesson 11: Into the looking glass

What have we learnt so far?

Objectives:

By the end of this session, participants should:

- Recap most of what they learnt during the course of these lessons.
- Be able to clarify content learnt in previous lessons.

Materials Needed:

- Drum [old pots or buckets and sticks can be used in the absence of a drum]

> ***Remember*****:** *Always keep a bucket of clean water, a cup, soap or ash, a clean piece of cotton chitenge and dish for hand washing.*

Introduction

Facilitator:

Always remember to:

- Greet the participants and their children. Ask about their day, week, etc.
- Introduce yourself and briefly talk about what the meeting is about.
- Encourage co-learners to sit in a circle, imitative of a village meeting. If possible the facilitator can have a drum[1] (old pots or buckets can serve as alternatives) present to begin the session.
- Thank participants for attending this meeting and telling them you are happy to be meeting with them.

> **NOTE**: *Always be welcoming, smile, be friendly and open (approachable). It is important to be welcoming so that participants can open up and feel free to express themselves, and also be able to approach you when they need help.*

[1] A drum is an aesthetic of popular theatre as a part of this context's oral culture; I felt it would be an effective means for signaling to the participants that the session has began as well as getting everyone involved through sound and movement.

Relaxation and trust building exercises

Facilitator:

- Ask all to stand, maintaining a circle, and do a stretch exercise.[2] For instance, stretch arms upwards, sideways, back to front, touch your toes, bend your knees, jump and try to touch the sky (reach for the heavens) and move head and neck.
- Encourage mothers to encourage children to stretch as well.

Always: pair these with breathing exercises (make funny faces and sounds with each stretch).

Facial expressions[3] help teach the child how to express emotions.[a]

> ***Remember**: We are doing this to relieve tension and relax so that the mother is to be stress free (in a good, balanced mood/ state) so that she can pay better attention to her child.*

Sing-Along Time!

Facilitator, say:

- Does anyone know a song that talks about good care? Or one that talks about the
- If so, can *[insert name of person who assents]* teach it to the rest us? If not, let's make one up? We can also make one that talks about all the lessons we've had. Don't forget the actions!!!

  Always encourage use of local songs.

Circle of knots exercise[b]

Facilitator:

- Ask mothers to make a big circle (do not hold hands): stretch out arms until the fingertips are almost touching.
- Then tell mothers to make a small circle or a huddle until no space is left.
- Ask mothers to chant the ritual or group name once the circle is small.[4]
- Then make a big circle once more.

---

[2] This exercise was inspired by notes in Boal, A. (2005) Games for actors and non-actors and Anderson, J., Michel, J & Silverberg, J (2001) Ready for action: A popular theatre/popular education manual, encouraging a warm up before a lesson begins so as ready the learners for learning.

[3] Facial expressions are an essential survival emotional skill, especially during the first years of life, through which the child is able to elicit the caregiver's help and attention. Children also have a predisposition to attend and respond to facial expressions from their primary caregivers, hence the mother's face serves as the child's learning interface in this case.

[4] It is important to allow participants to create a group name or ritual (activity or signal) with which they can identify with. This should be done during the lesson or meeting. It should not be confused with the ritual activity (sound, gesture) in Boal, A (2005) Games for actors and non-actors.

Q&A

Q: Why are we doing this exercise?

A: We are doing it to relieve tension, to relax because a mother is supposed to be stress free (be in a good balanced mood) so that she can pay better attention to her child. Don't worry, be happy!

NOTE: *This activity helps boost the morale and reminds learners that these sessions have a positive goal. You can also use this exercise any time the morale is low or when learners seem tired or attention seems to be wandering to bring their focus back to the lesson at hand.*

Village of dreams: pass the ball activity[5]

Materials needed: a ball or soft cloth

Facilitator:

- Say: "this is an activity that we will do every week. Here, we want to reflect on what we have accomplished so far. This will encourage us to commit to the activities that we chose to do during the *'village of dreams game'* with our children or by ourselves."
- Ask all to sit in a circle pass the ball or a soft cloth.
- Ask everyone to reflect on the "village of dreams" activity that we chose. Say: "Which activity did you commit to do by yourself or with your child last week? Did you do it? If yes, when is your turn to hold the ball arrives, you should say what activity you did and how. If not, you should still say the activity we were supposed to do and activity that we have done with our child."
- Now let's pass the ball.
- Then, talk about how the homework went. What was easy/difficult or fun/not fun? Discuss the challenges faced by the individuals as a group and then allow mothers to give suggestions on how to overcome these challenges before you propose new activities or alternative ways of doing them.
- Say: "One important thing to remember is that we can also do these activities as we work or do chores, especially if we do not have time to spare: for example you can

[5]The passing game from Barton's (2009) Acting: Onstage and off book, serves as the base for this task. I created this activity so as to create a stress free environment for reflection on homework tasks. As the mother passes the ball and responds to facilitator's questions and the discussion proceeds, the mother is disarmed and appears less likely to worry about reflection activity at hand. She is free to play with the ball (e.g. squeeze or throw in air) before passing it on.

talk to your child about the work at hand, ask child “to help” with chores (for instance, stir this cibwantu for me).

Games: Agree/ Disagree[6]

Facilitator:

- Draw a line in the sand. Then read out the statements: If mothers agree, they should run to the right side of the line, if they disagree, they should run to left side.

Statements for Agree or Disagree game

a. **Being stunted or being a dwarf is the same thing**

   Answer: Disagree

   Being stunted is as a result of lack of a balanced healthy diet, while dwarfism has to do with the way a person was born (or is naturally).

b. **Talking to our children has no value; it is a waste of time.**

   Answer: Disagree

   Talking to our children is important. Conversations with the child help the child's emerging self concept and their understanding of self and others. It also helps them structure their thoughts (memory narratives) in a culturally appropriate way.

c. **We should always find time to play with our children.**

   Answer: Agree

   Playing with our children helps stimulate their growth in all four areas: cognitive, motor (physical), language and socio-emotional development.

d. **Learning, in children only begins when they reach grade one (at five or six years plus).**

   Answer: Disagree

   Children begin learning much earlier than that. Although it is important to make sure that what is being taught and how is age appropriate. E.g.: you can't teach a two year old how to cook nshima but you can talk about how you are cooking the nshima)

e. **There are many ways of purifying water.**

   Answer: Agree

   Boiling, chlorination, sieving, etc

f. **How we treat our children, influences how they behave and how they treat others as well.**

[6] Structure for the agree/ disagree game is gotten from the previous curriculum used in phase I of the Saving brains study; although the content varies.

Answer: Agree

Children learn from us (parents, siblings, grandparents and other adults they are in close contact with).

A throw down memory lane [20 min max]

Facilitator, say:

- For the past few months we have been learning about ways in which we can improve our children's well being. We are going to use the ball of remembrance[7c].
- Now let's take the time to say what we remember from our lessons. Feel free to ask or make corrections, etc.

> **NOTE:** ***use the guidelines for the ball of rembrance in lesson1.***
>
> ***Questions:***
>
> - Why are we focusing on children at this age (2yrs)?
> - How many areas of development have we looked at? Name them.
> - What do you remember about:
>   - Stunting? What causes stunting, in our children?
>   - A child's thinking (cognitive development)?
>   - Language development?
>   - Water, Sanitation and Hygiene?
>   - Child nutrition (feeding times, types of foods)?
>   - What did you prepare for the cooking lesson?
>   - Socio-emotional development (a mother and child's wellbeing)?
> - Which lesson was your favourite? What did you like about it?
> - Given a chance to do these mother groups again, what would you change?

[7] This activity is named for the passing technique used in acting during character development or learning script (Barton, 2009). In this case, I felt it would work well in gauging how much the participants knew on child development.

True or False: A tale of sorts [25 min. max]

> ***NOTE***: *select two volunteers to act as story tellers. Give them 5 minutes to create the stories, 5 minutes to tell or show them and 15 minutes to discuss these stories.*

Facilitator, say:

- So, we all know that our great chief sent emissaries to investigate issues relating to child development. Let
- Now, we are going to sit quietly and listen to two stories: either talking about what a mother can do to enhance a child's growth or the bad traits that lead to poor child growth/health.
- After the stories are done, we will have to decide which story is **true** and which one is **false.**
- Now, let's discuss the parts or elements of these stories that makes them true or false.

> ***Questions***:
>
> Why do you think this story is false? What are the true actions that lead to that end?

Health versus Illness: Keeping healthy[8de]

> **NOTE**: *this game is good for uncovering underlying issues, making it ideal when coming towards the end of a learning programme.*

Facilitator, say:

- Let's all walk around and try to use up all the space around us. We have to keep moving during the whole exercise.
- Each person should choose someone in the room, without letting that person know. This person is your illness and so you should try to keep as far away from them as possible.
- then choose another person, who will be your shield (health) and so you should try to keep them between yourself and the illness.
- After a short time, I'll shout 'FREEZE!'
- look around and to see if you are protected from your illness (bomb) by your health (shield).

[8] This game is adapted from the 'One person we fear, one person is our protector' exercise (Boal, 2005) or the bomb and shield exercise (Babbage, 2004). I felt it was the ideal activity to assert of the overall theme was heard.

Questions

How did this exercise make you feel? How do you think it relates to taking care of ourselves and our children? Was there any time you could not keep away from the illness? Etc

HOMEWORK FOR ALL

Facilitator, say:

- **This is our last lesson, for now. However, it does not mean we should stop doing the activities aimed at your child's development. After all, we are doing this for the sake of our children. Remember, they are the key to the future.**

Sharing Knowledge: 15 minutes of fame[9]

Facilitator, say:

- Let's take this time to continue planning what we will do for the final performance. What have we done so far?

Pass the Pulse[10f]

Facilitator, say:

- Let's all hold hands in a circle.
- On my signal, a volunteer will start passing the pulse by squeezing the hand of their neighbour on their right. This person then squeezes the hand of the person on their right. This carries on until the pulse returns to the person who started it.
- Make a signal that the circle is complete with a "shout" or "squeak" for example.
- Meanwhile, I will check how long it takes for the pulse to pass round the circle. Then we will do it again_ this time faster. Each round will be faster than the last one.

NOTE: *this activity is aimed at emphasizing what the participants are required to do after learning and practising what has been taught, which is to pass on this information. Feel free to use the 'Good day' exercise as well or replace the' pulse' with it if you feel it may not be as effective in your group.*

Be Happy! Stay energized

[9] Refer to Michael, J., Michel, J. & Silverberg, J. (2001) Ready for action: A popular theatre/popular education manual, page 30 for guidelines on how to plan a participatory performance.

[10]Adapted from the pass the tap & pass the clap activities (Michael, J., Michel, J. & Silverberg, J, 2001) and the movement comes back exercise (Boal, 2005).

[a] Izard, E. C., Youngstrom, A. E., Fine, E. S., Mostow, J. A., & Trentacosta, J. C. (2006) Emotions and the development of psychopathology in infancy and early childhood. In D. Cichetti and D. J. Cohen (Eds.), *Developmental psychopathology: Theory and method, second edition (Pp 254- 59)*. John Wiley & Sons: New Jersey.
[b] Boal, A (2005) Games for actors and non-actors, second edition. Taylor & Francis online publication.
[c] Barton, R. (2009) Acting: Onstage and Off, Fifth edition. Wadsworth, Cengage learning: Boston.
[d] Boal, A. (2005) Games for actors and non-actors, second edition. Taylor & Francis online publication.
[e] Babbage, F. (2004) Routledge performance practitioners: Augusto Boal. Routledge Taylor & Francis group: London.
[f] Michael, J., Michel, J. & Silverberg, J. (2001) Ready for action: A popular theatre/popular education manual. Retrieved from: http://wwwwpirg.org

Lesson 12: Sharing knowledge

A performance for all ages

Objectives:

By the end of this session, participants should:

- Be able to create a well rounded play for the final performance.
- Be able to improvise during performances
- Be able to share what was learnt in the classes with a larger group (their communities).

Materials Needed:

- Drum [old pots or buckets and sticks can be used in the absence of a drum] and a ball of string.

> ***Remember**: Always keep a bucket of clean water, a cup, soap or ash, a clean piece of cotton chitenge and dish for hand washing.*

Introduction

Facilitator:

Always remember to:

- greet the mothers and their children as they walk in.
- always be friendly and welcoming
- encourage all to sit in circle, as you continue this "village meeting."
- beat the drum[1] (old pots or buckets can serve as alternatives) to signal that the meeting has begun.

Trust Circle Mirrors[2a] into the Mad Chicken[b]

Facilitator:

- Ask all to stand, maintaining the circle, and do a stretch exercise: stretch arms upwards, sideways, back to front, touch your toes, bend your knees, jump and try to

[1] A drum is an aesthetic of popular theatre as a part of this context's oral culture; I felt it would be an effective means for signaling to the participants that the session has began as well as getting everyone involved through sound and movement.

[2] Known as the Colombian hypnosis which is a very good exercise through which to introduce ideas of responsibility while simultaneously providing a gentle yet thorough physical warm up.

touch the sky (reach for the heavens) and move head and neck. Encourage mothers to encourage children to stretch as well!

- One by one, have mothers choose someone across the circle to watch. This can be done by having the first person point to someone and then put their hand on their head to show they have been taken. The person pointed to does the same thing until everyone has pointed and been pointed to once.
- Check the pattern by having everyone point to the person they are watching.
- Ask everyone to stand in a neutral position-feet shoulder width apart; arms down at their side; head straight.
- Tell the group that they are not to initiate any movement intentionally, but that they should vigilantly mirror the person that they have chosen to watch. Start the game.

Questions:

What happened as you were mirroring your partner? How did this feel? Was there a time you felt you forced your actions?

NOTE: *we are doing this activity to build up our energy, team spirit, encourage creativity especially through improvisation and communication among partners.*

The Mad Chicken[3]

Facilitator, say:

- We are all going to count from 1 to 8 whilst shaking our right hands in the air.
- Let's repeat the count whilst shaking our left hands.
- Then let's count to 8 whilst shaking first the right leg and then the left.
- We will repeat these 4 actions, but this time only count up to 4.
- Repeat, counting to 2.
- Finally count only to 1, repeating this last part twice.

Q & A

Q: Why are we doing this?
A: this is a good exercise because it is quick and helps generate energy. You should encourage everyone (mothers and their children) to take part in this activity.

[3] Adapted from the 'Dissociate coordinated movements' (Boal, 2005).

The Checklist

> ***NOTE:*** *As you plan your performances, here are some of the topics you can include in your plays, poetry and song acts*

The Checklist (what to include)

- **Stunting**: Make a play that shows the difference between a stunted child and one who is not stunted. Why should we care? What are the benefits?
- **Cognitive development:** how does a healthy child think? Are they able to solve problems or not whether in school or at home?
- **Language development:** language delay or non-delay? For instance, a mother talks to the child another doesn't. One says good things to the child another does not.
- **Physical development/ social**: how can play help with a child's physical development? Is there a difference in motor development between an active and an inactive child?
- **Social emotional growth:** a child living in an abusive/ neglect filled home versus a child living in a happy home, is there a difference?
  - A healthy mother equals a healthy child

> **NOTE**: *All the information included in the performances should be in line with what was learnt. If you are not clear, feel free to consult the Child Development Agent (CDA) in your area.*

PREPARING FOR YOUR PERFORMANCE: REHEARSAL TECHNIQUES.

Facilitator:

- Use the acting techniques below to prepare your spect-actors[4] for the final show.[5c]

a. Talking with numbers/ gibberish press conference

- Actors substitute dialogue with numbers/ gibberish.
- Mothers we will pair up and take turns speaking in numbers or gibberish.
- *Variation: an interpreter (facilitator can do this or another member) can be availed. Here members of the group can ask questions, which the interpreter will translate into gibberish and vice-versa.*

[4] Theatre of the oppressed term that refers to the audience that's now been converted into 'actors.'

[5] Refer to Barton, R. (2009) Acting: Onstage and off, 5th edition. Wadsworth, Cengage learning: Boston, for more rehearsal techniques. Boal (2005) Games for actors and non-actors, Babbage (2004) Augusto Boal and Michael, J., Michel, J. & Silverberg, J. (2001) Ready for action: A popular theatre/popular education manual, are very helpful sources in this regard [rehearsal for participant preparation and performance development].

b. Stop and think

- During the scene, the facilitator shout 'STOP AND THINK'.
- All the mothers then freeze and say their character's thoughts out loud. The facilitator then shouts 'CONTINUE'.
- This exercise helps the actors to understand their motivations for action.

- *Variation: facilitator stops the action and asks 1 particular character 'what are you thinking now?' The actor responds in character and tells the rest of the group what the character is thinking. Then the scene continues.*

c. Changing Characters

- The actors exchange characters with each other and then rehearse the scene (especially those characters who have a relationship e.g. husband and wife, father and son, boss and worker).
- The actors don't have to learn each other's lines, just give a general idea of the character and the action.

d. Speed run/ Slow motion

- Rehearse the scene as quickly and with as much energy as possible. No lines or actions should be cut.
- Rehearse the scene in slow motion without leaving out any dialogue or action.

e. Ask a Silly Question

Facilitator:

- Quickly fire off silly questions and call on participants to answer them as spontaneously as they can.
- For example, "What does a goat wear to bed?" "How do people cook nsima in a tree?" "Why does a rat snore?" "How many eggs can a pig lay?" "How does a cow sing?" etc.
- There are no wrong answers, save not saying something.

NOTE: *The activities above are helpful in developing improvisation skills such as listening and awareness, storytelling, spontaneity, trust, creativity, etc. Though no script is necessary, a story spine (outline and theme) must be present.*

Using the above Rehearsal skills, Lets make our performance.

NOTE: Rehearse final performance from beginning to end

The String[d]

Materials: a ball of string (sack string can be used in this case)

Facilitator, say:

- Let us sit in a circle.
- One mother will give the ball of string to someone in the circle. She will hold onto its end and throw the rest of it across the circle to another mother (or child).

- She will then give a message to the person who catches the string.
- In this case, the message can be a comment about the lessons (something she has liked or disliked), a message for the person to take away from the group, a hope for the future or her hopes for her child, herself, her family or her community…
- The receiver then holds onto the string and throws the ball across the room to another player.
- The process continues until everyone is holding onto the string, which will have made a spider's web pattern.
- At the end of this session, the last person (or facilitator) will cut all the string connections between players.
- Everyone can keep their own piece of string to remind them of the message we received during these meetings.

Good day exercise [the final act][e]

- Greet or hug each other and tell each other something nice for the day. Here, each mother hugs another mother and says positive things about the other. For instance, when Jane greets Mary, she will say, 'Hi my name is Mary and I am very kind.'
- Then May will return the favour, by saying her name is Jane and…....

Have a lovely season

[a] Boal, A (2005) Games for actors and non-actors, second edition. Taylor & Francis online publication.
[b] Boal, A (2005) Games for actors and non-actors, second edition. Taylor & Francis online publication.
[c] Barton, R. (2009) Acting: Onstage and Off, Fifth edition. Wadsworth, Cengage learning: Boston.
[d] Moyo, G. (2014/15) Theatre for Development. Lecture notes
[e] Boal, A (2005). Games for actors and non-actors, second edition. Taylor and Francis online publication.

Additional Materials

Activity List

Materials Checklist

Participant's Manual

ACTIVITY LIST: WE START AT TWO[1]

1. DRESS-UP TIME

Materials needed: old clothes, old shoes, and vitenges.

What to do: Take out a pile of old clothes and let your child play dress-up. You can play with them but it is great to encourage group play with two or three other children their age. Initially the mother can play with the child, then allow the child to attempt this task on their own and later with peers.

Skills learned: Creativity, imagination, language skills, and social development

2. A LITTLE CONVERSATION

Materials needed: Doll

What to do: Hand over the doll, and encourage your child to hold, talk, dress, and take care of it. Talk to the doll the way you would talk a child, and encourage your child to do the same.

Skills learned: Social, language and fine motor skills, creativity and imagination

3. TOY HIDE-AND-SEEK

Materials needed: Toy

What to do: Hide a toy somewhere in the house, and ask your child to find it. Explore with her, using cues like "warmer" and "colder" to guide her.

Variations: Use a torch for the search, or hide several objects at one time.

Skills learned: Listening, problem solving, social skills, and memory

[1] All activtities except those specified where derived from Sturm Niz, E. (n.d.) expert recommended activities that will enhance your 2 year old's development. Retrieved from: http://www.parents.com/toddlers-preschoolers/activities/indoor/activities-for-two-year-olds/.. Please note that almost if not all tasks have been adapted to suit the context of study except 'Simon says'

4. SIMON SAYS

What to do: Start out with simple directions-"Simon says, touch your toes"-then graduate to silly, more complex routines ("Simon says, tug on your left ear, then your right ear"). And don't forget to drop "Simon says" every now and then!

Variations: You can also encourage your child to jump, skip, catch something, and more.

Skills learned: Gross motor skills, following directions and receptive language

5. YOU'VE GOT MAIL

Materials needed: Old box or a bottle even a calabash, junk mail (stones, sticks, leaves, a paper with a drawing on it, clay doll_ or animal)

What to do: Make a mailbox by decorating an old cardboard box and cutting a slit in the top or use a calabash. Fill it with goodies together with your child. Then ask your child to draw out certain goodies. Talk about the pictures, colours, and shapes; help her or him sort it by size, shape or colour; or count the pieces. You can also layer in imaginative play by pretending these are things you bought at the market.

Skills learned: Develops an understanding of basic concepts, fine motor skills

6. ALL ABOARD

Materials needed: Towel or blanket, basket

What to do: Have your toddler sit in the basket on a towel or blanket and gently pull her around the room. Pretend the blanket is a train or a boat and that you are stopping at different places, like the zoo or wherever your imaginations take you.
Here you can also pretend to be a horse, and let your child sit on your back as you crawl around the house. Make sure you are both safe, start slowly.

Skills learned: Balance, pretending

7. BODY TRACING

Materials needed: Large piece of paper, charcoal

What to do: Have your child lie down on a large piece of paper and trace the outline of his body. "Because the child has to lay still to be traced, he learns self-control." You can show him or her where the two eyes, nose, and mouth go, but if your child just wants to color all over it, that's fine. Don't impose anything on him, just let him or her have fun with it."

Variation: If your child doesn't want to lie still, don't force him or her . Start with tracing just his or her hand or foot, or tracing your hand and foot.

Skills learned: Sense of self, self-control, and identifying body parts (cognitive & language skills).

8. STOP! GO!

What to do: Play a game that involves starting and stopping. Use your hands as your child's guide. For example: palm facing upwards and outwards means STOP and palm waving back and forth (sideways) means GO! Try this as many times as possible.

Skills learned: Self-control (socio-emotional development)
Developing self-control will eventually help children negotiate, compromise, and work out conflicts without losing their temper.

9. BUTUME ACTIVITY[2] (erranding)

Materials needed: none specific as long object is safe

What to do: First, tell your child to go and sit near an object [child should be free to play with object if he or she choose to. Do not to scold her or him for that. Then, tell the child to bring

[2] As directed by primary author, refer to lesson 3 for more details.

the object and give it to her. Ask child: what is it? (Referring to object) What is it used for? Do you want to share it with me? If child says yes, s/he wants to share then caregiver should show positive affect (happy facial expressions) and praise the action. For instance: Oh wow, thank you very much. If not, you can say: Oh that's okay. I see you still want to play with it. Positive facial expressions and gestures should be shown here too. Then ask child to share or take the object to another child or familiar adult. Here you simply observe the interactions that will happen.

> **NOTE**: *Variations in activity can be made but keep the activities simple. Encourage caregivers not to worry if child does not do this task exactly during the first few tries. Remember he or she is only two years old, however, overtime the child will be able to do it. This task is a gate way for erranding and encourages internal verbalisations (thinking or reasoning) in the child as well as socialisation.*

10. MAKANI AJILO[3] (Stories from the Past)

What to do: Talk to your child about two events that happened no longer than 4 weeks ago. For instance, talk about the time grandma came to visit or when you went to the market or visited a relative or when a relative came to visit. you can also talk about events that happened when you were with your child or those that happened when you were not with your child. You can talk about things that happened to you or you can talk about what you saw. Make facial and hand gestures as you talk to your child. You can make funny faces, make sounds (e.g. cow sound or cat's sound, or how daddy sounded). Be in character, pretend to be the person or the cow. Make this conversation as visual as possible. This is how children learn, by looking at your face and connecting with the words.

Skills learned: language use, and social expressions

11. RESPONSIVE FEEDING[4]

Materials needed: two spoons and tree bark or sack string

What to do: You can tie two spoons tightly together by their handles using a string or tree bark so that when the child eats you can also use the other end to feed herself. You tie two spoons together. As you feed, your child you can pretend that the spoon is a train trying to deliver goods (the food) into a special destination (the child's mouth). Be excited, happy. This task encourages the child to eat as mummy is doing it too.

[3] As described in Tulviste et al (2013). Read lesson 4 for more details.
[4] Adapted from Castle, J. (n.d.). Refer to lesson 4 for more details.

Skills learned: mother learns to read hunger and satisfied cues of the child, while the child learns how to express themselves facially and later on verbally.

Have a Lovely Day...
