# Supplementary material for: Two-year impact of community-based health screening and parenting groups on child development in Zambia: Follow-up to a cluster-randomized controlled trial
Source: PLoS Med. 2018 Apr 24;15(4):e1002555. doi: 10.1371/journal.pmed.1002555 (PMC5915271; doi:10.1371/journal.pmed.1002555)
Supplement: S2 Text — (DOCX) [file pmed.1002555.s007.docx]

**Improving Early Childhood Development in Zambia**

Protocol version 5.0

March 14, 2016

**Principal Investigators:**

Davidson H. Hamer, MD

Zambia Centre for Applied Health Research and Development (ZCAHRD)

Plot 121, Kudu Road, Kabulonga,

P.O. Box 30910,

Lusaka

Email: [dhamer@bu.edu](mailto:dhamer@bu.edu)

Cell phone: +260973543773

Godfrey Biemba, MB ChB, MSc

ZCAHRD

Plot 121, Kudu Road, Kabulonga,

P.O. Box 30910,

Lusaka

Email: gbiemba@gmail.com

Cell phone: +260974770293

**Co-investigators:**

Peter Rockers, ScD

Department of Global Health

Boston University School of Public Health

Günther Fink, PhD

Department of Global Health and Population

Harvard School of Public Health

Arianna Zanolini, MSc, PhD

American Institutes for Research

Kebby Musokotwane, MD, MPH

PMTCT Coordinator, Ministry of Community Development, Mother and Child Health

Vichaels Silavwe

Ministry of Community Development, Mother and Child Health

Contents

Summary 5

1 Background and Introduction 7

1.1 Rationale for the study 7

1.2 Statement of the problem 8

1.3 Significance 8

1.4 Aim 9

1.5 Specific objectives 9

1.6 Ethical issues 11

2 Literature Review 12

2.1 Early childhood education 13

2.2 Current interventions 14

3 Methodology 14

3.1 Study design 14

3.1.1. Sampling 14

3.1.2 Internal validity 15

3.2 Determination of clusters and randomization 15

3.3 Study interventions 16

3.4 Eligibility Criteria 18

3.5 Study Procedures 19

Recruitment and baseline survey 20

Routine visits in intervention clusters 21

3.6 Assessment of malnutrition screening 23

3.6.1 Assessment of health facility current practices and standards of care 24

3.7 Assessment of perceptions and behavioral changes triggered by mother groups through focus groups. 25

3.8 Sample Size Calculations 26

4 Ethical considerations 27

4.1 Risks for participants 27

4.2 Informed Consent 28

4.3 Data Security and Management 29

4.3.1 Instrument development 29

4.3.2. Data capture/paper storage 29

4.3.3 Data management 30

5 Data analysis 31

6 Funding & Budget 33

7 Time Frame 33

8 Reference List 35

# Summary

Despite major national and international efforts, Zambia continues to struggle with a high burden of under-nutrition and infectious disease. In Southern Province, an estimated 36% of children under-5 years are stunted, and about one in ten children die before reaching age 5 [[1](#_ENREF_1)]. Poverty is widespread, and maternal education low with 34% of women aged 15-49 years not having completed primary schooling [[1](#_ENREF_1)]. Malnutrition, infectious diseases, poverty, poor maternal education are all major risk factors preventing children to reach their full potential and harming children in a critical phase of development [[2](#_ENREF_2)]. Lack of investment in early childhood has been shown to have long-lasting negative effects on adult health, psychosocial wellbeing and economic stability [[3](#_ENREF_3),[4](#_ENREF_4)]. Early childhood interventions in cognitive stimulation, child health, and nutrition have provided higher returns than most other social programs, showing that early childhood is both a cost-effective and cost-efficient time to invest [[5](#_ENREF_5),[6](#_ENREF_6)]. Investments in early childhood can alleviate the negative effects of poverty and at the same time promote economic growth.

In this study we propose to assess the operational feasibility and developmental impact of an integrated community-based early childhood development (ECD) program. Under the program, each targeted community will be assigned a Child Development Agent (CDA) trained by the program. The CDA will have three main responsibilities: 1) Supporting parental access and use of essential child health services, such as vaccinations, growth monitoring and vitamin supplementation; 2) Ensuring prompt treatment of acute diseases such as malaria, diarrhea, and pneumonia by referring caregivers and their children to the nearest community-based provider or primary health center; and 3) Organizing weekly meetings for mothers and caregivers to discuss parenting issues and promote early childhood cognitive stimulation.

Given the information gathered in the first year of the study, we also propose to extend for a second year the role of the CDA, but only limited to 3) Organizing and mentoring the mother groups.

The program builds on previous research done in the Southern Province, where 39,787 mothers were enrolled through pregnancy and the neonatal period in the Zambia Chlorhexidine Application Trial (ZamCAT). This cluster randomized trial was designed to compare the impact on neonatal mortality and omphalitis of dry cord care with daily 4% chlorhexidine gluconate cord washes. While this study ended in December 2013, we plan to use the excellent relationship that ZamCAT established with the community, traditional leadership and district health offices to work again in these communities for the currently proposed ECD trial. CDAs will be trained on issues of child health and development, and will be supported by a data tracking system, which will be used to both monitor the development progress of participating children and to remind parents of upcoming health checkups including routine vaccination programs.

Objective and Relevance: The objective of the proposed study is to improve the physical and cognitive development of young children by reducing their exposure to undernutrition and infectious diseases, and by increasing mother-child access to early stimulation programs. We hope that the results of this study will further increase the interest of both researchers and policy makers in early childhood development in general, and specifically in the first 1,000 days of a child’s life.

**ZCAHRD**

The Zambia Centre for Applied Health Research and Development (ZCAHRD) is a non-governmental registered in Zambia under the Society’s Act. ZCAHRD’s vision is to be a leading health institution in applied and operations research, programs evaluations, innovative service delivery, and health capacity development in Zambia.

ZCAHRD’s strategic approaches include conducting evaluations of public health interventions to measure health outcomes, impact and efficiency of public health services, conducting and supporting applied research on relevant health and development questions, strengthening the capacity of local scientists and trainees from Boston University’s affiliated programs to undertake applied research on health and development and strengthening health services through collaboration with the government of Zambia, international donors and local partners.

ZCAHRD is composed of faculty and staff of the Center for Global Health and Development (CGHD) at Boston University, USA and Zambian public health professionals and specialists who are working together to carry out applied policy and program-relevant research on critical issues of public health importance. The goal is to generate evidence to support health policy decisions that will improve public and well-being in Zambia. In addition, ZCAHRD works closely with the Ministry of Health (MoH) and the Ministry of Community Development, Mother and Child Health (MCDMCH) to support the scale up of quality health services in government health facilities in Southern Province. In recognition of ZCAHRD’s support to the MoH, a memorandum of understanding has been signed between MoH and ZCAHRD in order to formalize the relationship.

# Background and Introduction

## Rationale for the study

A growing literature has highlighted the important effects of early childhood risk factors on physical and cognitive development [[2](#_ENREF_2),[7](#_ENREF_7),[8](#_ENREF_8)]. Even though brain development is fairly robust in principle, it can be modified by early life environment, nutrition, illness and other experiences [[9](#_ENREF_9)]. Many newborn children survive perinatal health problems and prematurity, but are at risk of long-term deficits. Nutritional deficiencies and repeated infections can form a “vicious cycle”, reinforcing each other, and undermine the physical and cognitive development of children, with subsequent effects on education, and economic productivity of individuals and their families [[5](#_ENREF_5),[10](#_ENREF_10),[11](#_ENREF_11),[12](#_ENREF_12)]. With an estimated 200 million children worldwide not reaching their developmental potential [[2](#_ENREF_2)], an urgent need to develop new interventions to improve child health and development has emerged.

## Statement of the problem

Common childhood illnesses such as malaria, pneumonia and diarrhea result in undernutrition and stunting; delays in linear growth as well as the lack of cognitive stimulation, especially in rural areas of Zambia, lower the long-term potential and productive ability of children. Neurocognitive and/or physical deficits can be prevented by ECD programs and community-based strategies designed to educate primary caregivers on the importance of good health, nutrition and cognitive stimulation for optimal holistic development of their children.

Zambia currently does not have national or large scale programs tackling contemporaneously health, nutrition and socio-emotional development of very young children. A growing body of research suggests that these interventions may be particularly effective in improving short and long-term deficits in countries with high malnutrition prevalence due to the deep interconnection between health, nutrition and socio-emotional development. However, evidence on interventions is still scarce and programs implemented vary substantial across countries We propose to pilot and then rigorously evaluate a community-based, integrated intervention addressing nutrition, health and socio-emotional development specifically tailored to the Zambian context. If proved to be effective in boosting child development, this intervention could be highly cost-effective and easily scalable in Zambia. The knowledge generated by this study may also be used by the government to review policies and programs surrounding ECD.

## Significance

The proposed intervention is to our knowledge the first early childhood program in Zambia that will rigorously evaluate a combined health, nutrition and early life stimulation intervention. If the proposed intervention shows both feasibility and impact, the program has the potential to be incorporated into existing national community-based health initiatives and to substantially improve the wellbeing of children in Zambia and other resource-limited countries.

## Aim

The aim of this study is to demonstrate the feasibility and preliminary impact of an integrated community-based early childhood intervention to improve the health, nutritional status, and cognitive development of young children.

## Specific objectives

**Specific objective #1:** To conduct a cluster-randomized controlled trial to determine whether a community-based package of interventions targeted at early childhood development (ECD) and delivered by community development agents (CDAs) can reduce stunting and improve cognitive and motor development by two years of age.

The study will have two primary outcomes:

1. **Prevalence of stunting (height-for-age z-score (HAZ) < -2) at age 2.**
2. **Cognitive and motor development by age 2 years as measured with a standardized test developed as part of the multinational INTERGROWTH-21^st^ study** [[13](#_ENREF_13)]. The assessment tool combines elements of four existing testing instruments (Bayley Scales of Infant Development (BSID), the Rapid Neurodevelopment Assessment (RNDA), the Malawi Development Assessment Tool and the Griffith’s Scales) and simultaneously assesses cognition, language, motor skills and behavior.

**Specific objective #2:** To determine whether the intervention improves effective access to child health services. This will be measured by the following secondary outcomes:

- Vaccination rates
- Vitamin A supplementation
- Utilization of routine child health check-up services

**Specific objective #3:** To determine whether the intervention alters caregiver interactions with children. We will use the *Home Observation for Measurement of the Environment* (HOME) inventory [[14](#_ENREF_14)]. This will be measured by the following secondary outcomes:

- Availability of books, toys and other reading materials at home
- Time spent by caregivers reading to the child
- Time spent by caregivers playing with the child

**Specific objective #4:** To determine whether there are secondary benefits of mother/caregiver-led-support groups in terms of maternal mental health. This will be measured by the following secondary outcomes:

- Maternal depression, captured with the SRQ-20 questionnaire, a test validated internationally including in neighboring Malawi[[15](#_ENREF_15)]

**Specific objective #5:** To determine the types and quality of nutrition services currently provided in the five health centers and referral feeding centers. This will be collected via a structured KII with nutritionist or clinical care specialists at the national, provincial and district levels. Questions will focus on:

- Current policies and guidelines relating to child health and nutrition

**Specific objective #6:** To determine the relationship between the 5 selected health facilities’ quality (measured and perceived) and parents’ use of services for malnourished children. Both perceived and parents’ use of services will be collected via questions collected on baseline data regarding satisfaction to health facility services. Facility quality will be determined by a structured KII with health facility staff. Questions for health facility staff will focus on:

- Health worker knowledge on treatment of malnourished children
- Health facility screening procedures
- Quality of nutrition services
- Audit of clinic resources

**Specific Objective #7:** To assess the usual diet of 6-12 month old infants. We will use a form that utilizes the 24-hour recall method to measure quantities and types of foods consumed by the child in the past 24-hours as reported by the mother.

- Measure by 24-hour recall, time and food or beverage consumed
- Aid of measuring cups, food pictures and diagrams

**Specific Objective #8:** Describe and document the perceptions of mothers on mother groups, as well as any behavioral changes in parenting or in mother support networks resulting from participation in mother groups.

This will be done through focus group discussions and analyzed with qualitative analysis methods.

**Specific Objective #9: A**ssess parenting behavior prior to the start of the mothers’ group extension, in April 2016.

We will visit all study households (intervention and control) in April 2016 and collect information on a short set of parenting behaviors. We have attached the instrument we will use. The parenting behaviors we will assess are the same as those assessed during previous household surveys for this study. We will also obtain a new informed consent form from all study households at this visit.

**Specific Objective #10:** Assess the impact on parenting practices and primary outcomes after extending the mother groups component for an additional period of time (7 months).

While the assessment of cognitive development at less than 2 years of age is very difficult and is and not-precise, the assessment closer to 3 years-old can be more reliable. We will continue the mothers’ groups component of the study (parenting) for 7 additional months, then re-assess the cognitive development of the children as well as parenting practices.

## Ethical issues

All procedures will be conducted according to local standards of routine clinical care. This protocol, the informed consent documents, and any subsequent modifications will be reviewed and approved by all relevant ethics committees responsible for oversight of the study including ERES Converge in Zambia and the Boston University Medical Campus IRB. All staff having contact with participants will receive training on the protection of human research participants prior to conducting any study activities.

We expect participant risk to be minimal for mothers and children. Specific risks for mothers and children as well as informed consent procedures are explained in detail in Section 3.7 below.

# Literature Review

The importance of early child development on individual welfare in later stages of life has been well documented in developed countries. On average, children with inferior cognitive abilities during early childhood do worse in school, receive less education, earn lower wages, and are also significantly unhealthier in later life [[4](#_ENREF_4),[5](#_ENREF_5),[16](#_ENREF_16),[17](#_ENREF_17)]. Similarly large welfare effects are typically found in relation to childhood health. Gluckman and Hansen[[18](#_ENREF_18)] highlighted the importance of *in utero* health on later health and development in the Barker tradition [[19](#_ENREF_19),[20](#_ENREF_20)]. Smith[[21](#_ENREF_21)] showed a strong correlation between early childhood health and welfare throughout life using the older cohorts followed in the health and retirement study (HRS). Case et al.[[22](#_ENREF_22)] used longitudinal data from the UK to show the importance of both uterine environment and early childhood health on children’s educational attainment and welfare during adulthood. These long-term studies do not generally allow a direct identification of the causal pathways from childhood health to educational attainment and welfare. However, they do generally point towards children’s cognitive and physical development as key drivers of the observed correlations. A notable exception in this field is recent work by Salm and Schunk [[23](#_ENREF_23)], who used a complete set of child health records from Germany to show that ill health during early childhood explained between 18 and 29% of the cognitive differentials of children at age 6, strongly affecting children’s educational and labor market outcomes in the long run.

*Rationale for an integrated intervention:* One of the key limitations of most EC programs is the focus on a single domain of child development. Recent research on brain development suggests that it is not single risk exposures or developmental stressors that cause most harm, but rather the toxic stress generated through the accumulation of early childhood adversity [[24](#_ENREF_24),[25](#_ENREF_25)]. Given this evidence, single risk factor interventions are, despite their beneficial effects on child development, unlikely to achieve the highest possible impact on child development.

In this project, we propose to overcome the limitations of existing intervention programs by implementing a comprehensive integrated, community-based child development program, designed to simultaneously address the most critical challenges to child development during the first two years of life in developing countries today.

## Early childhood education

Evidence on the developmental effects of early childhood education programs on children in sub-Saharan Africa remains relatively scarce. Several recent studies conducted in Kenya, Zanzibar and Uganda [[26](#_ENREF_26),[27](#_ENREF_27)], Botswana [[28](#_ENREF_28)], South Africa [[29](#_ENREF_29)] and Cape Verde [[30](#_ENREF_30)] suggested positive effects of early childhood learning initiatives; rigorous evaluations of such programs are not generally available.

At a regional level, net enrollment rates in early childhood programs remain low [[31](#_ENREF_31)] and very little is known about the quality and content of the early childhood training provided. Rao and colleagues [[32](#_ENREF_32)] found that disadvantaged children in India who attended higher-quality preschool programs experienced greater developmental growth than those attending poor-quality programs. However, the India study as well as recent evidence from Cambodia suggest that *any* early childhood education experience is beneficial to children [[33](#_ENREF_33),[34](#_ENREF_34)].

In Zambia, school enrollment at lower grades is now near-universal and gross enrollment ratios frequently exceed 100% at the basic school level [[35](#_ENREF_35)]. However, many students enter primary school at age 8 or later, and dropout rates remain high, with less than 50% of students progressing to secondary schooling [[36](#_ENREF_36)]. With severely constrained overall educational resources, the Zambian public early childhood education sector has remained largely underdeveloped: it lacks a national curriculum and policy framework, and suffers from a shortage of qualified teachers. There is also poor coordination among key stakeholders to facilitate holistic implementation of early childhood programs [[37](#_ENREF_37)]. Overall, Zambian children's exposure to early childhood programs appears limited. Only 17% of new first-graders reported any early childhood care and education experience in 2010 [[38](#_ENREF_38)], while the projection for the year 2015 stands at 30% [[37](#_ENREF_37)].

## Current interventions

While several projects targeting early childhood have been launched, most interventions in Zambia are limited geographically and not currently integrated into larger education or health programs. UNICEF has been piloting an integrated intervention jointly implemented with Save the Children and PLAN; the materials developed for this initiative will be used as a basis for the intervention to be tested in our pilot study.

# Methodology

## Study design

The study is designed as a two-arm cluster-randomized trial with the aim of demonstrating the feasibility and preliminary impact of an integrated community-based early childhood intervention to improve the health, nutritional status, and cognitive development of young children. The study will take place in the Southern Province of Zambia.

Through an existing research program (ZamCAT), nearly 40,000 pregnant women and young infants have been monitored during pregnancy and until 42 days of the young infant’s life since 2012. This existing research network will be used for this trial.

### 3.1.1. Sampling

A total of five health facilities with a corresponding number of 30 health zones will be selected for the study. All primary caregivers (mothers or other female caregivers) with children between 6 and 12 months of age will be invited to participate in the study for a total of 500 caregiver-child pairs. Details of how participants will be recruited are provided in Section 3.5.

For evaluation purposes, catchment areas of each health facility will be divided along the already established neighborhood health zones with 4-6 zones per clinic. Fifty percent of all zones at each facility will be randomly chosen for the ECD program. The random assignment of zones within each facility to the ECD program will allow us to rigorously assess the program’s impact and effectiveness.

### 3.1.2 Internal validity

Power calculations indicate that the selected number of health zones will be sufficient to detect the expected impact on our primary outcomes. After randomization, the soundness of the randomization as a procedure to control for confounding will be checked by comparing variables obtained through the baseline survey and through community level data. Examples of baseline variables that will be compared are maternal age, education and occupation; child age and gender; birth weight of the child; duration of breastfeeding length; and initial vaccination status. We will also include community level baseline variables such as distance to health center and District Medical Office, road access and presence of community health workers. Although blinding of treatment will not be possible due to the very nature of the intervention, we do not expect Hawthorne effects to be significant in this case due to the length of the program. We also do not foresee important concerns due to differential attrition in the two arms because of the long-standing relationship of ZCAHRD in all of these communities (both the control and intervention) established through the previous ZamCAT study.

## Determination of clusters and randomization

Through the recent ZamCAT research efforts, the study team has established a close partnership with district health offices in six districts in Southern Province (Mazabuka, Siavonga, Monze, Choma, Kalomo and Livingstone). In total, 90 facilities were part of the recently completed ZamCAT. Since we aim for a target population of about 500 children, we will choose five facilities with approximately 100-150 children in the 6-12 months age range for the study.

From each of the five facilities, we will randomly select 50% of zones for the program, and use the remaining 50% as a control (comparison) group. The selection of zones will be based on a random number draw. We will select health facilities with six zones each for the study, which means that we expect to have 15 zones in our treatment group and 15 zones in our intervention group. We will randomize health facility zones with stratified randomization with strata based on population size and distance to the health facility; this will help ensure balance in health characteristics of the health zones and populations.

## Study interventions

Communities selected for the intervention arm will benefit from a new cadre of health workers, which will be recruited and trained for the sole purpose of monitoring and supporting all aspects of child development under the age of 2. The newly trained health workers will bear the title of “child development agents” (CDA) and will be community-based. A total of five CDAs will be trained and employed for the project period; their sole responsibility will be to support targeted children and their caregivers with the interventions outlined below. We will ask for community recommendations for the selection of CDAs with the requirement that they have some previous experience in health-related activities and ability to read and write in both English and Tonga, the local tribal language. The CDAs will be employed by the project and work full-time. Each CDA will be responsible for approximately 50 intervention children across three health zones. **CDAs will visit all intervention children on a bi-weekly basis**. In total, CDAs are expected to conduct 100 household visits per month (50 children times two visits each); this will mean that they will each do about five visits per day. **The home visits will have two fundamental functions:**

1. **Ensuring treatment of malnutrition and acute infections**. CDAs will monitor children’s growth using mid-upper arm circumference (MUAC) measurements, if they identify children that have moderate acute malnutrition (defined as absence of edema and MUAC <12.5 and ≥11.5 cm), they will provide supplemental information to the caregiver on how to improve the child’s diet. If the CDAs identify children with severe acute malnutrition (defined MUAC <11.5 cm without edema or MUAC of <11.0 – 12.5 cm with pedal edema), they will be advised to immediately refer the child to the nearest health facility for further evaluation and management. Similarly, CDAs will inquire about symptoms of malaria, acute diarrhea or pneumonia, and instruct parents to seek treatment at the nearest health facility as needed.
2. **Monitoring utilization of routine health services.** Zambia’s Ministry of Health has developed a comprehensive well child program over the past years, including standard vaccines (BCG, DPT-Hib, and polio), vitamin A supplementation, deworming with mebendazole, and clinical health checkups. However, in many cases, there is limited uptake of these services in rural communities. The CDA will remind caregivers of critical milestones for child development and support parental efforts to receive routine health services as advised on the national “Child Clinic Card (formerly known as the Under 5 Card)” all caregivers receive. To facilitate the aforementioned process, we will support CDAs with the provision of a cell phone. The cell phones will have three functions: 1) to automatically remind CDAs of upcoming health services for each child; 2) to track visits and service utilization from an operational perspective; and 3) to allow CDAs to contact their supervisor to ask questions and to obtain feedback on whether a child should be referred to a hospital or other project-related questions caregivers may have.



**In addition to the bi-weekly home visits, the CDA will be responsible for organizing and supporting community-based early life learning and stimulation activities.** Similar to the programs successfully implemented in Cambodia [[39](#_ENREF_39)], CDAs will directly work with mothers and caregivers^[[1]](#footnote-1)^ from each community, and support volunteer mothers (who become ECD community leaders) with instructions and learning materials. Volunteer mothers will be provided with flip charts, which contain lessons on the importance of mother-child interaction, on how to talk to the child and on how to maintain child health. The volunteer mother will be instructed to host weekly meetings for other mothers and caregivers in her community and lead a collegial discussion on one of the topics in the flip chart. This approach has been found to be just as effective in boosting cognitive development as more formal and more expensive community-based schools [[39](#_ENREF_39)].The main strength of this approach is that mothers learn to have an active role in their child’s growth; this empowerment effect has been shown to increase in a continuous way their investment in their children [[40](#_ENREF_40)]. We expect most of the changes in parenting and cognitive development to come from this intervention.

**Intervention in comparison group:**

In the comparison group communities, we will refer any children that during the surveys are identified with fever, cough/difficulty breathing, diarrhea, or SAM/MAM to the nearest health center for evaluation. Otherwise the comparison clusters will continue with the current Ministry of Community Development, Mother and Child Health standard of care. No CDAs will be assigned in the control health zones, and no mothers groups will be organized in the control communities.

## Eligibility Criteria

Inclusion

- Children aged 6-12 months in the catchment areas of the five selected health facilities in Southern Province will be eligible to participate
- Child’s primary caregiver must be 15 years or older
- Child’s primary caregiver must be a female (because the participants in the women’s group may feel uncomfortable discussing certain issues if a man is present)
- Health workers working at the five health centers where the study is taking place
- District and provincial level nutritionists or clinical care specialists

Exclusion

- Caregivers who are unwilling to provide informed consent
- Families that plan to move from their health center catchment zone during the twelve month period of the study
- Health workers, nutritionists, or clinical care specialists who refuse to provide informed consent

## Study Procedures

Training of CDAs

Prior to the initiation of the study, the CDAs will participate in a one-week training workshop, which will consist of a mix of didactic and practical sessions. This will include an overview of the study design, description of the study interventions, research ethics and protection of human participants, screening for signs of common infectious diseases and danger signs, and extensive training on the use of the anthropometric toolkits, as well as the comparison of anthropometric measurements with the age-specific reference tables.

CDAs will also receive special training on community-based early cognitive stimulation. The early childhood development training will be based on the UNICEF/WHO curriculum and on the Save the Children Essential Package training. This is the same training that UNICEF and Save the Children have adopted in Zambia after obtaining local ethical clearance and Ministry permission. The workshop will include a careful review of the process of informed consent, importance of confidentiality, and detailed review of the baseline and routine data collection instruments. We will also conduct a post-training test to assess the understanding of study procedures and competence of the CDAs in conducting interviews and performing anthropometric measurements.

Pre-testing data collection instruments

The baseline survey and biweekly data collection forms will be pre-tested with 10 households (caregivers) to assess the clarity of the questions and ensure that appropriate responses are available on the form. After pre-testing the forms will be finalized before the initiation of data collection.

###

### Recruitment and baseline survey

Study staff will visit all villages within the catchment area (both intervention and control zones) of the selected health facilities at the beginning of the study. The study will be introduced to local health authorities, traditional leaders, community leaders, and health center committees. After making sure that the study’ goals and procedures are acceptable to the traditional leaders, community leaders, and health center committees, the CDA will identify potentially eligible children aged 6-12 months through the health center under 5 registries and the village headman’s log book. The CDA will work with the local traditional leaders to locate the families of potentially eligible children. The primary caregiver of the child (hereafter referred to as the caregiver) will be approached, the screening questions will be asked and, if the child is eligible, the caregiver will be asked to provide consent (or assent if the parent is under 18 years old with consent to be provided by the husband if the mother is a minor). Once informed consent has been obtained, the CDA will schedule a time to conduct the baseline survey.

The CDAs will conduct a baseline survey of both control and intervention households which will include: a) basic demographic and socioeconomic status, b) availability of books, toys and other reading material at home, time spent by caregivers reading to the child, as well as time spent by caregivers playing with the child, c) anthropometric screening (weight, height, and MUAC), d) review of vaccinations and child health visits using child health card, e) assessment of maternal or caregiver mental well-being, f) assessment of maternal depression with the SRQ-20 questionnaire, and g) presence of other early childhood development programs in the community. If caregivers report any danger sign during the baseline survey, they will recommend immediate referral to the health facility. If any signs of uncomplicated illness (suggestive of malaria, pneumonia, or diarrhea) are present, the CDA will liaise with the nearest volunteer community health worker (CHW) or community health assistant (CHA), if available in the catchment of the clinic, and refer the child to this person for evaluation. If there is no CHW or CHA in the health zone, the child will be referred to the health center for evaluation. Caregivers will also be asked if they had previously participated to ZamCAT study, and if they would be willing to have the researchers access their baseline data from ZamCAT. Before obtaining permission to review their ZamCAT baseline data, we will obtain written informed consent to access these data.

Households in control health zone clusters will undergo the baseline and endline surveys including an endline assessment of early childhood development. Routine MOH heath facility and community-based activities will continue but none of the aforementioned study interventions will be implemented in the control areas.

Tracking and monitoring system

At enrollment, a unique ID and visit schedule will be created for each child and their caregiver. Each CDA will keep a log of the children that she visits fortnightly. The log will include the ID of each child, the date when the child was visited, and whether there was any referral. The study coordinator will then visit the CDA every two weeks to conduct monitoring activities, record the number of successful and unsuccessful visits, check that the report matches the biweekly forms, verify completion of the biweekly forms and to conduct random monitoring visits to one of the mothers in the intervention arm to verify that the visits have been conducted as reported. The coordinator will also generate a fortnightly report with statistics for the study team to monitor and countercheck with the data coming from the biweekly visits

Whenever there is need for a referral to the health facility as explained in the section below, the CDA will set up a paper based tracking system. She will complete a referral form and hand it to the mother, who will bring it to the health facility during her referral. The health facility staff will then fill in the remaining part of the referral form with diagnosis, treatment and date of visit. The CDA will then pass by the health facility in the following days and collect the form, to confirm that the mother has visited the health facility with her child. The number of referral forms is then recorded during the coordinator’s fortnightly visits.

### Routine visits in intervention clusters

Upon completion of enrollment and the baseline survey, enrolled mothers in areas selected for the intervention will be visited by the CDAs. Routine visits will focus on:

1. *Health and Nutrition Monitoring*

Each visit in intervention clusters will start with a review of the child’s health status and an update since the previous visit. CDAs will have previously reviewed the child’s under-5 card and will have reminders on which children are behind with their vaccinations and check-ups. At each visit, the CDA will review the child health card with the caregiver, and inform and refer to the clinic if the child has missed an immunization, health check-up or a routine treatment such as deworming or vitamin A supplementation. The CDA will also remind the mother of upcoming routine health check-ups for the child. The CDA will measure the child’s MUAC[[41](#_ENREF_41)] every month and communicate the result to the mother. If the result shows signs of moderate to severe acute malnutrition or a poor growth pattern, the CDA will refer the child to the clinic. Following WHO/UNICEF guidelines [[42](#_ENREF_42)], we will classify all children as severely malnourished if their MUAC is less than 115 millimeters (11.5 centimeters). In case of malnutrition, the CDA may also work with the mother to suggest an affordable, local, and nutritionally appropriate diet for the child following Ministry guidelines and the ones of the Zambia National Food and Nutrition Commission. The CDA will also ask about presence of acute infectious diseases such as fever, diarrhea, cough or difficulty breathing, worms and refer to the clinic and to a community-based health worker such as a CHA or CHW if any of these are present. The routine visits for health and nutrition monitoring will last for 12 months.

1. *Cognitive Stimulation/Parenting Intervention facilitation and check-up.*

CDAs will also ask local headman for their support in finding volunteers for the community-based early childhood learning groups. Volunteers will be trained to lead the community-based parenting intervention (for being the “head mother”) by the CDA, who will supervise and mentor the volunteers. This training will occur on a regular basis during the CDAs biweekly visits to the local community. Before the beginning of the study, head mothers may also receive half a day of formal training. Volunteer mothers will receive the training material and the informational material that will be the core of each meeting by the CDAs. The “head mothers” will be responsible for organizing a meeting at least once a week with all of the other interested mothers, and especially caregivers of children who are part of the study. During each session the mother will lead a discussion on the information material representing the topic of that session, and will be in charge of stimulating a discussion. There will also be homemade toys created with local material or other sessions where specific songs, rhymes, or games will be taught. The CDA will attend the parenting group on a regular basis, but not each week, to monitor the quality of the information that is given. Every time the CDA visits a community, she will also visit the head mother to refresh her training for the following lessons, to troubleshoot any day-to-day problems, to make sure that the meetings are ongoing and to answer to any questions. During routine visits with the caregivers in the study, the CDA will also review the material that was discussed at that week, and give the caregivers the opportunity to ask questions. The parenting program is set to last for 9 months. We anticipate that it will start around month 3 of the intervention.

An example of the early childhood development curriculum is attached in Appendix B. We have 25 weekly classes that are focused on introducing the course, cognitive development, maternal health and wellbeing, socio-emotional development, communication, gross motor development, fine motor development, rights and protection, nutrition, hygiene and child health. Over the course of the ECD curriculum, both theory and practical applications of the health and development messages will be taught. Some practical applications include toy making and learning new songs.

**Endline Assessment**

Twelve months after enrollment all children will be visited for a final follow-up interview and assessment. This assessment will be done by a separately trained team of child assessors, and will include the questions asked at baseline as well as an assessment of child development using a standardized test, the INTERGROWTH 21^st^ century assessment tools.

**Extension of mother groups and follow-up assessment**

We propose to extend the mother groups component of the study to increase exposure to the parenting component of the study. Preliminary results indicated encouraging results from the mothers’ group component in terms of behavior change and engagement in the child development of their children. Mothers would like to continue the intervention and we propose

to formally support this extension. At the end of the extended period, we propose to re-assess the

cognitive and socio-emotional development of children in the intervention component. This will

allow us to measure in a more precise way and also after increased exposure to the program, both

of which will increase the power to detect a change in cognitive and socioemotional development. We will also re-assess parenting practices to observe any long-term follow-up in terms of parenting practices and engagement. The results from the first year indicate that Intergrowth might not be the best assessment tool, and we propose therefore to use the gold standard, the BSID-III.

## Assessment of malnutrition screening

To obtain a qualitative overview of how the national malnutrition guidelines are being implemented at the provincial and district levels, we plan to conduct a survey interviewing nutrition and/or clinical care specialists at different levels of the health system (district, province, and national).

To assess the quality of nutrition services at facilities, we propose to conduct structured interviews with the clinic staff (e.g. to assess health worker knowledge of malnutrition treatment, health facility screening procedures, and management for malnutrition) and conduct an audit of clinic resources, (e.g., the number of people that have access to the facility, staffing levels, equipment, and availability of RUTF).

To understand parents’ perceptions and use of services for a potentially malnourished child, we will use household interview data being collected as part of the study baseline procedures (e.g. if your child is stunted or not gaining weight, what services are available or what will you do; do you know of local or home services available?).

## Assessment of health facility current practices and standards of care

Health facility assessment: Participants will be nurses certified in treatment of severe acute malnutrition or in–charges (managers) of participating health facilities which will includes health centers, both in the public and mission run.

Health worker interviews: The participants will be clinical staff providing malnutrition treatment in the participating health facilities on the day of the survey.

- - 1. **Assessment of dietary intake**

During a bi-weekly visit, the CDAs will perform a one time 24-hour recall to assess the food consumption of all intervention children in five selected health facilities. CDAs will be trained on interviewing techniques, how to elicit detained descriptions of food and beverage items, and how to estimate and record and record portion size. Portion sizes will be estimated via modeling clay molded into the correct size and shape of the food, portion-sized photographs of foods and standard measuring cups.

## Assessment of perceptions and behavioral changes triggered by mother groups through focus groups.

Mother groups for parenting are new in Zambia, and no study has studied their dynamics. We would like to understand and document mother perceptions, behavioral changes and changes in the support network related to mother groups through a qualitative study using focus group discussions. Qualitative research allows researchers to investigate process changes and other themes that cannot be captured by the endline quantitative assessment by having participants identify channels, challenges and themes related to the mother groups, and inform future implementation of mother groups.

Focus group discussions will be conducted to determine the perceptions of the mothers on the mother’s groups, mother’s perceptions on their behavior change since attending the mother groups and their feelings of belonging, social capital and if they have established any social networks since they began attending these groups. We will conduct:

- 4 focus groups with mothers participating in the study and attending mother groups, each group with 6-9 mothers invited. Focus groups will be done at the health facility and will be approximately one hour in length. Mothers will be recruited by facility, with the exception of one focus group that will recruit mothers from two facilities. Each mother will be selected from a different mother group to maximize privacy and make the mothers more comfortable in talking about their group. For each mother group, the study ID of one mother will be randomly selected. The selected mother will be approached by the CDA; if the mother is not interested in participating, another mother will be randomly selected.
- 3 focus groups with mother heads participating in the study, each with 4-7 participants. Mother heads are study participants who volunteered to head the mother groups. Sampling will be divided in a first group including the first two facilities, a second group including the other two, and the last facility by itself because too isolated. For each sample strata, we will randomly select 6 mother groups and invite their mother head for the FGD.
- 1 focus group with CDAs (which we consider key informants) with all of the 10 CDAs.

Informed consent for focus groups. The focus groups will record age, parity, marital status, education and attendance, but this information will not be linked to facility catchment area nor to any other information collected as part of the main study. No other personal health information or personal data will be used nor collected. The focus group discussions will be minimal risk and, as a result, we will use verbal rather than written consent using a verbal script form instead of a written informed consent form.

Data collection for focus groups: All the staff involved in focus group discussions will be trained in confidentiality and research ethics. Interviews will be done in either Tonga or English, and recorded using a digital recorder. No last names nor village nor facility will be mentioned during the recorded session. Sequential IDs will be given to participants for referring to each other.

The following steps will be followed: a) explanation of information sheet and informed consent process; b) introduction; c) explanation of the process; d) logistics; e) ground rules and emphasis on confidentiality; f) collection of age, parity, marital status, education and attendance for each FGD ID; g) turn on tape recorder and conduct FGD following FGD guide; h) turn off tape recorder, thank the mother; give refreshment and transport reimbursement.

## Sample Size Calculations

The study is designed to detect increases in standardized child development scores of 0.5 standard deviations with power = 0.9. The main assumptions underlying this calculation are an average group size of 20 children in each of the 30 zones, and an intra-class correlation coefficient of 0.1 (a design effect of 2.9). Similarly, the study is powered to detect an increase in height-for-age z-scores of 0.5 standard deviations in the intervention arm relative to the control arm. We aim to enroll 250 children in each study arm for a total sample of 500 children. In order to account for lost to follow-up, we aim to recruit up to 5% additional children per study arm (262 per arm).

For the year two follow-up assessment, we are powered to detect an increase in standardized child development scores, as measured by the BSID-III, of 0.3 standard deviations with power 0.8.

Sample size for health facility surveys and feeding centers is seven. There will be 10 health workers interviewed (assuming 2 per health facility), 5 national, provincial and district level nutritionists or clinical-care specialists and the feeding sample size for assessment of dietary intake will be all willing and enrolled intervention children (estimate 275).

# Ethical considerations

## Risks for participants

We expect only minimal risks for children and their caregivers. All study procedures will follow official MOH guidelines for routine child care and child health services. The main purpose of the CDA visits will be to ensure that children are receiving health services developed and scheduled by the MOH. The CDAs **will not** provide medical care to the children, but will refer children to the health clinic when necessary or to the nearest CHA/CHW. The CDAs will give advice on nutritional issues based on MOH guidelines. All of the CDAs will be formally trained in the nutritional and health on the specific messages that they are allowed to communicate. All CDAs will also receive training on the protection of human research participants prior to conducting the study activities.

We consider a minimal psychological risk of embarrassment for the caregivers who have not followed the MOH guidelines for child health visits, and get a reminder from the CDA to bring the child to the clinic. We will minimize this risk by integrating into the CDA training a specific part emphasizing the need for respect and empathy, and avoiding judgmental comments to the caregivers.

We also consider risks for breach of confidentiality to be minimal. All efforts will be made to maintain confidentiality by securely storing the questionnaires and the data from the routine health visits in a safe, locked location (see subsection “Data Security and Management”).

Economic risks are minimal. CDAs will make all efforts to reach the caregivers in moments when they are not working. Caregivers may visit the clinics more often than what they would have been without intervention, which will cost them transportation money. However, no caregiver will be coerced to go and the recommended frequency of routine health care visits will only be within the MOH recommendations for visiting the health facility.

The cognitive/parenting intervention will also have no more than minimal risks. The curriculum for parenting stimulation has been approved by UNICEF and it is largely overlapping with another intervention that UNICEF is implementing in 4 other provinces of Zambia, which has already been approved by local IRBs and by the Ministry of Education. The curriculum is based on basic, safe, uncontroversial and culturally appropriate messages for child development such as basic hygiene messages (handwashing, breastfeeding), quality mother-child interaction messages that encourage the mothers to talk with their child, label objects, sing songs, and make home-made toys (see Study Procedures above). The community-based nature of the group, and the fact that mothers will teach each other rather than having a model strictly imposed or recommended, will further guarantee culturally appropriate practices and discussion. We expect no more than minimal risk for stigma or embarrassment for mothers following practices that were not recommended. The groups are supposed to represent a safe environment amongst peers to discuss and understand the importance of positive practices rather than judgment of negative ones. The CDAs will reinforce this message with the head mother, and will also routinely monitor the groups and signal to the investigators any behavior or dynamic that may generate embarrassment or stigma.

Any unforeseen research-related injury to participants will be borne by the research team.

## Informed Consent

As described above, all caregivers will be informed about the project, and caregiver-child pairs be enrolled only if they provide written informed consent to do so (see Participation Information Sheet and Consent Form attached).

We will ask for a new informed consent before the April 2016 parenting assessment (see Parenting Assessment Information Sheet).

Health workers will be informed about the project in which the interviewer will explain the purpose and rationale of the study and will inform the participants that they will not be paid for participating, they are not obliged to participate, and they can refuse to answer some of the questions. They will be assured of confidentiality regarding any information they provide. They will be asked to sign the consent form, and will be offered the opportunity to receive a copy of the consent form. Only after written informed consent is provided will a participant be permitted to participate in the KII.

## Data Security and Management

## Instrument development

Standardized data collection tools will be designed in TeleForms® (see below for more information) and will be linked to a Microsoft Access database. Instruments will be piloted in the study area and changes will be made as appropriate. These corrections will include alterations for clarity, consistency and spelling mistakes.

## Data capture/paper storage

Identifying information including the woman's full name, location of her home and cell phone number (if applicable) will be kept in a study log book that will be carried by each of the CDA and will be kept separately from the medical information about the study participants. All study forms will only contain study identification numbers and will not contain any identifying information. Field monitors will use standardized forms for all data capture at baseline, midline and endline.

At enrollment, a unique study ID will be given to the caregiver and all forms will use this ID to link the forms together. The study ID will have 5 digits. The first two digits will indicate the cluster (health facility) and the final three digits will be the caregiver’s unique identifier. This will be a continuous number, advancing by 1 for each mother enrolled: i.e. 01001, 01002, 01003, 02001, 02002, 02003, etc.

Data collected at enrollment will include contact information for the mother (address/cell phone). As each mother is enrolled, a timeline will be developed for that mother-infant pair. The timeline will be sent to the CDA and reminders near/around the day of the visit will be given to the CDA to follow-up with the mother.

Data collected at baseline and endline will include information on maternal and child health, weight, height, mid-upper arm circumference (MUAC), as well as neurocognitive assessment (endline only) with standardize questionnaires. At the end of baseline and endline assessment, the data collectors will meet with the field supervisors daily to hand in their paper forms.

For the monthly monitoring, data on children in the intervention arm, including MUAC and presence of danger signs of illness, will be collected. The CDA will meet with a data supervisor every two weeks to hand in all monitoring data on the child.

Field supervisors will review the forms for errors/missing data and will ask the CDA or data collector to fix the errors if possible. Hard copies of the data forms will be taken to the main office (Choma), scanned into a PDF and data captured through the TeleForms program.

All paper files will be stored in the central office in Choma in file drawers, ordered by study ID number. The files will be locked and the field director will have keys to those cabinets. At the end of the study, the paper files or electronic images will be stored up to 5 years past the end of the study.

## Data management

Comprehensive data management and data cleaning will occur in real-time as data is accrued. Over the past 10 years while working in Zambia, we have successfully utilized the TeleForms® system for data verification and entry. This approach offers a high-volume, high-accuracy data capture and management system. Teleforms® enables hand-written text to be translated to computer readable files and then data is entered into a Microsoft® Access database. As Teleforms® includes a data verification system, the need for multiple data entry personnel is eliminated.[53]

A data entry specialist with his/her dedicated scanner, faxed in files, and computer will scan the paper forms, import the faxed forms, and all fields will be verified through the TeleForms® system; this approach acts in a manner similar to a double data entry system. An image file of each paper form is created in scanning. These image files will be backed up to an external hard drive. Data from the TeleForms® system, including the images, and the Access databases will be locked on password-protected computers in the field office. The Access database will be e-mailed as a password-protected file back to the study epidemiologist on a monthly basis.

Monthly data checking will take place with verification of the Access database with the paper forms by the Choma-based data manager. Of all paper records, 10% will be randomly chosen and compared with the Access database each month. Additionally, data cleaning will involve logic checks, range, missing data, and missing form checks. Data queries will be sent from the central office to the data supervisor and the respective field monitor for correction. The data entry specialist and data manager will maintain a data query log; only the data manager in the field will make changes in the Access database.

# Data analysis

We will use the following primary outcomes:

1. Proportion of children with stunting (height-for-age Z-score less than 2 standard deviations below the mean);
2. Child development scores using the INTERGROWTH tool;

Other outcomes of interest include:

1. Proportion of children receiving all age-appropriate vaccines
2. Proportion of children receiving vitamin A supplementation;
3. Number of well-baby visits attended;
4. HOME score;
5. Average time spent reading with the child;
6. Average time spent playing with the child;
7. Proportion of women scoring as depressed, based on the SRQ-20.

We will investigate the relative risk associated with random group assignment as a fixed covariate predicting each outcome using generalized estimating equations (GEE) models that take into account the clustering by health facility. We will use an exchangeable correlation structure in the model.

We will compare baseline characteristics between the two groups that may be related to the outcome, but we expect randomization to have created reasonably balanced groups. Categorical variables will be compared between groups using the chi-square test or Fisher’s exact test if cell sizes are small, continuous variables using t-tests if normally distributed or non-parametric Wilcoxon rank sum tests, if non-normal. We will adjust for confounders as appropriate.

Potential confounders include: maternal education, paternal education, socio-economic status, baseline nutritional status, and presence of food insecurity. The process of randomization should help to balance confounders across the two study arms. Crude and adjusted risk ratios will be presented with 95% confidence intervals in appropriate tables.

**Analysis of objectives 5-7**

Hand written notes will be taken by the interviewer in addition to an audio recording of the interview. The notes will then be coded for common themes and outliers. For analysis of KII data, we will focus on individual views and will, where relevant, look at the proportion of total respondents who report particular views. To the greatest degree possible, this analysis will begin during data collection or shortly after the KIIs are completed in order to permit participation in the analysis by the interviewers who conducted the KIIs and therefore who will be most familiar with the information collected.

At the beginning of the study, a one time, one-day dietary intake will be collected by each CDA prompting the mother or caregiver to complete a 24-hour food recall of their child’s food and drink intake as described in the GAIN working Paper Series. (43) Descriptive frequencies of food intake and proportions of food groups represented will be examined and comparative analysis to WHO guidelines will be performed.

**Analysis of objectives 8: perceptions and behavioral change on mother groups**

Data analysis for focus groups: Focus group discussions will be transcribed and analyzed using NVIVO or similar qualitative analysis software. Emerging common themes and channels will be highlighted. Quotes (without names made, nor facility mentioned) will possibly be included in the manuscript.

**Analysis for Objective 9:**

The parenting assessment will provide information on how parenting behaviors have changed since the last assessment in October 2015. We will primarily look at the number of activities that parents indicate completing with the child.

**Analysis for Objective 10:**

At the year two follow-up, we will measure the following primary outcomes:

1. Proportion of children with stunting (height-for-age Z-score less than 2 standard deviations below the mean);
2. Child development scores using the BSID-III;

Other outcomes of interest include:

- 1. Child diet diversity
  2. Caregiver-child interactions
  3. Proportion of caregivers scoring as depressed, based on the SRQ-20.

# Funding & Budget

The study is funded through the *Grand Challenges Canada’s Saving Brains: Scaling Impact* project. Total project budget is Canadian $ 250,000.

The extension for mother groups and the follow-up assessment is funded through the DFID Policy Research Grant for a budget of approximately US$ 163,000.

# Time Frame

| Development of intervention materials and BU IRB/Zambian ethics committee approvals | Sept 2013 – April 2014 |
| --- | --- |
| Surveyor and CDA recruitment and training | April 2014 |
| Pre-testing data collection instruments, enrollment and baseline survey | May 2014 |
| Intervention rollout | September 2014 |
| Endline evaluation | October 2015 |
| Data analysis | November 2015 |
| Year 1 report and data dissemination | December 2015 |
| Parenting assessment | April 2016 |
| Mothers group extension | April 2016 |
| Year 2 (follow-up) evaluation | November 2016 |

# Reference List

1. Zambia Central Statistic Office, Zambia Ministry of Health, Tropical Diseases Research Centre, University of Zambia, Macro International Inc. (2009) Zambia Demographic and Health Survey 2007. Calverton, Maryland, USA: CSO and Macro International Inc.

2. Grantham-McGregor S, Cheung YB, Cueto S, Glewwe P, Richter L, et al. (2007) Developmental potential in the first 5 years for children in developing countries. Lancet 369: 60-70.

3. Maluccio JA, Hoddinott JF, Behrman JR, Martorell R, Quisumbing AR, et al. (2006) The Impact of Nutrition during Early Childhood on Education among Guatemalan Adults. University of Pennsylvania Population Studies Center Working Paper 06-04.

4. Heckman J, Stixrud J, Urzua S (2006) The effects of cognitive and noncognitive abilities on labor market outcomes and social behavior. Journal of Labor Economics 24: 411-482.

5. Cunha F, Heckman JJ (2007) The technology of skill formation. American Economic Review 97: 31-47.

6. Heckman JJ (2007) The Economics, Technology, and Neuroscience of Human Capability Formation. Proceedings of the National Academy of Sciences 104: 13250-13255.

7. Shonkoff JP, Richter L, van der Gaag J, Bhutta ZA (2012) An integrated scientific framework for child survival and early childhood development. Pediatrics 129: e460-472.

8. Leung BM, Wiens KP, Kaplan BJ (2011) Does prenatal micronutrient supplementation improve children's mental development? A systematic review. BMC Pregnancy Childbirth 11: 12.

9. Hertzman C, Boyce T (2010) How Experience Gets Under the Skin to Create Gradients in Developmental

Health. Annual Review of Public Health 31.

10. Victora CG, Adair L, Fall C, Hallal PC, Martorell R, et al. (2008) Maternal and child undernutrition: consequences for adult health and human capital. Lancet 371: 340-357.

11. Hoddinott J, Maluccio J, Behrman J, Flores R, Martorel Rl (2008) Effect of a nutrition intervention during early childhood on economic productivity in Guatemalan adults. Lancet 371: 411-416.

12. Heckman JJ (2007) The economics, technology, and neuroscience of human capability formation. Proceedings of the National Academy of Sciences of the United States of America 104: 13250-13255.

13. Villar J, Altman DG, Purwar M, Noble JA, Knight HE, et al. (2013) The objectives, design and implementation of the INTERGROWTH-21st Project. BJOG: An International Journal of Obstetrics & Gynaecology 120: 9-26.

14. Caldwell BM, Bradley RH (2003) Home Observation for Measurement of the Environment: Administration Manual. Tempe, AZ: Family & Human Dynamics Research Institute, Arizona State Universit.

15. Stewart RC, Kauye F, Umar E, Vokhiwa M, Bunn J, et al. (2009) Validation of a Chichewa version of the Self-Reporting Questionnaire (SRQ) as a brief screening measure for maternal depressive disorder in Malawi, Africa. Journal of affective disorders 112: 126-134.

16. Currie J (2000) Child health in developed countries. In: Culyer A, Newhouse J, editors. Handbook of Health Economics North Holland.

17. Currie J (2009) Healthy, wealthy, and wise? Socioeconomic status, poor health in childhood, and human capital development. Journal of Economic Literature 47: 87-122.

18. Gluckman P, Hanson M (2006) Mismatch: Why Our World No Longer Fits Our Bodies. Oxford, UK: Oxford University Press.

19. Barker DJP (1992) The fetal and infant origins of adult disease. London: BMJ Books.

20. Barker D, Eriksson J, Forsén T, Osmond C (2002) Fetal origins of adult disease: strength of effects and biological basis. International Journal of Epidemiology 31: 1235-1239.

21. Smith JP (2007) The Impact of Socioeconomic Status on Health over the Life-Course. Journal of Human Resources 42: 739-764.

22. Case A, Fertig A, Paxson C (2005) The Lasting Impact of Childhood Health and Circumstance. Journal of Health Economics 24: 365-389.

23. Salm M, Schunk D (2008) The Role of Childhood Health for the Intergenerational Transmission of Human Capital: Evidence from Administrative Data. Mimeo.

24. Shonkoff JP, Boyce WT, McEwen BS (2009) Neuroscience, Molecular Biology, and the Childhood Roots of Health Disparities Building a New Framework for Health Promotion and Disease Prevention. JAMA 301: 2252-2259.

25. Garner AS, Shonkoff JP (2012) Early childhood adversity, toxic stress, and the role of the pediatrician: translating developmental science into lifelong health. Pediatrics 129: e224-231.

26. Reynolds AJ (2000) Success in early intervention: The Chicago Child-Parent Centers. Lincoln, NE: University of Nebraska Press.

27. Ludwig J, Miller DL (2007) Does Head Start improve children's life chances? Evidence from a regression discontinuity design. Quarterly Journal of Economics 122: 159-208.

28. Abbott-Shim M, Lambert R, McCarty F (2003) A comparison of school readiness outcomes for children randomly assigned to a Head Start program and program's waiting list. Journal of Education for Students Placed at Risk 8: 191-214.

29. Barnett WS (1996) Lives in the balance: Age-27 benefit-cost analysis of the HighScope Perry Preschool Program. Ypsilanti, MI: HighScope Press.

30. Schweinhart LJ, Montie J, Xiang Z, Barnett WS, Belfield CR, et al. (2005) Lifetime effects: The HighScope Perry Preschool study through age 40. Ypsilanti, MI: HighScope Press.

31. Malmberg LE, Mwaura PAM, Sylva K (2011) Effects of a preschool intervention on cognitive development among East-African preschool children: a flexibly time-coded growth model. Early Childhood Research Quarterly 26: 124-133.

32. Mwaura PAM, Sylva K, Malmberg LE (2008) Evaluating the Madrasa preschool programme in East Africa: a quasi-experimental study. International Journal of Early Years Education 16: 237-255.

33. Taiwo AA, Tyolo JB (2002) The effect of preschool education on academic performance in primary school: A case study of grade one pupils in Botswana. International Journal of Educational Development 22: 169-180.

34. Liddell C, Rae G (2001) Predicting early grade retention: a longitudinal investigation of primary school progress in a sample of rural South African children. British Journal of Educational Psychology 71: 413-428.

35. Pence AR, Amponsah M, Chalamanda F, Habtom A, Kameka G, et al. (2004) ECD Policy Development and Implementation in Africa. International Journal of Educational Policy, Research, and Practice: Reconceptualizing Childhood Studies 5: 13-29.

36. UNESCO (2011) Education for All Global Monitoring Report: The Hidden Crisis: Armed Conflict and Education. Paris: UNESCO.

37. Rao N (2010) Preschool Quality and the Development of Children From Economically Disadvantaged Families in India. Part of a special issue: Accountability and Quality in Early Childhood Education: Perspectives From Asia 21: 167-185.

38. Rao N, Sun J, Pearson V, Pearson E, Liu H, et al. (2012) Is Something Better than Nothing? An Evaluation of Early Childhood Programs in Cambodia. Child Development 83: 864-876.

39. Rao N, Sun J, Pearson V, Pearson E, Liu H, et al. (2012) Is Something Better Than Nothing? An Evaluation of Early Childhood Programs in Cambodia. Child Development 83: 864-876.

40. Conti G, Heckman J, Pinger P, Zanolini A (2013) Transmission of Inequality. Maternal Skills, Behavior and Birth Outcomes. Mimeo.

41. (2009) WHO child growth standards and the identification of severe acute malnutrition in infants and children: A Joint Statement by the World Health Organization and the United Nations Children’s Fund World Health Organization and UNICEF 2009.

42. WHO/UNICEF WHO child growth standards and the identification of severe acute malnutrition in infants and children. Geneva: WHO and UNICEF.

43. GAIN Working Paper Series No. 4 Applying Dietary Assessment Methods for Food Fortification and Other Nutrition Programs. GAIN, Tufts and Harvest Plus. September 2012.

1. The group is open to both mothers and primary caregivers, even when they are not the biological parent. However, for convenience it will be called “Mothers’ group”. [↑](#footnote-ref-1)
