# Supplementary material for: Two-year impact of community-based health screening and parenting groups on child development in Zambia: Follow-up to a cluster-randomized controlled trial
Source: PLoS Med. 2018 Apr 24;15(4):e1002555. doi: 10.1371/journal.pmed.1002555 (PMC5915271; doi:10.1371/journal.pmed.1002555)
Supplement: Supplementary file 7 [file pmed.1002555.s001.docx]

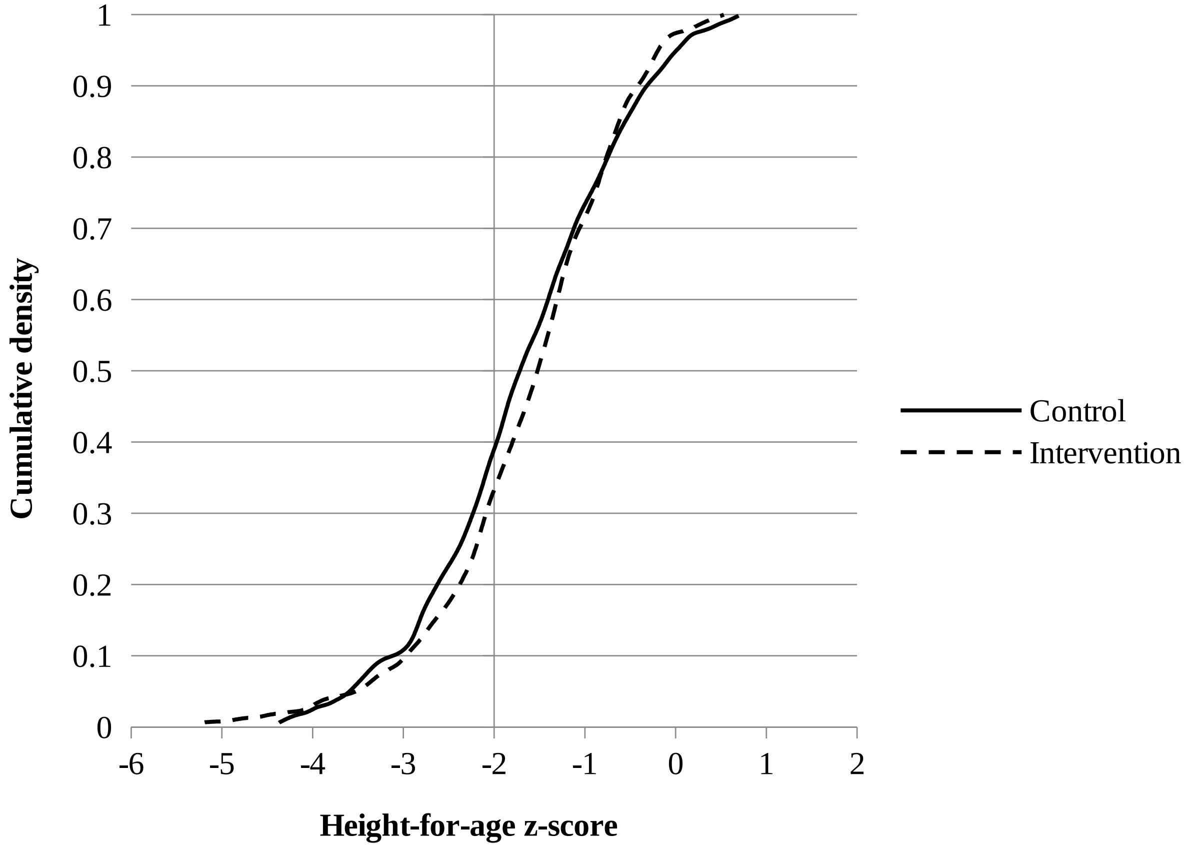
1
0.9
0.8
0.7
0.6
0.5
0.4
0.3
0.2
0.1
0
-6
-5
-4
-3
-2
-1
0
1
2
Cumulative density
Height-for-age z-score
Control
Intervention
