# Supplementary material for: Two-year impact of community-based health screening and parenting groups on child development in Zambia: Follow-up to a cluster-randomized controlled trial
Source: PLoS Med. 2018 Apr 24;15(4):e1002555. doi: 10.1371/journal.pmed.1002555 (PMC5915271; doi:10.1371/journal.pmed.1002555)
Supplement: Supplementary file 8 [file pmed.1002555.s002.docx]

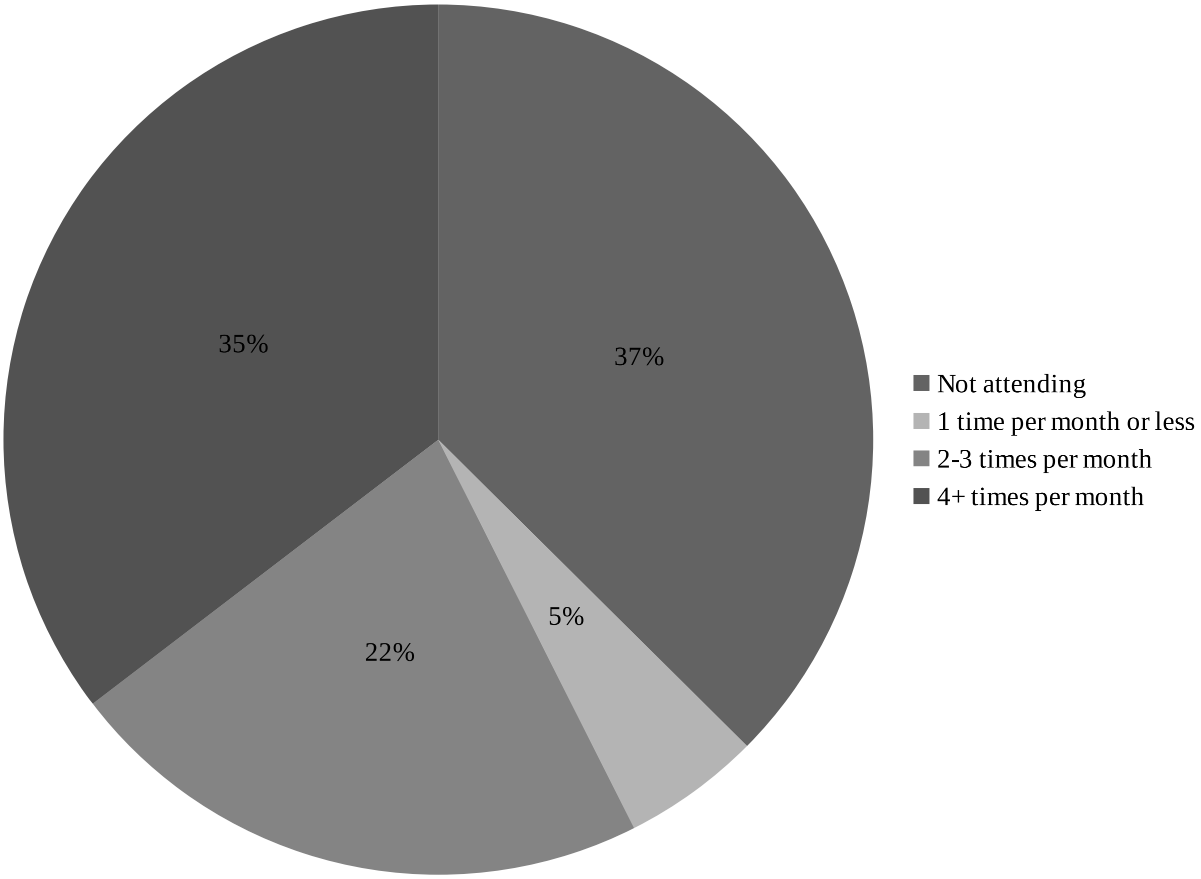
35%
37%
5%
22%
Not attending
1 time per month or less
2-3 times per month
4+ times per month
